# Through-bond *versus* through-space conjugation and high-dissymmetry chiroptical switching in proton–responsive [8]helicene bisbenzimidazoles

**DOI:** 10.1039/d6tc00671j

**Published:** 2026-03-20

**Authors:** Amira A. C. Hartmann, Vincenzo Brancaccio, Krzysztof Radacki, Holger Braunschweig, Prince Ravat

**Affiliations:** a Department of Chemistry and Biochemistry, Institute of Organic Chemistry, University of Cologne 50939 Cologne Germany pravat@uni-koeln.de; b Institut für Organische Chemie, Julius-Maximilians-Universität Würzburg Am Hubland D-97074 Würzburg Germany; c Institut für Anorganische Chemie and Institute for Sustainable Chemistry & Catalysis with Boron, Julius-Maximilians-Universität Würzburg Am Hubland D-97074 Würzburg Germany

## Abstract

We report a novel class of bisbenzimidazole-fused [8]helicenes ([8]HBIs) that integrates a configurationally stable helical scaffold with two proton-responsive benzimidazole units. Three distinct regioisomers, generated through twofold imidization of [8]helicene dianhydride, provide a modular platform for systematically tuning through-bond and through-space π-conjugation. Relative to the diimide analogue [8]helicene diimide, the [8]HBIs exhibit significantly enhanced π-delocalization, as reflected in red-shifted absorption and emission, reduced optical and electrochemical gaps, and improved photophysical performance. Crucially, reversible protonation induces helical compression and reorganization of electronic structure, resulting in pronounced bathochromic shifts in circular dichroism (CD) and circularly polarized luminescence (CPL), while preserving large dissymmetry factors (|*g*_abs_| and |*g*_lum_| up to 4.3 × 10^−2^, and 3.0 × 10^−2^, respectively) in the protonated state, outperforming the majority of previously reported acid-responsive chiroptical switches. Electrochemical data and quantum chemical calculations identify helical pitch modulation and orbital overlap as the molecular basis for these effects. These findings position [8]HBIs as versatile, stimuli-responsive materials for advanced chiroptical applications.

## Abstract 2

Bisbenzimidazole-fused [8]helicenes exhibit regioisomer-dependent π-conjugation and reversible acid–base-induced CPL switching. Protonation triggers helical compression and chiroptical shifts while maintaining strong dissymmetry.

## Graphical abstract

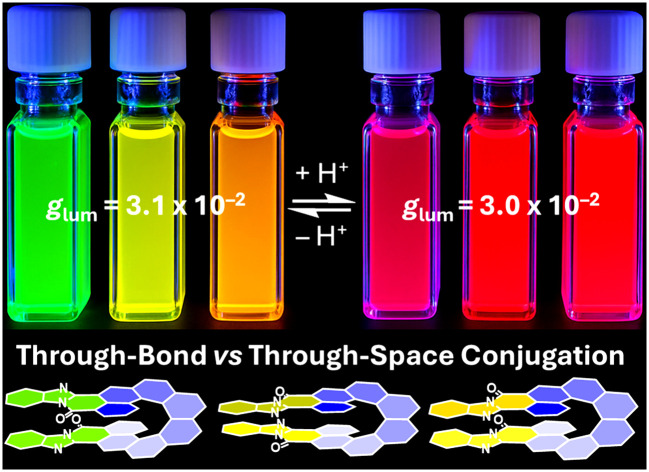

## Introduction

Effective π-conjugation governs the electronic and optical properties of organic chromophores, controlling their frontier molecular orbital (FMO) energies, redox potentials, optical energy gaps, and excited-state dynamics. In addition to through-bond connectivity, conjugation can arise from through-space interactions when π-surfaces are brought into close spatial proximity.^1–7^ The balance between these two conjugation modes is particularly relevant in nonplanar systems like helicenes,^8^ where twisted geometries naturally position distant chromophore units near each other.^9–16^ Although this interplay holds great potential for tuning electronic structure, systematic experimental approaches to modulate through-bond and through-space coupling remain underdeveloped.^17–20^

Helicenes represent archetypal chiral π-systems that combine strong electronic delocalization with configurational stability and robust chiroptical activity.^21–23^ Their helical topology yields intense circular dichroism (CD),^24^ high optical rotation, and, in some cases, circularly polarized luminescence (CPL),^25–29^ features of growing interest for chiral optoelectronics,^30–32^ spin-selective charge transport,^33–37^ and photonic devices.^38,39^ Nonetheless, optimizing both strong chiroptical activity and efficient π-conjugation remains challenging, and external control of chiroptical properties is often achieved only at the expense of configurational stability^40^ or dissymmetry.^41–44^ The development of helicene-based molecular systems^43,45,46^ that combine extended conjugation with reversible, stimuli-responsive control of their electronic and chiroptical properties, while maintaining high optical anisotropy, remains an elusive goal.^47–55^

**Fig. 1:**
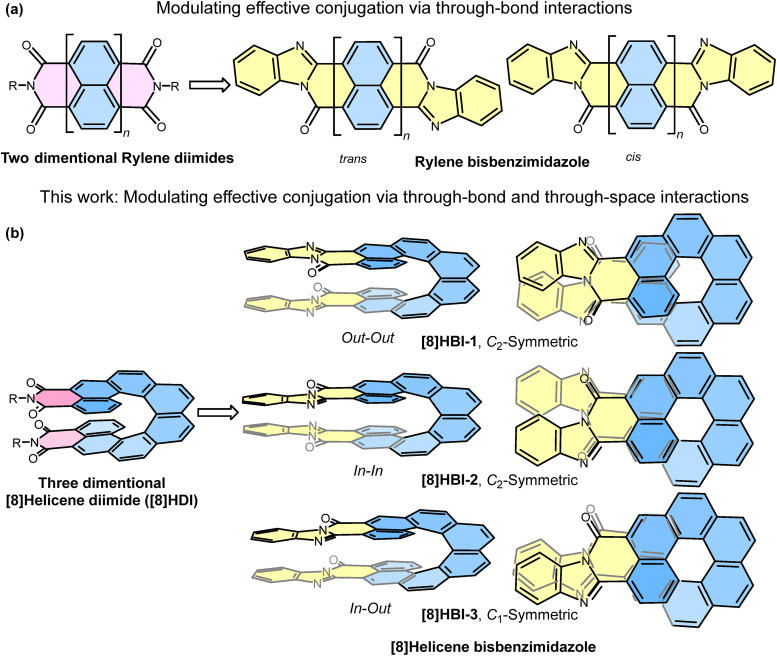
(a) Classical rylene diimides and their benzimidazole analogues. (b) A novel class of regio-isomeric [8]helicene bisbenzimidazoles ([8]HBIs), which differ by spatial arrangement of benzimidazole moieties. The side and top views are presented here for a better visualization of isomeric structures and simplified structures are shown in Scheme 1.

Rylene bisbenzimidazoles (Fig. 1a) represent a versatile class of π-conjugated dyes, historically employed as industrial pigments^56–59^ and more recently investigated as semiconducting materials in organic field-effect transistors and photovoltaics.^60–63^ A milestone in early organic solar cell research involved perylene bisbenzimidazoles, highlighting their functional potential beyond coloration.^60^ Compared to rylene diimides, their benzimidazole analogues exhibit extended conjugation, leading to bathochromic shifts in absorption and emission, smaller optical energy gaps, and higher molar absorptivity. Their synthesis is straightforward, typically involving condensation of rylene dianhydrides with aromatic diamines, yielding *cis* and *trans* isomers that display distinct coloration and semiconducting behavior.^64–66^ A prominent example is the industrial Perinone pigment,^67^ a naphthalene bisbenzimidazole derivative whose *cis* (red) and *trans* (orange) forms are both functional semiconductors.^66^ Moreover, rylene benzimidazoles are well known to undergo reversible protonation under acid/base conditions, making them promising building blocks for pH-responsive systems.^68,69^

Building on these precedents, herein we introduce a family of bisbenzimidazole-fused [8]helicenes ([8]HBIs), obtained by twofold imidization of [8]helicene dianhydride (Fig. 1b). While the imide analogue, [8]helicene diimides ([8]HDI), framework itself exhibits high optical anisotropy, we anticipate that the incorporation of benzimidazoles further extends delocalization while introducing proton-responsive functionality.^41,70^ The two-fold imidization of [8]helicene dianhydride produces three distinct regioisomers, defined by the relative placement of the benzimidazole units along the helicene backbone. The resulting regioisomeric [8]HBIs vary in the spatial arrangement of benzimidazole units, enabling a systematic study of how isomerism influences through-bond and through-space conjugation, electronic structure, and proton-triggered chiroptical switching.

Through electrochemical measurements, spectroscopic characterization, NMR titration, and quantum chemical calculations, we reveal two key findings. (1) Benzimidazole fusion enhances π-conjugation beyond that of [8]HDI, as evidenced by red-shifted absorption and emission, reduced HOMO–LUMO gaps, and mixed-valence behavior consistent with Robin–Day Class II systems. (2) Protonation reorganizes electronic communication and compresses the helical pitch, inducing large bathochromic shifts in CD and CPL spectra, while preserving large dissymmetry factors. These changes enable reversible, high-brightness CPL switching, outperforming current benchmarks for small-molecule acid/base-responsive chiroptical switches.^71^

## Results and discussion

### Synthesis and characterization

**Scheme 1:**
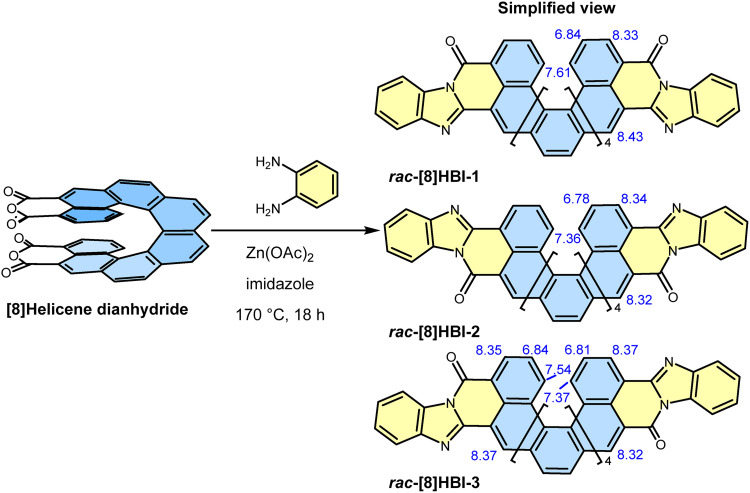
Synthesis of *rac*-[8]HBIs. Corresponding enantiopure samples were synthesized using enantiopure [8]helicene dianhydride. Characteristic ^1^H chemical shifts of the helicene core for the three regioisomers in CD_2_Cl_2_ are shown in blue.

The *rac*-[8]HBIs were efficiently synthesized in a single step *via* condensation of *rac*-[8]helicene dianhydride^19^ with *o*-phenylenediamine (Scheme 1). The reaction afforded a mixture of three regioisomers, two with *C*_2_ symmetry ([8]HBI-1 and [8]HBI-2) and one with *C*_1_ symmetry ([8]HBI-3). These were readily separated by silica gel column chromatography in a 0.25 : 1.0 : 1.2 ratio, with an overall isolated yield of 98%. The observed regioisomeric distribution arises from steric hindrance introduced after the first imide formation, which biases the subsequent annulation. This preference, driven by the differential accessibility of the inner *versus* outer carbonyl groups, can be rationalized using the buried volume analysis (SI Fig. S1 and S2). The formation of multiple regioisomers reflects the distinct orientations by which the two benzimidazole units can annulate onto the helicene backbone, a feature that enables systematic evaluation of how regioisomerism influences conjugation and chiroptical properties. Whereas rylene bisbenzimidazoles often suffer from poor solubility, complicating separation of *cis*/*trans* isomers and limiting their practical utility compared to rylene diimides, the [8]HBIs exhibit relatively good solubility in common organic solvents such as dichloromethane, toluene, and tetrahydrofuran even in the absence of solubilizing substituents. This enhanced solubility greatly facilitates both purification and spectroscopic characterization of unsubstituted [8]HBIs.

Enantiopure *P*- and *M*-[8]HBIs were obtained using the same condensation protocol starting from enantiopure *P*- and *M*-[8]helicene dianhydrides, respectively. The high configurational stability of the [8]helicene core ensures that the enantiopurity of the starting material is preserved during the reaction. The structures of all three regioisomers were unambiguously confirmed through assignment of the proton magnetic resonance signals using 1D and 2D ^1^H NMR spectroscopy (SI Fig. S29–S46) and further corroborated by single-crystal X-ray structure analysis (SI Fig. S23–S28).

### Optical properties

**Fig. 2:**
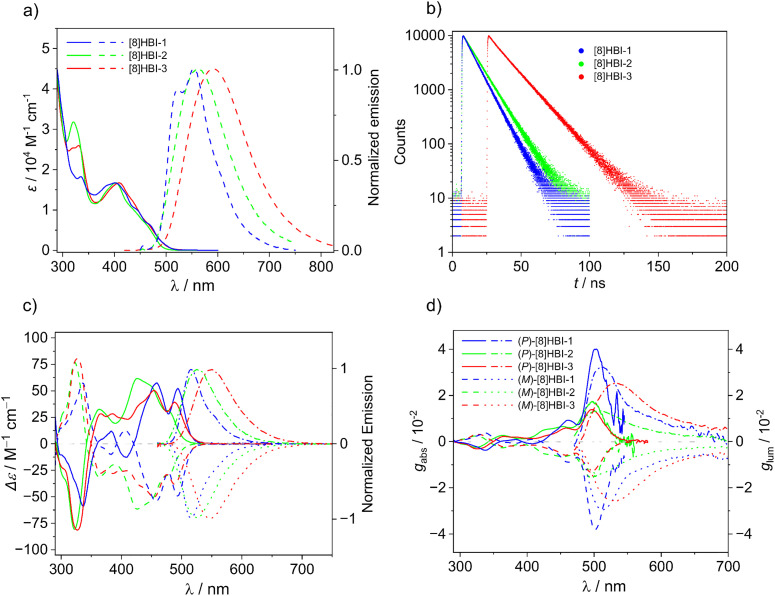
(Chir)optical properties in toluene (*c* ∼10^−5^ M) at 298 K. (a) Absorption and emission (excitation at 400 nm) spectra, (b) time-resolved fluorescence decay, and (c) CD and CPL spectra and (d) corresponding dissymmetry factors values.

The absorption and emission spectra of the [8]HBIs were measured in dilute toluene and dichloromethane (DCM) solutions and compared with those of [8]HDI (Table 1 SI Table S2). In toluene all three [8]HBIs exhibit pronounced bathochromic shifts in both absorption and emission relative to [8]HDI (Fig. 2a). Correspondingly, their optical gaps (2.48–2.50 eV) are significantly smaller than that of [8]HDI (2.63 eV), consistent with extended π-conjugation introduced by benzimidazole fusion. Although the absorption onsets of the three regioisomers are nearly identical, their spectral line shapes differ. In emission spectra, all [8]HBIs show broad bands; only [8]HBI-1 exhibits partial vibronic resolution. The emission maxima red-shift progressively across the series, with [8]HBI-1, [8]HBI-2, and [8]HBI-3 emitting in the green (553 nm), yellow (560 nm), and orange (591 nm) regions, respectively (sample pictures in Fig. 5). The pronounced redshift of [8]HBI-3 can be attributed to enhanced electronic delocalization arising from greater spatial overlap of the benzimidazole units. In DCM all three molecules exhibited a slight red shift in both absorption and emission spectra, attributed to the increased polarity of the solvent (SI Fig. S4).

**Table 1:** Summary of optical (in toluene) and electrochemical (in DCM) properties for [8]HBIs and comparison with [8]HDI

Compound	Pitch/Å	*λ*_em_/nm	*E*_g_ a/eV	*Φ*_FL_/%	*τ*_FL_/ns	*g*_abs_ b/10^−2^	*g*_lum_ b/10^−2^	LUMO^c^/eV	HOMO^d^/eV	Δ*E*_red_^e^/V
[8]HBI-1	3.44	553	2.53	20	8.79	3.9	3.1	−3.36	−5.86	0.21
[8]HBI-2	3.49	560	2.55	21	10.4	1.7	1.4	−3.40	−5.94	0.25
[8]HBI-3	3.47	591	2.48	12	14.7	1.3	2.5	−3.41	−5.89	0.23
[8]HBI-1-H_2_^2+^	3.39	638	2.26	11	3.94	4.3	3.0	—	—	—
[8]HBI-2-H_2_^2+^	3.25	638	2.29	8.0	3.47	3.7	2.8	—	—	—
[8]HBI-3-H_2_^2+^	3.40	676	2.24	7.0	3.19	2.3	2.1	—	—	—
*N*-Methyl-[8]HDI	3.28	480	2.63	4.3	5.72	3.1	3.4	−3.47	−6.09	0.22

a Optical energy gap (*E*_g_) estimated from the crossing of absorption and fluorescence spectra.

b The maximum dissymmetry factors are given. The shown values are the average of the values found for both enantiomers.

c LUMO = –(*E*_red_ + 5.10).

d HOMO = *E*_LUMO_ − *E*_g_.

e Δ*E*_red_ = Difference between the reduction potentials of the two imide moieties. –: Not measured. Data for *N*-methyl-[8]HDI are from ref. 19

The [8]HBIs also exhibit markedly higher photoluminescence quantum yields (PLQYs) than [8]HDI (4.3%). [8]HBI-1 (20%) and [8]HBI-2 (21%) show comparable quantum yields, whereas [8]HBI-3 is less emissive (12%). Time-resolved fluorescence further reveals a clear trend in excited-state lifetimes (Fig. 2b): [8]HBI-1 (8.8 ns) < [8]HBI-2 (10.4 ns) < [8]HBI-3 (14.7 ns). Together, these data indicate a systematic progression from [8]HBI-1 to [8]HBI-3 toward lower-energy emission and longer excited-state lifetimes, consistent with increasing electronic delocalization across the series.

### Chiroptical properties

The enantiomers of the [8]HBIs display mirror-image CD spectra, and their absolute configurations were assigned by comparison with TD-DFT simulations (SI Fig. S22). All three isomers display large absorption dissymmetry factors (*g*_abs_) on the order of 10^−2^ for their lowest-energy electronic transitions (Fig. 2d). Among them, [8]HBI-1 shows the largest *g*_abs_ (0.039), comparable to that of [8]HDI (0.034), while [8]HBI-2 (0.017) and [8]HBI-3 (0.013) exhibit somewhat smaller values. As in [8]HDI, these high *g*_abs_ values originate from the favorable alignment of the electric (*µ*_e_) and magnetic (*µ*_m_) transition dipole moments together with comparatively large magnetic transition dipole moments (SI Table S5).

Consistent with the CD results, the enantiomers also display mirror-image CPL spectra, with *g*_lum_ values likewise on the order of 10^−2^ (Fig. 2c). The close correspondence between the *g*_abs_ values of the lowest-energy transitions and the measured *g*_lum_ values indicates that the excited states of the [8]HBIs remain conformationally rigid during the excitation–emission process, preserving their strong chiroptical response.^72^ Importantly, dissymmetry factors of this magnitude far exceed those typically observed for small-molecule CPL emitters (10^−3^–10^−4^),^73^ underscoring the exceptional chiroptical performance of the [8]HBIs.

A comparison of the CPL and fluorescence spectra of [8]HBI reveals distinct differences in both spectral positions and the full width at half-maximum (SI Fig. S17). These discrepancies are attributed to Herzberg–Teller vibronic coupling and the constraints that limit CPL generation. While some vibronic transitions contribute significantly to fluorescence, only those with appropriate alignment and strengths of electric and magnetic transition dipole moments can effectively contribute to CPL 
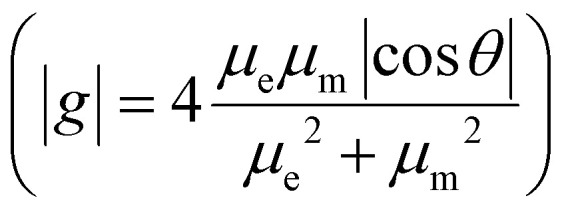
. As a result, certain vibronic transitions prominent in fluorescence appear weak or silent in the CPL spectra.^74,75^

### Quantum chemical calculations

**Fig. 3:**
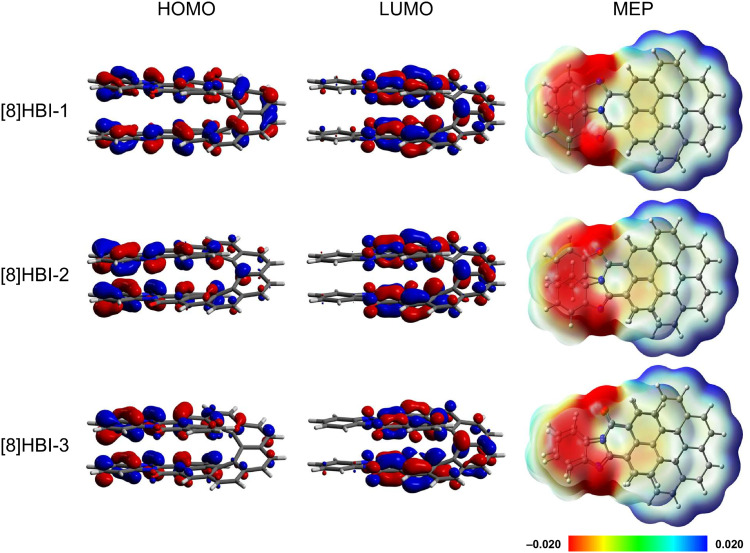
Frontier molecular orbitals and molecular electrostatic potential map (MEP) of [8]HBIs.

Quantum chemical calculations (DFT at ωB97XD/6-31G(d,p) level) provided insights into the electronic structure and optical properties of the [8]HBIs. The experimentally determined crystal structures (Table S7, Fig. S25) are in good overall agreement with the DFT-optimized geometries. However, we note that solid-state structures can be influenced by crystal packing effects (*e.g.*, racemic/enantiopure packing, intermolecular interactions, and solvent inclusion), which may slightly distort the intrinsic molecular geometry and helical pitch. Therefore, for a consistent and intrinsic structure–property analysis across neutral, radical anion, and protonated states, we have based our discussion primarily on the DFT-optimized structures. FMO analysis revealed clear differences among three regioisomers (Fig. 3). For [8]HBI-2 and [8]HBI-3, the HOMO is primarily localized on the benzimidazole units with only partial delocalization over the helicene backbone, whereas in [8]HBI-1 the HOMO exhibits significant delocalization across the helicene π-system. These variations reflect distinct through-bond conjugation pathways determined by the relative positioning of the imine linkage in each isomer. In contrast, the LUMO in all three molecules is distributed over the carbonyl group and adjacent rings of the helicene core.

Molecular electrostatic potential maps (MEP) highlight substantial electron density on the benzimidazole moieties and suggest pronounced through-space interactions, which vary with regioisomer (Fig. 3 and SI Fig. S19–S21). These features correlate with the calculated helical pitches: 3.44 Å for [8]HBI-1, 3.49 Å for [8]HBI-2, and 3.47 Å for [8]HBI-3. The variations can be rationalized by spatial interactions between the benzimidazole groups and the FMOs. In all three isomers the HOMO displays through-space antibonding interactions, but in [8]HBI-2 and [8]HBI-3 it is more strongly localized on the benzimidazole units. This enhanced localization likely contributes to the slightly larger helical pitches of these two isomers compared to [8]HBI-1.

The lowest-energy optical transitions (S_1_ ← S_0_) predicted by TD-DFT (B3LYP/6-311G(2d,p)) calculations further distinguish the regioisomers. For [8]HBI-1 and [8]HBI-3, the lowest-energy transition corresponds to HOMO → LUMO excitation, whereas in [8]HBI-2 this transition involves HOMO–1 → LUMO.

### Electrochemical properties

**Fig. 4:**
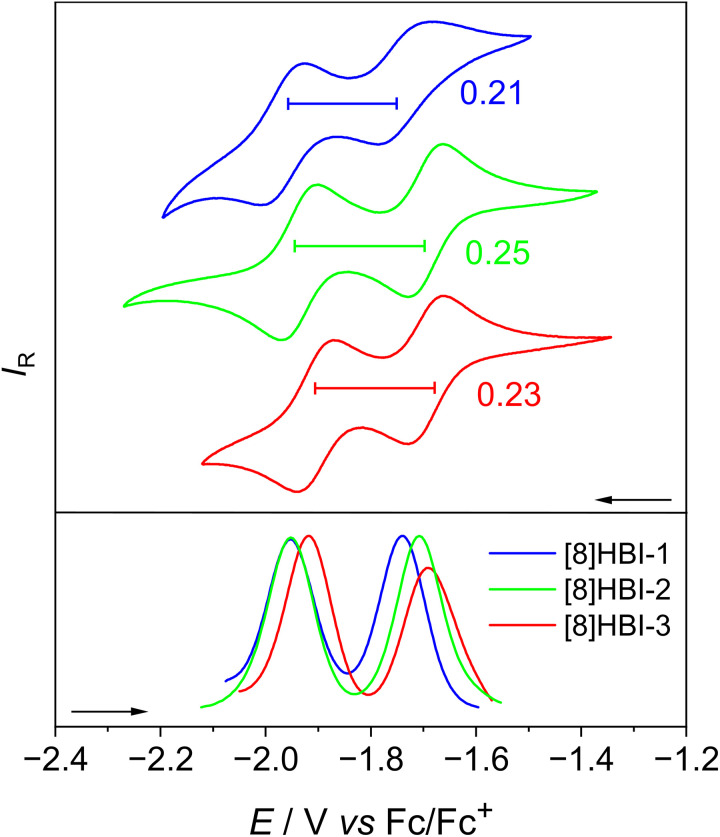
Comparison of the CV and DPV plots of the [8]HBIs in DCM with [Bu_4_N][PF_6_] (0.2 M) as supporting electrolyte at a scan speed of 50 mV s^−1^.

Cyclic voltammetry (CV) and differential pulse voltammetry (DPV) in DCM were used to probe the redox behavior of the three regioisomeric [8]HBIs and to assess electronic communication between their benzimidazole units (Fig. 4). All three isomers display two well-defined, reversible one-electron reduction waves, corresponding to sequential formation of the radical anion and dianion. The LUMO energies, derived from the first reduction potentials, decrease in the order [8]HBI-1 (−3.36 eV) > [8]HBI-2 (−3.40 eV) > [8]HBI-3 (−3.41 eV). This trend points to stronger through-space π–π overlap in [8]HBI-3, which stabilizes its LUMO, in concomitance with calculated helical pitch in neutral state. In contrast, the HOMO energies, derived from the difference of optical energy gap and LUMO energy, follow a slightly different order, [8]HBI-1 (−5.85 eV) > [8]HBI-3 (−5.89 eV) > [8]HBI-2 (−5.94 eV), reflecting the differing contributions of through-bond *versus* through-space conjugation among the regioisomers. Relative to [8]HDI (HOMO = −6.09 eV, LUMO = −3.47 eV), all [8]HBIs exhibit higher orbital energies. Importantly, their HOMO–LUMO gaps (2.48–2.54 eV) are substantially smaller than that of [8]HDI (2.62 eV) and closely match the optical gaps (2.48–2.55 eV) determined from absorption-emission spectra, confirming the enhanced conjugation introduced by benzimidazole fusion.

The separations between the two reduction waves (Δ*E*_red_), which correspond to intervalence splitting in the radical anions, also reflect the influence of molecular geometry on conjugation. The largest splitting is observed for [8]HBI-2 (0.25 V), followed by [8]HBI-3 (0.23 V) and [8]HBI-1 (0.21 V). This ordering correlates with the calculated helical pitch of the radical anions: 3.31 Å ([8]HBI-2) < 3.36 Å ([8]HBI-3) < 3.39 Å ([8]HBI-1), indicating that a more compressed helical structure enhances through-space interactions and stabilizes the reduced species.^76^ From the Δ*E*_red_ values, the comproportionation constants were calculated as 3.5 × 10^3^ ([8]HBI-1), 1.7 × 10^4^ ([8]HBI-2), and 7.7 × 10^3^ ([8]HBI-3). These large values confirm appreciable electronic coupling, and according to the Robin–Day classification, all three anionic [8]HBIs fall into Class II mixed-valence systems.^77^

### Acid/base responsive chiroptical switch

Switchable chiral molecules, whose chirality or chiroptical response can be reversibly modulated by external stimuli, are of particular interest for sensing, optoelectronics, molecular motors, and responsive materials.^47^ However, many reported small-molecule chiral switches suffer from relatively low optical dissymmetry (∼10^−4^–10^−3^) and/or limited configurational stability.^41,42,51,70,78–81^ We therefore anticipated that combining protonatable benzimidazole units with a [8]HDI core, known for its high dissymmetry factor, would yield an efficient acid/base responsive chiroptical switch.

Protonation of the [8]HBIs was first probed by ^1^H NMR titration in CD_2_Cl_2_ using deuterated trifluoroacetic acid (TFA-*d*). Progressive downfield shifts of most proton resonances confirmed protonation at the two imine nitrogens, yielding the dicationic species. Analysis of characteristic signals with BindFit (1 : 2 binding model) provided association constants (*K*_a_), which suggests that protonation occurs only under sufficiently acidic conditions (SI Table S8).^82^[8]HBI-1 and [8]HBI-2 displayed similar, slightly higher *K*_a_ values than [8]HBI-3, suggesting that [8]HBI-3 is the least basic isomer.

**Fig. 5:**
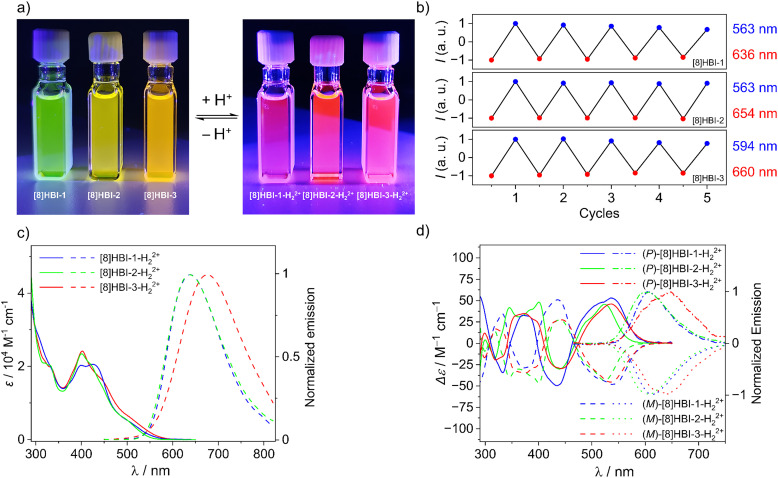
(a) Picture of the toluene solution of neutral and protonated [8]HBIs under 365 nm UV light. (b) Acid/base-switching cycles of the emission between neutral (blue) and dicationic state (red) of [8]HBIs (*c*∼20 µM). Addition of TFA (0.5 M) for the dicationic and Pyridine (0.5 M) for the neutral state. (c) UV–vis and fluorescence spectra and (d) CD and CPL spectra of [8]HBIs in toluene (*c* ∼10^−5^ M) in the presence of TFA (0.5 M).

Protonation induced pronounced optical changes in [8]HBIs in toluene and DCM. Neutral [8]HBI solutions appear bright yellow but turn orange/red upon addition of TFA ([8]HBI-H_2_^2+^), accompanied by fluorescence shifts from green/yellow/orange to deep red (Fig. 5a). UV–Vis absorption and fluorescence titrations revealed continuous bathochromic shifts in absorption edges and emission maxima until saturation, consistent with charge delocalization in the protonated state and narrowing of the optical energy gap to 2.24–2.29 eV. Logarithmic titration plots were linear, consistent with a single-step process. Addition of pyridine regenerated the neutral spectra, and repeat cycling confirmed reversible switching. PLQYs decrease by roughly half upon protonation, excited-state lifetimes were also shortened. The reduced PLQY is attributed to the energy-gap law, as emission shifts into the red–NIR region, increased nonradiative rates diminish efficiency as well as enhanced charge transfer character in protonated state.

Chiroptical properties likewise respond to acid/base stimuli. Consistent with the UV–Vis shifts, the lowest-energy CD bands of the protonated [8]HBIs undergo clear bathochromic shifts relative to the neutral forms, while retaining their sign (Fig. 5d). Importantly, the protonated species maintain large dissymmetry factors with *g*_abs_ values in the range of 2.4 × 10^−2^–4.3 × 10^−2^. Protonated [8]HBIs also display strong CPL in the red–NIR region with *g*_lum_ of 1.4 × 10^−2^–3.1 × 10^−2^, one to two orders of magnitude larger than those of most reported small-molecule acid/base CPL switches (SI Table S1 for comparison).^41,42,70,71,78–81,83^

DFT optimized geometries reveal how protonation alters the helical framework. Notably, all three regioisomers exhibit compression of the helix upon protonation, with [8]HBI-2-H_2_^2+^ showing a more pronounced decrease in pitch (3.25 Å) compared to [8]HBI-1-H_2_^2+^ and [8]HBI-3-H_2_^2+^ (3.39–3.40 Å). Such compression of helical pitch, due to reduced through-space repulsion in protonated state has been recently reported, however, the impact on chiroptical properties was not investigated.^43^ The enhanced *g*_lum_ value for [8]HBI-2-H_2_^2+^ in comparison to the neutral species can be attributed to decreased helical pitch as observed for the bridged [8]HDI based molecular springs.^9^ These regioisomer-dependent conformational responses correlate with the observed electronic and chiroptical differences. These observations highlight how subtle differences in regioisomeric structure and conjugation pathways lead to distinct protonation-induced structural and chiroptical modulations in [8]HBIs, underscoring their potential as responsive chiral materials.

## Conclusion

In summary, the bisbenzimidazole-fused [8]helicenes reported here establish a modular platform in which regioisomerism and external protonation can be used to tune effective π-conjugation and chiroptical response. Crucially, the three regioisomers display distinct balances of through-bond and through-space coupling, which translate into systematic changes in absorption/emission energy, redox splitting, and excited-state dynamics. Protonation provides a reversible chemical handle that compresses the helix in a regioselective manner, red-shifts CD/CPL signatures, and importantly preserves large dissymmetry factors in the switched state. Collectively, these findings reveal how spatial arrangement governs electronic delocalization in nonplanar π-systems and establish a design strategy for next-generation responsive chiral materials.

## Author contributions

A.H. performed the experimental work, analyzed the data, wrote the manuscript, and prepared the supporting information. K.R. and H.B. analyzed the crystallographic data. V.B. performed CPL measurements. P.R. conceived the project, supervised the research, and wrote the manuscript.

## Conflicts of interest

There are no conflicts to declare.

## Supplementary Material

TC-014-D6TC00671J-s001

TC-014-D6TC00671J-s002

## Data Availability

All experimental and computational data supporting this study are included in the manuscript and the supplementary information (SI). Supplementary information is available. See DOI: https://doi.org/10.1039/d6tc00671j. CCDC 2504723 ([8]HBI-1), 2504724 ([8]HBI-2), 2504725 ([8]HBI-3) contain the supplementary crystallographic data for this paper.^84*a*–*c*^

## References

[cit1] Wu Y., Frasconi M., Gardner D. M., McGonigal P. R., Schneebeli S. T., Wasielewski M. R., Stoddart J. F. (2014). Angew. Chem., Int. Ed..

[cit2] Takamuku S., Nakano M., Kertesz M. (2017). Chem. – Eur. J..

[cit3] Li J., Shen P., Zhao Z., Tang Ben Z. (2019). CCS Chem..

[cit4] Zhao X.-J., Hou H., Ding P.-P., Deng Z.-Y., Ju Y.-Y., Liu S.-H., Liu Y.-M., Tang C., Feng L.-B., Tan Y.-Z. (2020). Sci. Adv..

[cit5] Keshri S. K., Ishizuka T., Kojima T., Matsushita Y., Takeuchi M. (2021). J. Am. Chem. Soc..

[cit6] Bansal D., Kundu A., Singh V. P., Pal A. K., Datta A., Dasgupta J., Mukhopadhyay P. (2022). Chem. Sci..

[cit7] Bræstrup C., Xiao X., García-González F., Brock-Nannestad T., Aranda D., Bao S. T., Cavlovic D., Jiang H., Ng F., Nuckolls C., Santoro F., Pittelkow M. (2025). Adv. Opt. Mater..

[cit8] Crassous J. , Helicenes - Synthesis, Properties, and Applications, WILEY-VCH, Weinheim, Germany, 2022

[cit9] Saal F., Zhang F., Holzapfel M., Stolte M., Michail E., Moos M., Schmiedel A., Krause A. M., Lambert C., Wurthner F., Ravat P. (2020). J. Am. Chem. Soc..

[cit10] Saal F., Ravat P. (2021). Synlett.

[cit11] Izquierdo-García P., Fernández-García J. M., Medina Rivero S., Šámal M., Rybáček J., Bednárová L., Ramírez-Barroso S., Ramírez F. J., Rodríguez R., Perles J., García-Fresnadillo D., Crassous J., Casado J., Stará I. G., Martín N. (2023). J. Am. Chem. Soc..

[cit12] Tian X., Shoyama K., Mahlmeister B., Brust F., Stolte M., Würthner F. (2023). J. Am. Chem. Soc..

[cit13] Niu W., Fu Y., Qiu Z.-L., Schürmann C. J., Obermann S., Liu F., Popov A. A., Komber H., Ma J., Feng X. (2023). J. Am. Chem. Soc..

[cit14] Lión-Villar J., Fernández-García J. M., Medina Rivero S., Perles J., Wu S., Aranda D., Wu J., Seki S., Casado J., Martín N. (2025). Nat. Chem..

[cit15] Borstelmann J., Zank S., Krug M., Berger G., Fröhlich N., Glotz G., Gnannt F., Schneider L., Rominger F., Deschler F., Clark T., Gescheidt G., Guldi D. M., Kivala M. (2025). Angew. Chem., Int.
Ed..

[cit16] Zhang Z., Hu W., Liu Z., Tsutsui Y., Murata Y., Seki S., Hirose T. (2025). J. Am. Chem. Soc..

[cit17] Ousaka N., Yashima E. (2021). Chem. Lett..

[cit18] Saal F., Swain A., Schmiedel A., Holzapfel M., Lambert C., Ravat P. (2023). Chem. Commun..

[cit19] Saal F., Brancaccio V., Radacki K., Braunschweig H., Ravat P. (2025). Angew. Chem., Int. Ed..

[cit20] Zhang F., Radacki K., Braunschweig H., Lambert C., Ravat P. (2021). Angew. Chem., Int. Ed..

[cit21] Shen Y., Chen C.-F. (2012). Chem. Rev..

[cit22] Gingras M. (2013). Chem. Soc. Rev..

[cit23] Ravat P. (2021). Chem. – Eur. J..

[cit24] Nakai Y., Mori T., Inoue Y. (2012). J. Phys. Chem. A.

[cit25] Zhao W. L., Li M., Lu H. Y., Chen C. F. (2019). Chem. Commun..

[cit26] Kubo H., Hirose T., Nakashima T., Kawai T., Hasegawa J.-Y., Matsuda K. (2021). J. Phys. Chem. Lett..

[cit27] Zhang F., Rauch F., Swain A., Marder T. B., Ravat P. (2023). Angew. Chem., Int. Ed..

[cit28] Yu Y., Wang C., Hung F.-F., Jiang L., Che C.-M., Liu J. (2025). Angew. Chem., Int. Ed..

[cit29] Kumar V., Páez J. L., Míguez-Lago S., Cuerva J. M., Cruz C. M., Campaña A. G. (2025). Chem. Soc. Rev..

[cit30] Brandt J. R., Salerno F., Fuchter M. J. (2017). Nat. Rev. Chem..

[cit31] Furlan F., Moreno-Naranjo J. M., Gasparini N., Feldmann S., Wade J., Fuchter M. J. (2024). Nat. Photon..

[cit32] VanOrman Z. A., Kitzmann W. R., Reponen A.-P. M., Deshpande T., Jöbsis H. J., Feldmann S. (2025). Nat. Rev. Chem..

[cit33] Kiran V., Mathew S. P., Cohen S. R., Hernandez Delgado I., Lacour J., Naaman R. (2016). Adv. Mater..

[cit34] Kettner M., Maslyuk V. V., Nurenberg D., Seibel J., Gutierrez R., Cuniberti G., Ernst K. H., Zacharias H. (2018). J. Phys. Chem. Lett..

[cit35] Rodriguez R., Naranjo C., Kumar A., Matozzo P., Das T. K., Zhu Q., Vanthuyne N., Gomez R., Naaman R., Sanchez L., Crassous J. (2022). J. Am. Chem. Soc..

[cit36] Giaconi N., Poggini L., Lupi M., Briganti M., Kumar A., Das T. K., Sorrentino A. L., Viglianisi C., Menichetti S., Naaman R. (2023). ACS Nano.

[cit37] Bloom B. P., Paltiel Y., Naaman R., Waldeck D. H. (2024). Chem. Rev..

[cit38] Yang Y., da Costa R. C., Fuchter M. J., Campbell A. J. (2013). Nat. Photonics.

[cit39] Li J.-K., Zhang M.-Y., Zeng L., Huang L., Wang X.-Y. (2023). Angew. Chem., Int. Ed..

[cit40] Nakakuki Y., Hirose T., Matsuda K. (2018). J. Am. Chem. Soc..

[cit41] Sakai H., Kubota T., Yuasa J., Araki Y., Sakanoue T., Takenobu T., Wada T., Kawai T., Hasobe T. (2016). Org. Biomol. Chem..

[cit42] Pascal S., Besnard C., Zinna F., Di Bari L., Le Guennic B., Jacquemin D., Lacour J. (2016). Org. Biomol. Chem..

[cit43] Karak P., Choudhury J. (2022). Chem. Sci..

[cit44] Li C., Zhang C., Li P., Jia Y., Duan J., Liu M., Zhang N., Chen P. (2023). Angew. Chem., Int. Ed..

[cit45] Ray C., Díaz-Norambuena C., Johnson M., Moreno F., Maroto B. L., Bañuelos J., Muller G., de la Moya S. (2023). J. Mater. Chem. C.

[cit46] Agrawal A. R., Shioukhi I., Deree Y., Bogoslavsky B., Shalev O., Hoffman R., Gidron O. (2025). Angew. Chem., Int. Ed..

[cit47] Isla H., Crassous J. (2016). C. R. Chim.

[cit48] Ravat P., Šolomek T., Juríček M. (2019). ChemPhotoChem.

[cit49] Ravat P., Šolomek T., Häussinger D., Blacque O., Juríček M. (2018). J. Am. Chem. Soc..

[cit50] Isla H., Srebro-Hooper M., Jean M., Vanthuyne N., Roisnel T., Lunkley J. L., Muller G., Williams J. A. G., Autschbach J., Crassous J. (2016). Chem. Commun..

[cit51] Yen-Pon E., Buttard F., Frédéric L., Thuéry P., Taran F., Pieters G., Champagne P. A., Audisio D. (2021). JACS Au.

[cit52] Günther K., Grabicki N., Battistella B., Grubert L., Dumele O. (2022). J. Am. Chem. Soc..

[cit53] Guy L., Mosser M., Pitrat D., Mulatier J.-C., Kukułka M., Srebro-Hooper M., Jeanneau E., Bensalah-Ledoux A., Baguenard B., Guy S. (2023). Molecules.

[cit54] Swain A., Radacki K., Braunschweig H., Ravat P. (2024). Chem. Sci..

[cit55] Dongre S. D., Venugopal G., Kumar V., Badrinarayan Jadhav A., Kumar J., Santhosh Babu S. (2025). Angew. Chem., Int. Ed..

[cit56] Loutfy R. O., Hor A. M., Kazmaier P. M., Burt R. A., Hamer G. K. (1991). Dyes Pigm..

[cit57] Nagao Y. (1997). Prog. Org. Coat..

[cit58] Quante H., Geerts Y., Müllen K. (1997). Chem. Mater..

[cit59] Mizuguchi J., Shimo N. (2006). J. Imaging Sci. Tech.

[cit60] Fortage J., Séverac M., Houarner-Rassin C., Pellegrin Y., Blart E., Odobel F. (2008). J. Photochem. Photobiol., A.

[cit61] Ortiz R. P., Herrera H., Blanco R., Huang H., Facchetti A., Marks T. J., Zheng Y., Segura J. L. (2010). J. Am. Chem. Soc..

[cit62] Mamada M., Pérez-Bolívar C., Kumaki D., Esipenko N. A., Tokito S., Anzenbacher Jr P. (2014). Chem. – Eur. J..

[cit63] Behera P. K., Yadav K., Patra A., Gupta R. K., Rao D. S. S., Kumar S., Pandey U. K., Achalkumar A. S. (2023). Chem. – Eur. J..

[cit64] Yuan Z., Xiao Y., Li Z., Qian X. (2009). Org. Lett..

[cit65] Mamada M., Pérez-Bolívar C., Anzenbacher, Jr. P. (2011). Org. Lett..

[cit66] Taublaender M. J., Glocklhofer F., Marchetti-Deschmann M., Unterlass M. M. (2018). Angew. Chem., Int. Ed..

[cit67] Mizuguchi J. (2004). J. Phys. Chem. B.

[cit68] Schönamsgruber J., Maid H., Bauer W., Hirsch A. (2014). Chem. – Eur. J..

[cit69] Schönamsgruber J., Hirsch A. (2015). Eur. J. Org. Chem..

[cit70] Saleh N., Moore II B., Srebro M., Vanthuyne N., Toupet L., Williams J. A. G., Roussel C., Deol K. K., Muller G., Autschbach J., Crassous J. (2015). Chem. – Eur. J..

[cit71] García-Cerezo P., Codesal M. D., David A. H. G., Le Bras L., Abid S., Li X., Miguel D., Kazem-Rostami M., Champagne B., Campaña A. G., Stoddart J. F., Blanco V. (2025). Adv. Mater..

[cit72] Tanaka H., Inoue Y., Mori T. (2018). ChemPhotoChem.

[cit73] Cei M., Di Bari L., Zinna F. (2023). Chirality.

[cit74] Longhi G., Castiglioni E., Koshoubu J., Mazzeo G., Abbate S. (2016). Chirality.

[cit75] Liu Y., Cerezo J., Mazzeo G., Lin N., Zhao X., Longhi G., Abbate S., Santoro F. (2016). J. Chem. Theory Comput..

[cit76] Zhou Z., Fu L., Hu Y., Wang X. Y., Wei Z., Narita A., Mullen K., Petrukhina M. A. (2020). Angew. Chem., Int. Ed..

[cit77] Heckmann A., Lambert C. (2012). Angew. Chem., Int. Ed..

[cit78] Inoue R., Aoki A., Agou T., Morisaki Y. (2025). Angew. Chem., Int. Ed..

[cit79] Miwa S., Mizutani D., Kawano K., Matsuzaki K., Nagata Y., Tsubaki K., Takasu K., Takikawa H. (2025). Chem. – Eur. J..

[cit80] Hanada K., Nogami J., Miyamoto K., Hayase N., Nagashima Y., Tanaka Y., Muranaka A., Uchiyama M., Tanaka K. (2021). Chem. – Eur. J..

[cit81] Maeda C., Daigen Y., Michishita S., Ema T. (2025). Org. Lett..

[cit82] Thordarson P. (2011). Chem. Soc. Rev..

[cit83] Otani T., Tsuyuki A., Iwachi T., Someya S., Tateno K., Kawai H., Saito T., Kanyiva K. S., Shibata T. (2017). Angew. Chem., Int. Ed..

[cit84] (a) CCD C 2504723: Experimental Crystal Structure Determination, 2026 10.5517/ccdc.csd.cc2q2cjq

