# Supplementary material for: Through-bond versus through-space conjugation and high-dissymmetry chiroptical switching in proton–responsive [8]helicene bisbenzimidazoles
Source: J Mater Chem C Mater. 2026 Mar 20;14(19):8099–107. doi: 10.1039/d6tc00671j (PMC13022832; doi:10.1039/d6tc00671j)
Supplement: TC-014-D6TC00671J-s001 [file TC-014-D6TC00671J-s001.pdf]

Supplementary Information (SI) for Journal of Materials Chemistry C.
This journal is © The Royal Society of Chemistry 2026

Supplementary Information for

Through-Bond versus Through-Space Conjugation and High Dissymmetry Chiroptical Switching in Proton–Responsive [8]Helicene Bisbenzimidazoles

Amira A. C. Hartmann,‡ Vincenzo Brancaccio,# Krzysztof Radacki,† Holger Braunschweig,† and Prince Ravat‡*

‡Department of Chemistry and Biochemistry, Institute of Organic Chemistry, 50939 Cologne, Germany

#Institut für Organische Chemie, Julius-Maximilians-Universität Würzburg, Am Hubland, D-97074 Würzburg, Germany

†Institut für Anorganische Chemie and Institute for Sustainable Chemistry & Catalysis with Boron, Julius-Maximilians-Universität Würzburg, Am Hubland, D-97074 Würzburg, Germany

*Email: pravat@uni-koeln.de

Table of contents

S1. Experimental details **2**

S2. Synthesis **4**

S3. Photophysical properties **12**

S4. Time-resolved fluorescence decay **17**

S6. Cyclic voltammetry **23**

S7. Quantum chemical calculations **24**

S8. Single crystal data **33**

S9. NMR spectra **36**

S10. High-resolution mass spectrometry **49**

S11. Cartesian coordinates **50**

S12. References **50**

S1. Experimental details

Synthesis and Materials

All chemicals and solvents were purchased from commercial sources and were used without further purification unless stated otherwise. Racemic and enantiopure [8]helicene-4,5,16,17-tetracarboxylic dianhydride was synthesized according to literature known protocols.[1] The reactions and experiments sensitive to dioxygen were performed using Schlenk techniques and nitrogen-saturated solvents.

Chromatography

Open-column chromatography and thin-layer chromatography (TLC) were performed on silica gel (Merck silica gel 60, 40–63 µm).

NMR Spectroscopy

The NMR experiments were performed at 298 K on NMR spectrometers operating at 400 MHz or 600 MHz ^{1}H and 101 MHz or 151 MHz ^{13}C frequencies. Standard pulse sequences were used, and the data was processed using 2-fold zero-filling in the indirect dimension for all 2D experiments. Chemical shifts (δ) are reported in parts per million (ppm) relative to the solvent residual peak (^{1}H and ^{13}C NMR, respectively): CD_2Cl_2 (δ = 5.32 and 54.00 ppm) and J values are given in Hz. Structural assignment was made with additional information from *g*COSY, *g*NOESY, HSQC and HMBC experiments.

HRMS

MALDI-TOF-HRMS were measured on a Bruker ultrafleXtreme mass spectrometer. *Trans*-2-[3-(4-*tert*-butylphenyl)-2-methyl-2-propenylidene]malononitrile (DCTB) dissolved in chloroform (30 mg/mL^{-1}) was used as supporting matrix in the MALDI–TOF–HRMS measurement. Reference spectra were simulated using the mMass software.

UV–vis Absorption Spectroscopy

UV–vis spectra were measured on a JASCO V–670 spectrophotometer.

Emission Spectroscopy

Room temperature florescence emission spectra were measured on an Edinburgh FLS 980 photoluminescence spectrometer. A rectangular 10 mm quartz glass cuvette with a Teflon screw cap was used for the measurements.

Fluorescence Lifetime and Quantum Yield

The fluorescence lifetimes were measured on an Edinburgh FLS 980 photoluminescence spectrometer using the Time Correlated Single Photon Counting (TCSPC) technique for the acquisition of single photons. The samples were dissolved in the respective solvent and a 418.6 nm pulsed laser diode with a pulse frequency of 10,000,000 Hz (pulse-to-pulse time 100 ns) or 5,000,000 Hz (pulse-to-pulse time 200 ns) was used for excitation. Decays were recorded to 10,000 counts in the peak channel with a record length of 8192 channels. The band pass of the monochromator was adjusted to give a signal count rate of <100 kHz. The instrument response function (IRF) was recorded by measuring the excitation signal using a cuvette filled with LUDOX® colloidal silica suspension to scatter the laser light. The resulting spectra were analyzed with the FAST (Fluorescence Analysis Software Technology) software by Edinburgh Instruments, where the fluorescence lifetimes were calculated by mono- or biexponential iterative reconvolution fits employing non-linear least-squares analysis, depending on the compound and solvent. The quality of the fits was judged by the calculated value of the reduced $\chi2$ and visual inspection of the weighted residuals.

The absolute fluorescence quantum yields were measured on the same spectrometer with an Integrating Sphere Assembly calibrated integrating sphere (F-M01) installed. A 450 W xenon arc lamp was used as a light source. The raw quantum yields were calculated within the F980 software by Edinburgh Instruments. Due to the low extinction coefficients of the compounds in the overlap of absorption and emission spectra, a self-absorption correction was not performed.

Electronic Circular Dichroism and Circularly Polarized Luminescence Spectroscopy

Electronic Circular Dichroism (ECD) spectra were recorded on either a Jasco J-810 CD spectropolarimeter, or a customized JASCO CPL-300/J–1500 hybrid spectrometer, at 20.0 °C.

Circularly Polarized Luminescence (CPL) spectra were recorded with a customized JASCO CPL-300/J–1500 hybrid spectrometer at 20.0 °C. Excitation/Emission band widths for the

neutral Compounds 20 nm / 10 nm and for the compounds after TFA addition 28 / 14 nm were used for the CPL measurement.

Cyclic Voltammetry and Differential Pulse Voltammetry

Cyclic voltammetry and differential pulse voltammetry experiments were performed in DCM with 0.2 M $[Bu_4N][PF_6]$ as supporting electrolyte, using a Gamry Instruments Reference 600 potentiostat. A standard three-electrode cell configuration was employed, using a platinum disk working electrode, a platinum wire counter electrode, and a platinum wire serving as reference electrode. The redox potentials were referenced to the ferrocene (Fc) / ferrocenium (Fc^+) redox couple.

S2. Synthesis

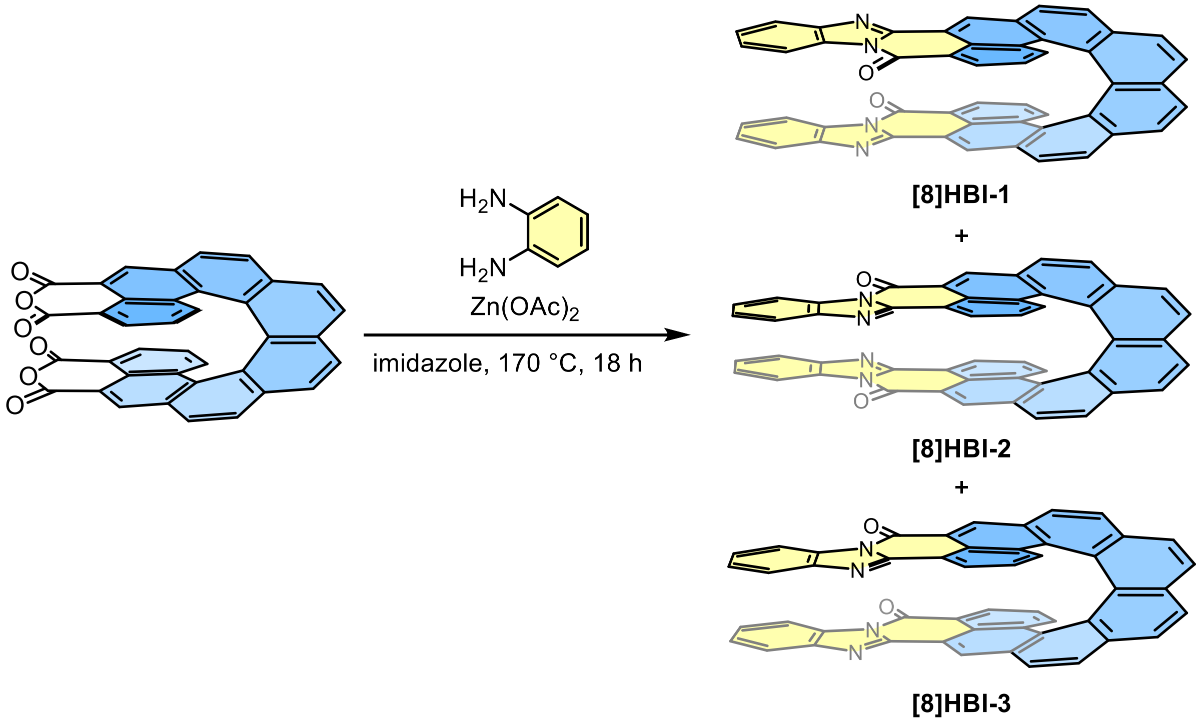

[8]Helicene dianhydride (20.0 mg, 35.2 µmol, 1.0 eq.), 1,2-diaminobenzene (12.7 mg, 117 µmol, 3.3 eq.), zinc acetate (6.45 mg, 35.2 µmol, 1.0 eq.) and imidazole (2.0 g) were heated at 170 °C for 18 h under an inert atmosphere. The cooled reaction mixture was suspended in 10% HCl (20 mL). The precipitates were washed with 10% HCl (50 mL) and dried under reduced pressure. The residue was purified by column chromatography on silica gel (DCM/MeOH 99.8/0.2 to DCM/MeOH 99.2/0.8). The solvent was removed, and the three regioisomers were collected. This synthesis was performed with racemic and enantiopure starting materials, resulting in racemic and enantiopure products, respectively.

Yield: [8]HBI-1: 2.5 mg; [8]HBI-2: 10 mg; [8]HBI-3: 12 mg (Σ=24.5mg, 34.7 μmol, 98%) of yellow to orange powders.

[8]HBI-1:

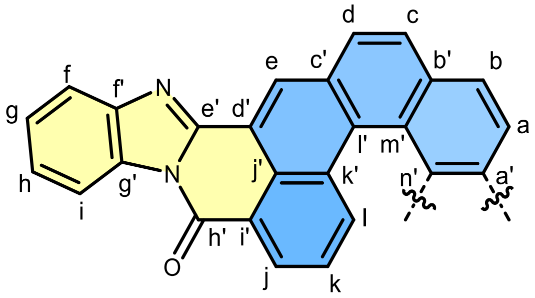

^{1}H NMR (400.1 MHz, CD_2Cl_2, rt): δ [ppm] = 8.43 (s, $2H_e$), 8.33 (dd, ^3J = 7.3 Hz, ^4J = 1.2 Hz, $2H_j$), 8.17 (d, ^3J = 8.3 Hz, $2H_a$), 8.09 (d, ^3J = 8.3 Hz, $2H_b$), 7.98 (d, ^3J = 8.3 Hz, $2H_c$), 7.76 (ddd, ^3J = 8.0 Hz, ^4J = 1.2 Hz, ^5J = 0.6 Hz, $2H_f$), 7.64 (d, ^3J = 8.3 Hz, $2H_d$), 7.61 (dd, ^3J = 8.3 Hz, ^4J = 1.1 Hz, $2H_l$), 7.61 (ddd, ^3J = 8.0 Hz, ^4J = 1.2 Hz, ^5J = 0.6 Hz, $2H_i$), 7.17 (ddd, ^3J = 8.0 Hz, ^3J = 7.3 Hz, ^4J = 1.3 Hz, $2H_h$), 6.96 (ddd, ^3J = 8.0 Hz, ^3J = 7.3 Hz, ^4J = 1.3 Hz, $2H_g$), 6.84 (dd, ^3J = 8.3 Hz, ^3J = 7.3 Hz, $2H_k$).

^{13}C NMR (150.9 MHz, CD_2Cl_2, rt): δ [ppm] = 160.41 ($C_{h'}$=O), 148.07 ($C_{e'}$=N), 143.63 ($C_{q,f'}$), 134.35 (C_q), 133.33 (C_q), 132.31 (C_q), 131.32 (C_q), 131.19 (C_q), 130.75 (C_lH), 129.52 (C_q), 129.41 (C_jH), 128.93 (C_cH), 128.81 (C_aH), 127.98 (C_bH), 127.78 (C_dH), 127.62 (C_eH), 127.16 (C_q), 125.82 (C_q), 125.79 (C_gH), 125.75 (C_hH), 125.39 (C_kH), 124.61 (C_q), 122.32 (C_q), 119.70 (C_fH), 118.18 (C_q), 115.56 (C_iH).

HRMS (MALDI): m/z: calculated $[M]^-$ 712.19048; found $[M]^-$ 712.19554 (|Δ| = 7.1 ppm).

[8]HBI-2:

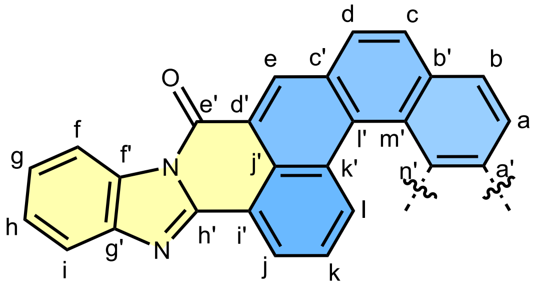

^{1}H NMR (400.1 MHz, CD_2Cl_2, rt): δ [ppm] = 8.34 (dd, ^3J = 7.3 Hz, ^4J = 1.1 Hz, $2H_j$), 8.32 (s, $2H_e$), 8.19 (d, ^3J = 8.3 Hz, $2H_a$), 8.09 (d, ^3J = 8.3 Hz, $2H_b$), 7.98 (d, ^3J = 8.3 Hz, $2H_c$), 7.86 (ddd, ^3J = 8.0 Hz, ^4J = 1.2 Hz, ^5J = 0.6 Hz, $2H_i$), 7.64 (d, ^3J = 8.3 Hz, $2H_d$), 7.45 (ddd, ^3J = 8.0 Hz, ^3J = 7.3 Hz, ^4J = 1.3 Hz, $2H_g$), 7.36 (dd, ^3J = 8.3 Hz, ^4J = 1.1 Hz, $2H_l$), 7.24 (ddd, ^3J = 8.0 Hz,

^{4}J = 1.2 Hz, ^{5}J = 0.6 Hz, 2H$_{f}$), 7.08 (ddd, ^{3}J = 8.0 Hz, ^{3}J = 7.3 Hz, ^{4}J = 1.3 Hz, 2H$_{h}$), 6.78 (dd, ^{3}J = 8.3 Hz, ^{3}J = 7.3 Hz, 2H$_{k}$).

^{13}C NMR (100.6 MHz, CD_2Cl_2, rt): δ [ppm] = 159.26 (C$_{e'}$=O), 149.56 (C$_{h'}$=N), 144.23 (C$_{q,f'}$), 134.89 (C$_{q,b'}$), 133.54 (C$_{e}$H), 132.20 (C$_{q,a'}$), 131.67 (C$_{q,g'}$), 131.29 (C$_{q,l'}$), 130.51 (C$_{q,c'}$), 129.38 (C$_{a}$H), 128.89 (C$_{c}$H), 128.28 (C$_{d}$H), 128.00 (C$_{l}$H), 127.97 (C$_{b}$H), 126.98 (C$_{q,k'}$), 126.80 (C$_{q,n'}$), 125.74 (C$_{k}$H), 125.71 (C$_{h}$H), 125.70 (C$_{g}$H), 125.00 (C$_{q,j'}$), 124.84 (C$_{j}$H), 124.47 (C$_{q,m'}$), 120.48 (C$_{q,d'}$), 120.08 (C$_{q,i'}$), 119.75 (C$_{i}$H), 115.92 (C$_{f}$H).

HRMS (MALDI): m/z: calculated $[M]^-$ 712.19048; found $[M]^-$ 712.19450 ($|\Delta|$ = 5.6 ppm).

[8]HBI-3:

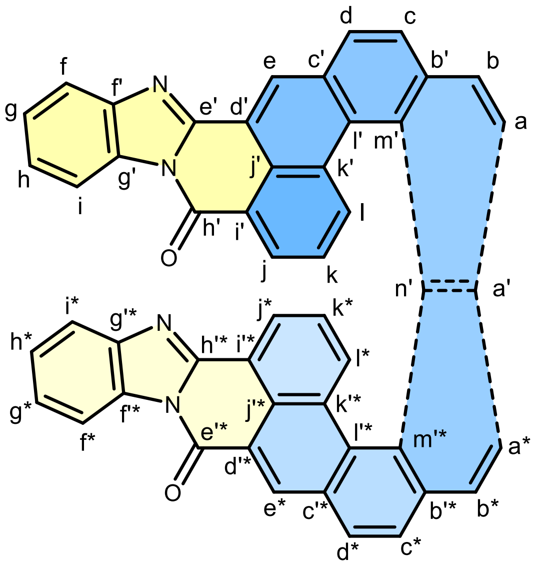

^{1}H NMR (400.1 MHz, CD_2Cl_2, rt): δ [ppm] = 8.40 (ddd, ^{3}J = 8.0 Hz, ^{4}J = 1.2 Hz, ^{5}J = 0.6 Hz, 1H$_{f*}$), 8.37 (dd, ^{3}J = 7.3 Hz, ^{4}J = 1.1 Hz, 1H$_{j*}$), 8.37 (s, 1H$_{e}$), 8.35 (dd, ^{3}J = 7.3 Hz, ^{4}J = 1.1 Hz, 1H$_{j}$), 8.32 (s, 1H$_{e*}$), 8.19 (d, ^{3}J = 8.3 Hz, 1H$_{a*}$), 8.16 (d, ^{3}J = 8.3 Hz, 1H$_{a}$), 8.08 (d, ^{3}J = 8.3 Hz, 1H$_{b}$), 8.07 (d, ^{3}J = 8.3 Hz, 1H$_{b*}$), 7.95 (d, ^{3}J = 8.3 Hz, 1H$_{c*}$), 7.94 (d, ^{3}J = 8.3 Hz, 1H$_{c}$), 7.69 (ddd, ^{3}J = 8.0 Hz, ^{4}J = 1.2 Hz, ^{5}J = 0.6 Hz, 1H$_{f}$), 7.61 (d, ^{3}J = 8.3 Hz, 1H$_{d*}$), 7.60 (d, ^{3}J = 8.3 Hz, 1H$_{d}$), 7.54 (dd, ^{3}J = 8.3 Hz, ^{4}J = 1.1 Hz, 1H$_{l}$), 7.48 (ddd, ^{3}J = 8.0 Hz, ^{4}J = 1.2 Hz, ^{5}J = 0.6 Hz, 1H$_{i}$), 7.37 (dd, ^{3}J = 8.3 Hz, ^{4}J = 1.1 Hz, 1H$_{l*}$), 7.30 (ddd, ^{3}J = 8.0 Hz, ^{3}J = 7.3 Hz, ^{4}J = 1.3 Hz, 1H$_{h*}$), 7.24 (ddd, ^{3}J = 8.0 Hz, ^{3}J = 7.3 Hz, ^{4}J = 1.3 Hz, 1H$_{g}$), 7.00 (ddd, ^{3}J = 8.0 Hz, ^{3}J = 7.3 Hz, ^{4}J = 1.3 Hz, 1H$_{g*}$), 6.89 (ddd, ^{3}J = 8.0 Hz, ^{3}J = 7.3 Hz, ^{4}J = 1.3 Hz, 1H$_{h}$), 6.88 (ddd, ^{3}J = 8.0 Hz, ^{4}J = 1.2 Hz, ^{5}J = 0.6 Hz, 1H$_{i*}$), 6.84 (dd, ^{3}J = 8.3 Hz, ^{3}J = 7.3 Hz, 1H$_{k}$). 6.81 (dd, ^{3}J = 8.3 Hz, ^{3}J = 7.3 Hz, 1H$_{k*}$).

^{13}C NMR (150.9 MHz, CD_2Cl_2, rt): δ [ppm] = 160.64 (C$_{h'}$=O), 159.67 (C$_{e'*}$=O), 149.43 (C$_{h'*}$=N), 148.19 (C$_{e'}$=N), 143.95 (C$_{q,g'*}$), 143.56 (C$_{q,f'}$), 134.81 (C$_{q}$), 134.20 (C$_{q}$),

133.10 (C_{e*}H), 132.07 (C_q), 131.56 (C_q), 131.46 (C_q), 131.33 (C_q), 131.09 (C_lH), 131.05 (C_q), 130.22 (C_q), 129.75 (C_q), 129.69 (C_q), 129.36 (C_jH), 129.30 (C_{a*}H), 128.68 (C_aH), 128.64 (C_cH), 128.08 (C_{c*}H), 127.90 (C_dH), 127.89 (C_{d*}H), 127.69 (C_bH), 127.52 (C_{b*}H), 127.49 (C_{l*}H), 126.99 (C_q), 126.84 (C_q), 126.70 (C_q), 126.33 (C_eH), 126.01 (C_{g*}H), 125.90 (C_{k*}H), 125.58 (C_hH), 125.56 (C_kH), 125.42 (C_gH), 125.21 (C_{h*}H), 125.05 (C_q), 124.95 (C_{j*}H), 124.58 (C_q), 124.33 (C_q), 122.35 (C_q), 120.60 (C_q), 120.04 (C_q), 119.46 (C_{i*}H), 119.36 (C_fH), 117.98 (C_q), 115.54 (C_{f*}H), 115.09 (C_iH).

HRMS (MALDI): m/z: calculated $[M]^-$ 712.19048; found $[M]^-$ 712.19493 ($|\Delta|$ = 6.2 ppm).

The synthesis is preferentially forming isomers **[8]HBI-2** and **[8]HBI-3**, while **[8]HBI-1** is obtained significantly smaller ratio. This selectivity arises from steric hindrance that occurs after imide formation. The reaction proceeds through two possible imidazole formations, involving an attack on either the inside or outside carbonyl group. The overall reaction consists of two consecutive steps.

The steric hindrance can be characterized using the buried volume method, with the standardized radius of 3.5 Å (for the buried volume method) for the carbon atom involved in the nucleophilic attack. The buried volume is the percentual fraction of the total volume of the sphere around the selected group that is sterically occupied. The molecule structures were optimized using ωB97XD functional and 6-31G(d,p) basis set in the gas phase using Gaussian 16 suite.[2] In the initial imidazole formation, the internal attack is less sterically hindered. In the second step, **[8]HBI-2** is favored over **[8]HBI-3**, while the alternative second step favors **[8]HBI-3** over **[8]HBI-1**.[3-5]

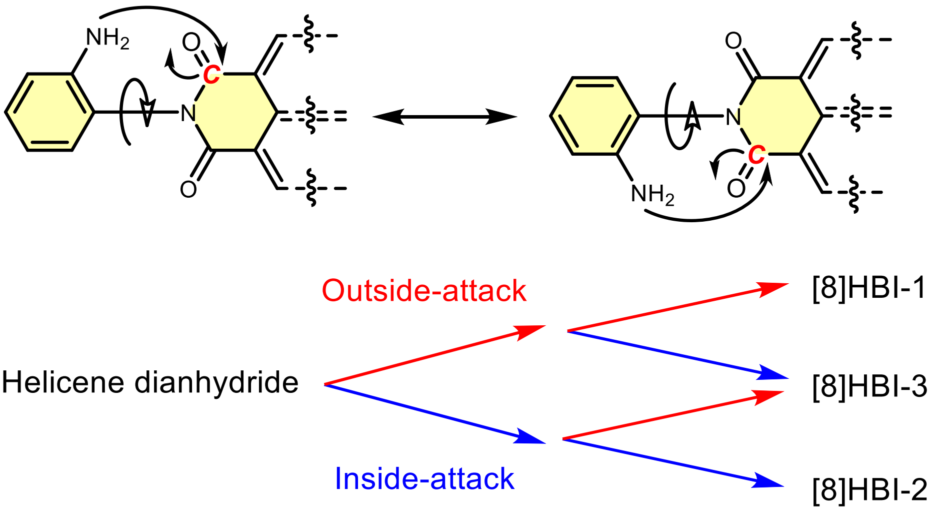

Figure S1. Visualization of the two possible nucleophilic attacks at the carbonyl carbon and the resulting two step formation of the three isomers.

Molecular graphics and analyses performed with UCSF ChimeraX, developed by the Resource for Biocomputing, Visualization, and Informatics at the University of California, San Francisco, with support from National Institutes of Health R01-GM129325 and the Office of Cyber Infrastructure and Computational Biology, National Institute of Allergy and Infectious Diseases.[4]

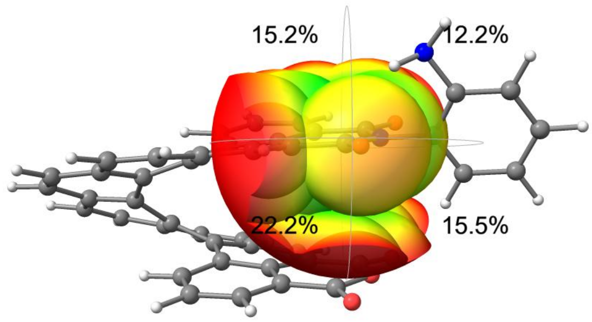

Outside-attack

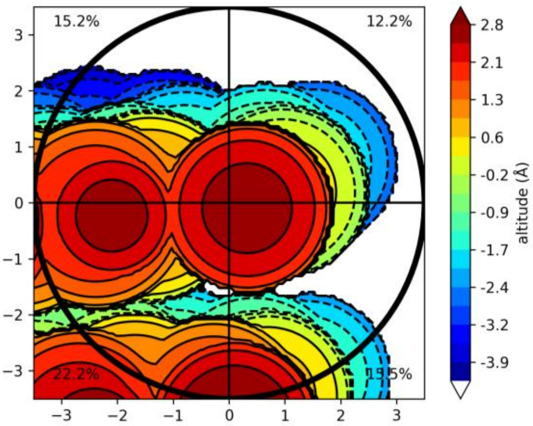

V_{bur} = 65.1 %

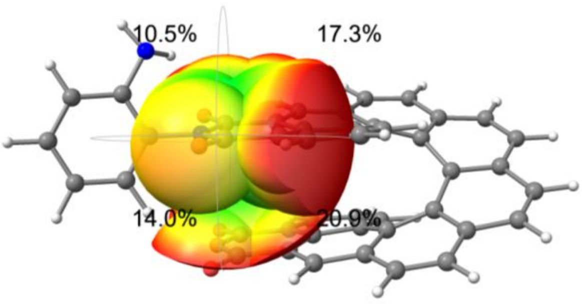

Inside-attack

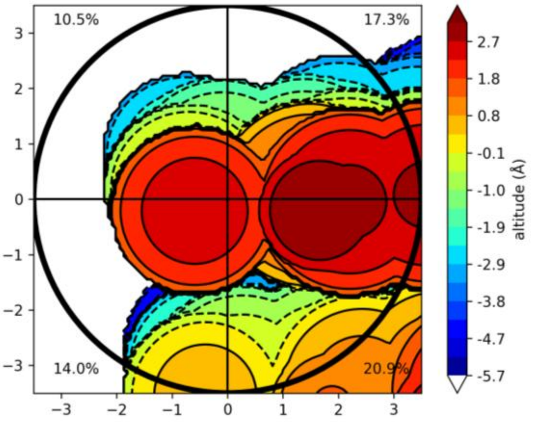

V_{bur} = 62.7 %

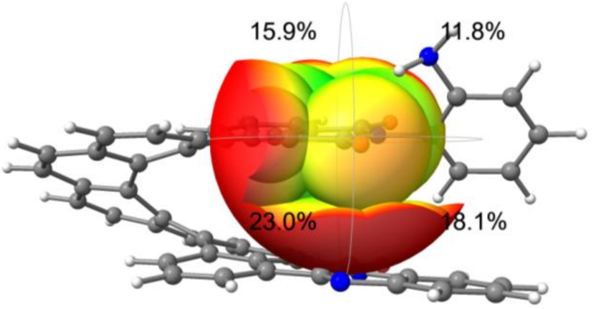

HBI-3 (Outside-attack)

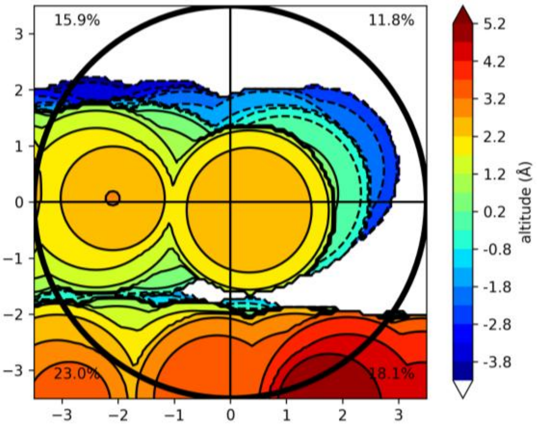

V_{bur} = 68.8 %

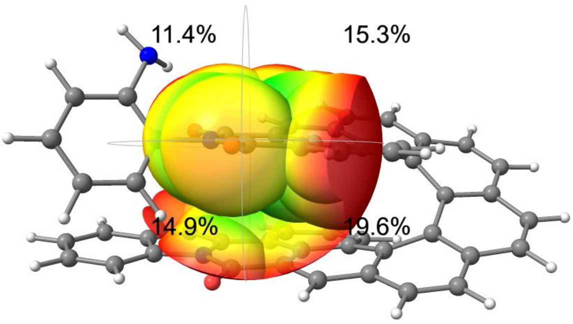

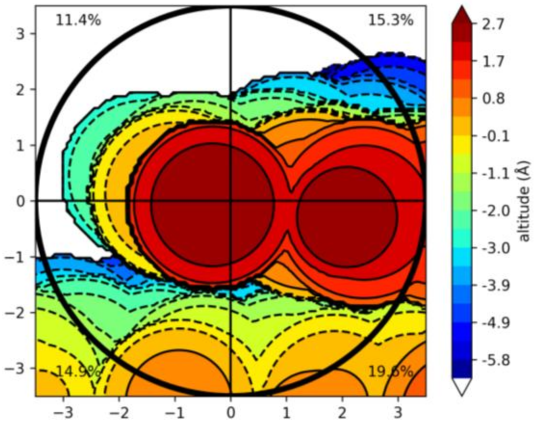

HBI-2

V_{bur} = 61.2 %

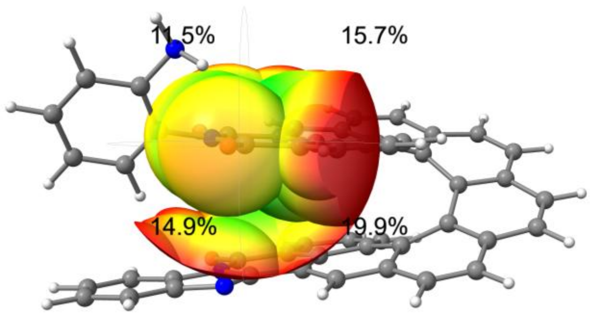

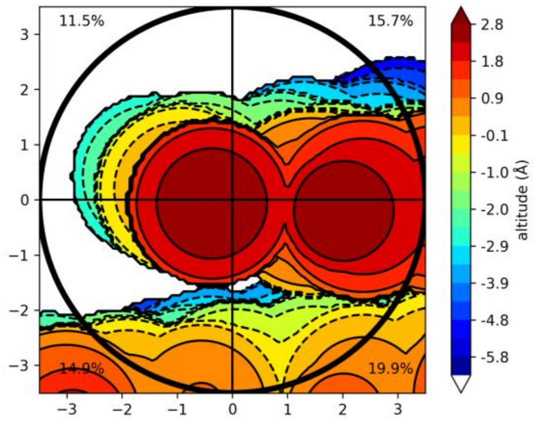

HBI-3 (Inside-attack)

V_{bur} = 62.0 %

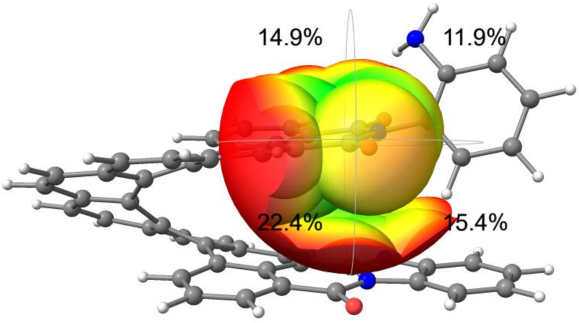

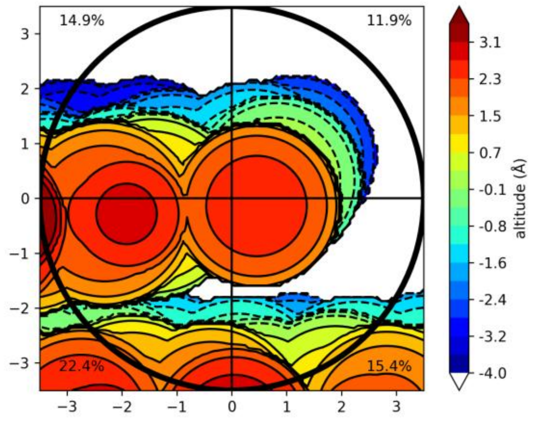

HBI-1

V_{bur} = 64.6 %

Figure S2. Visualization of the buried volume method. On the left side is the molecule with the 3.5 Å radius sphere around the attacked carbonyl carbon and on the right side is the corresponding steric maps.

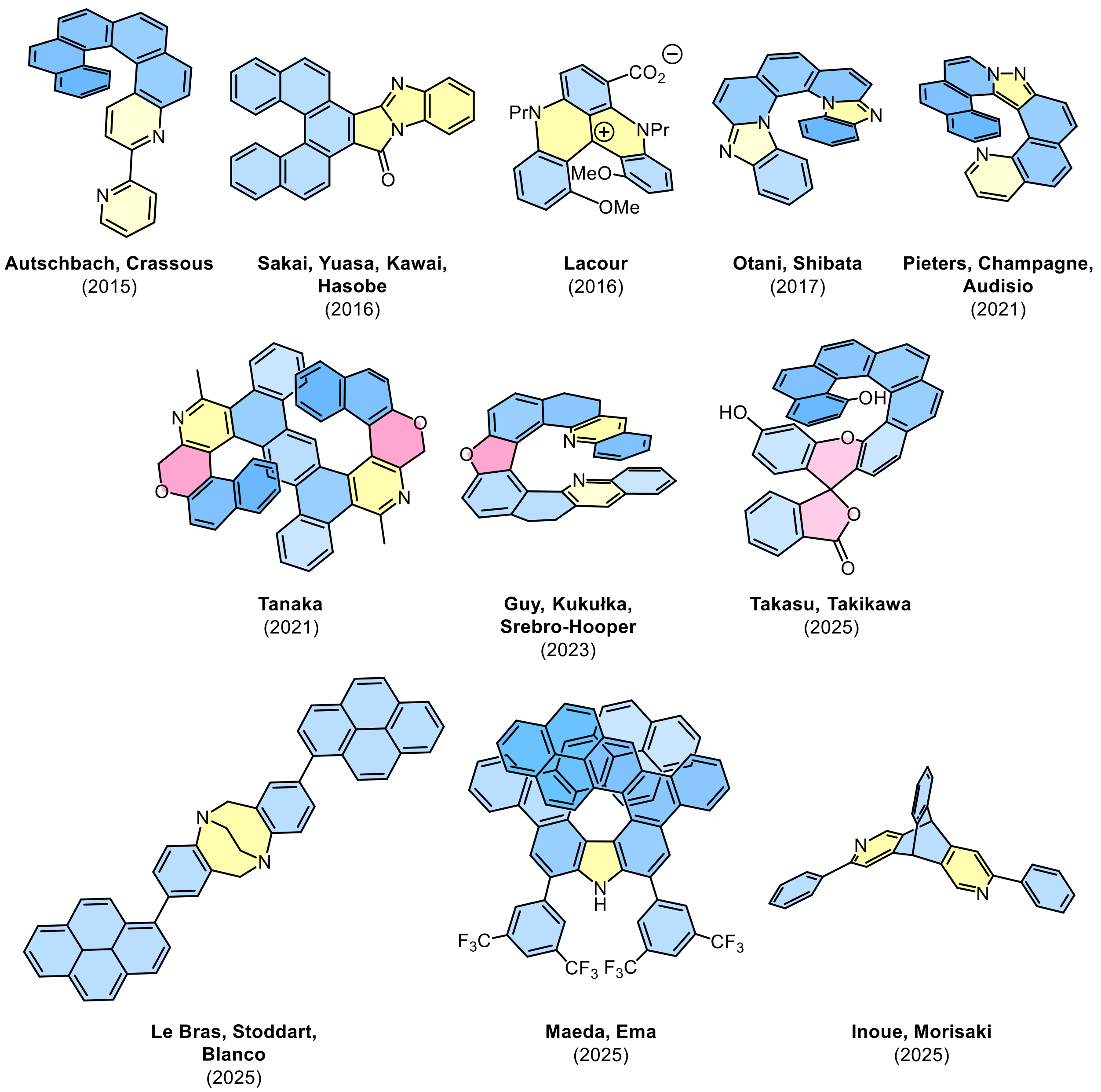

Figure S3. Selected literature examples of acid/base-triggered chiroptical switches.[6-16]

Table S1. Overview of chiroptical properties of the compounds shown in **Figure S3.**[6-16]

	State	g_{abs} / 10^{-3}	g_{lum} / 10^{-3}	λ_{em} / nm	Φ_{FL}	Solvent	Acid/Base
**Autschbach, Crassous** (2015)	neutral	-	3.2 (average)	421, 445	0.084	DCM	Na_2CO_3
	protonated	-	2.9 (average)	590	0.082		$[H_2O{\cdot}HBF_4]_2$ [18C6]
**Sakai, Yuasa, Kawai, Hasobe** (2016)	neutral	-	9.45	575 (approx.)	0.06 (approx.)	DCM	Pyridine
	protonated	-	5.92	630 (approx.)	0.06		TFA
**Lacour** (2016)	neutral	-	-	709	0.01	CH_3CN	NaOH
	protonated	0.4	0.5	654	0.29		HCl
**Otani, Shibata** (2017)	neutral	-	9.0	473	0.39	DCM	-
	protonated	-	8.0	514	0.80		TFA
**Pieters, Champagne, Audisio** (2021)	neutral	-	1.1	436	0.17	DCM	DBU
	protonated	-	1.2	585	0.1		TFA
**Tanaka** (2021)	neutral	5.38	1.42	489	0.21	$CHCl_3$	-
	protonated	4.98	1.20	555	0.32		TFA
**Guy, Kukułka, Srebro-Hooper** (2023)	neutral	-	14	440	0.05	DCM	NaOH
	protonated	-	14	560	0.05		TFA
**Takasu, Takikawa** (2025)	neutral	-	1.58	439, 463	0.014	DCM	TFA
	deprotonated	-	4.61	636	0.031		DBU
**Le Bras, Stoddart, Blanco** (2025)	neutral	0.3	0.53	431	0.61	DCM	Et_3N
	protonated	0.4	0.3	397	0.45		TFA
**Maeda, Ema** (2025)	neutral	5.1	4.2	480	0.39	DCM	MSA
	deprotonated	-	2.3	590 (approx.)	-		TBAF/TBD
**Inoue, Morisaki** (2025)	neutral	0.16	0.0	416	< 0.01	CH_3CN	TEA
	mono-protonated	1.2	2.3	510	0.12		TfOH/TEA
	diprotonated	0.66	3.1	375, 438	0.17		TfOH

S3. Photophysical properties

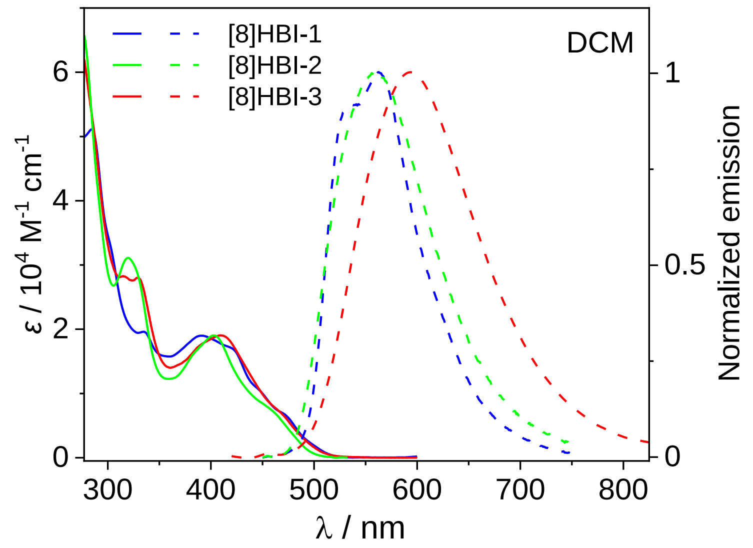

Figure S4. UV–vis and fluorescence spectra of the **[8]HBI**s in DCM (c~10^{-6} M).

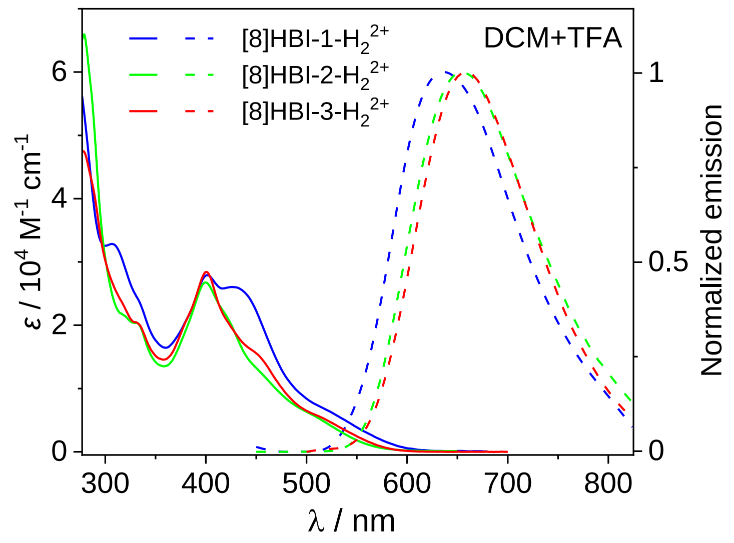

Figure S5. UV–vis and fluorescence spectra of the **[8]HBI**s in DCM (c~10^{-5} M) with TFA (0.5 M).

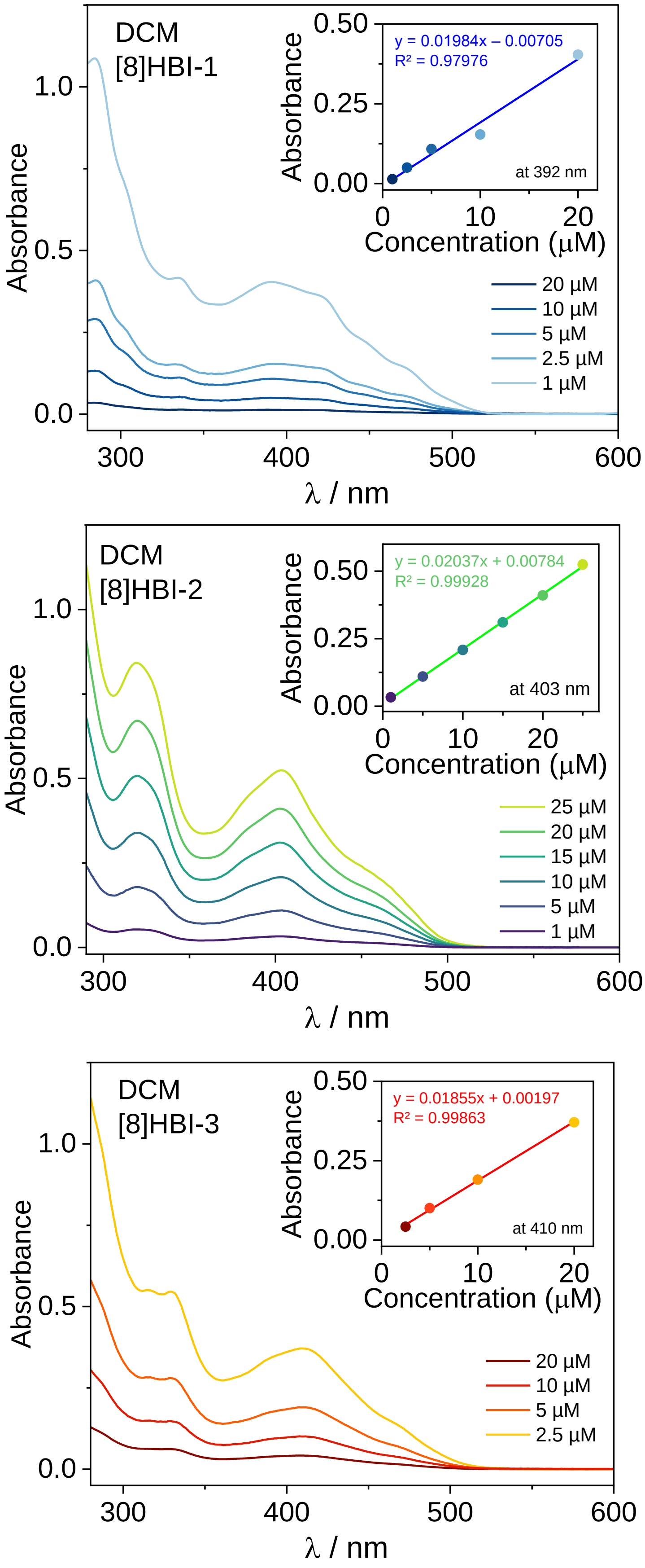

Figure S6. UV–Vis spectra of **[8]HBI**s in DCM at varying concentrations at room temperature and the corresponding linear regression for **[8]HBI-1** at 392 nm, for **[8]HBI-2** at 403 nm and for **[8]HBI-3** at 410 nm.

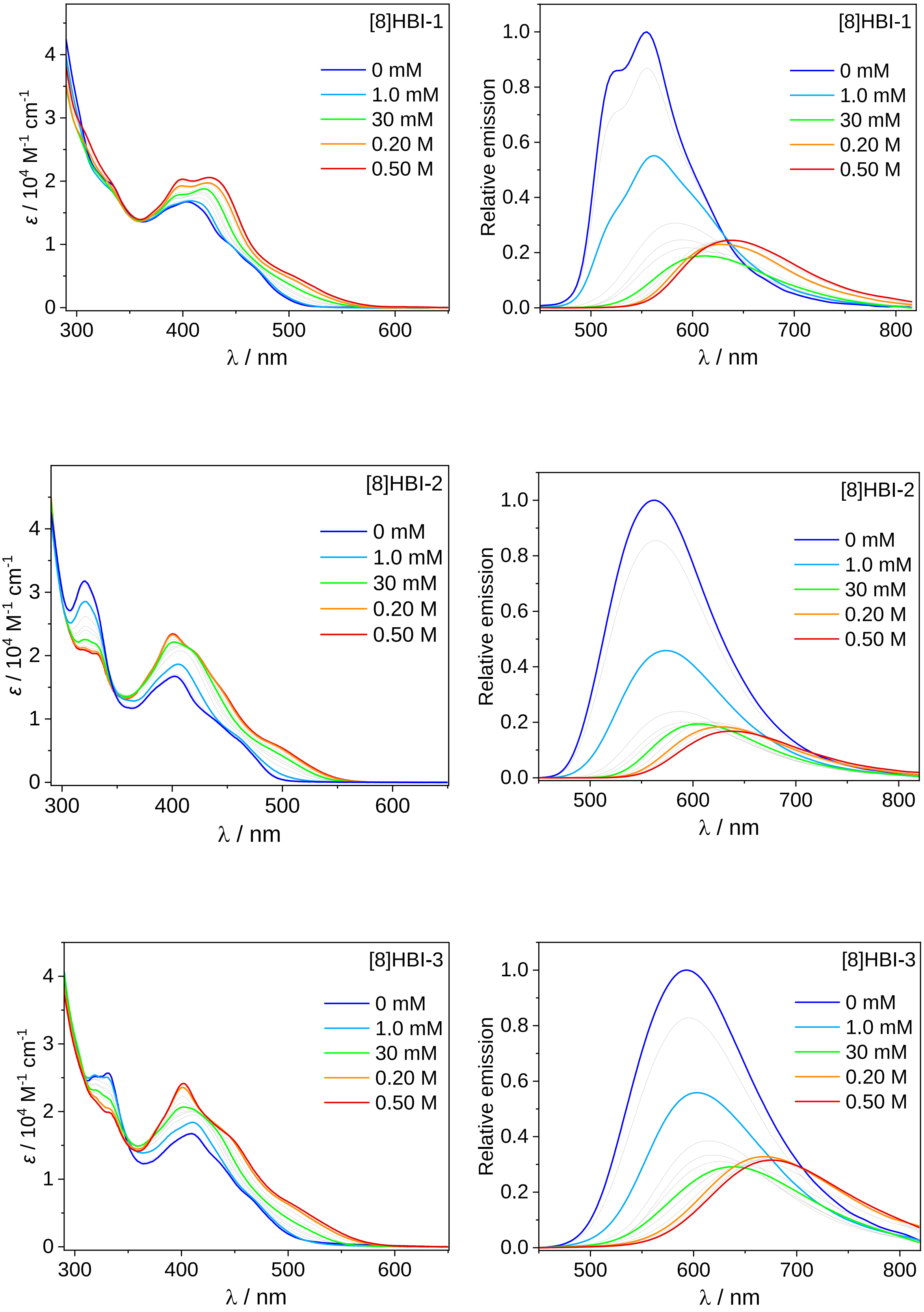

Figure S7. UV–vis and fluorescence spectra of the **[8]HBI**s (*c*~20 μM) at different TFA concentrations in toluene.

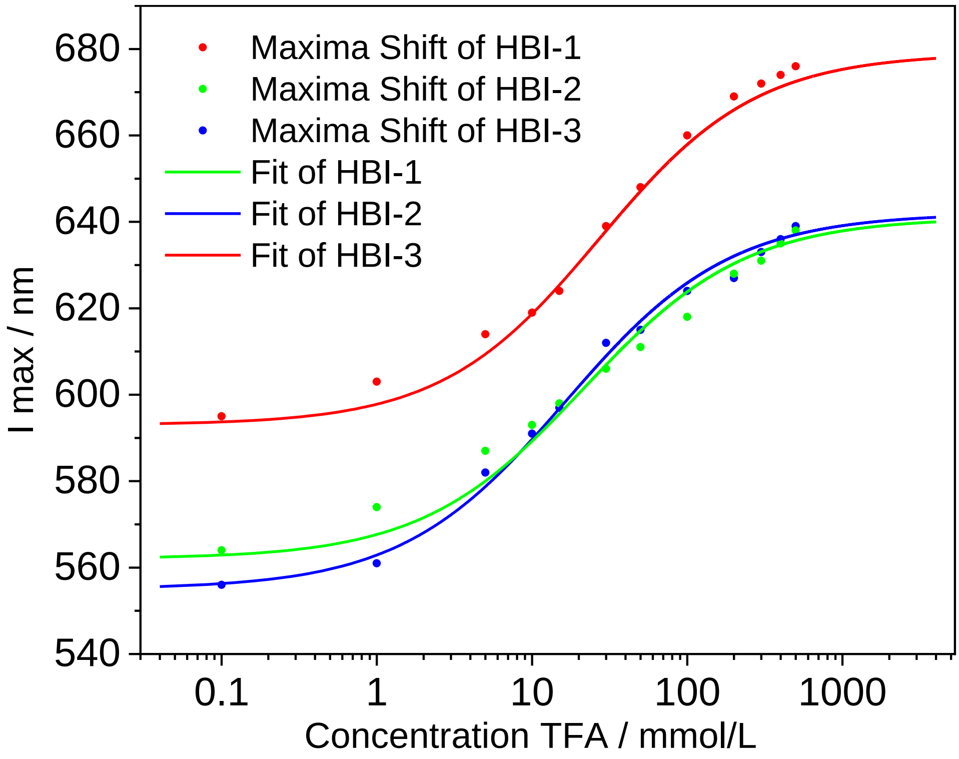

Figure S8. Plot of the fluorescence shifts from the TFA titration in toluene shown in **Figure S7**.

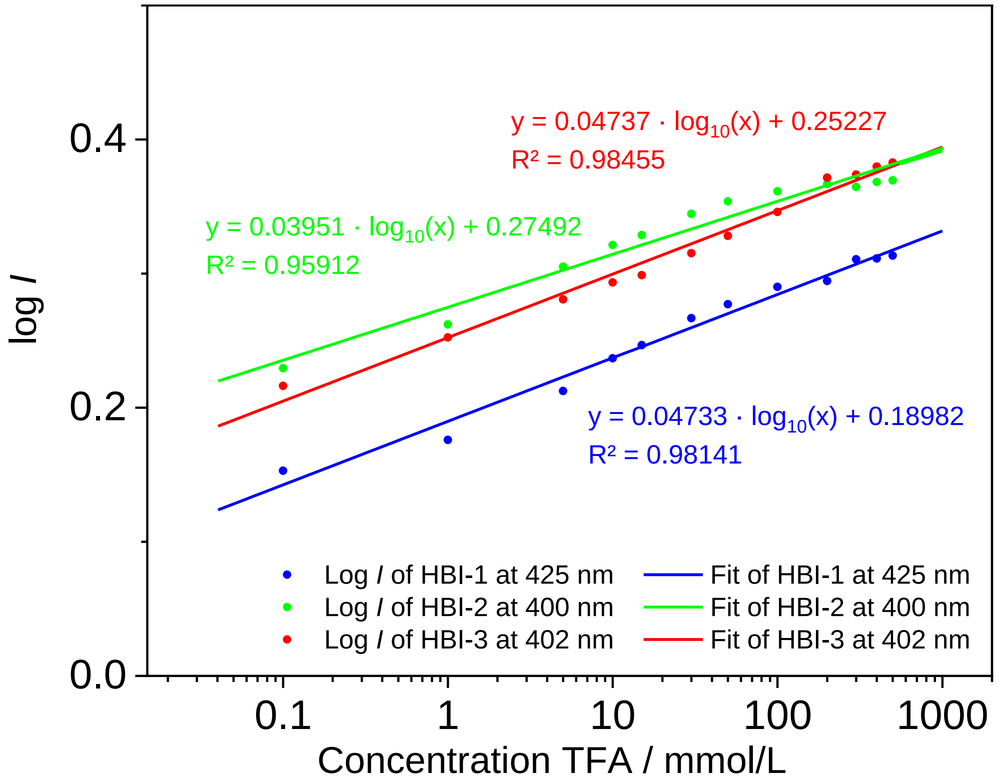

Figure S9. Plot of the change in absorption intensity at a specific wavelength during the TFA titration in toluene shown in **Figure S7**.

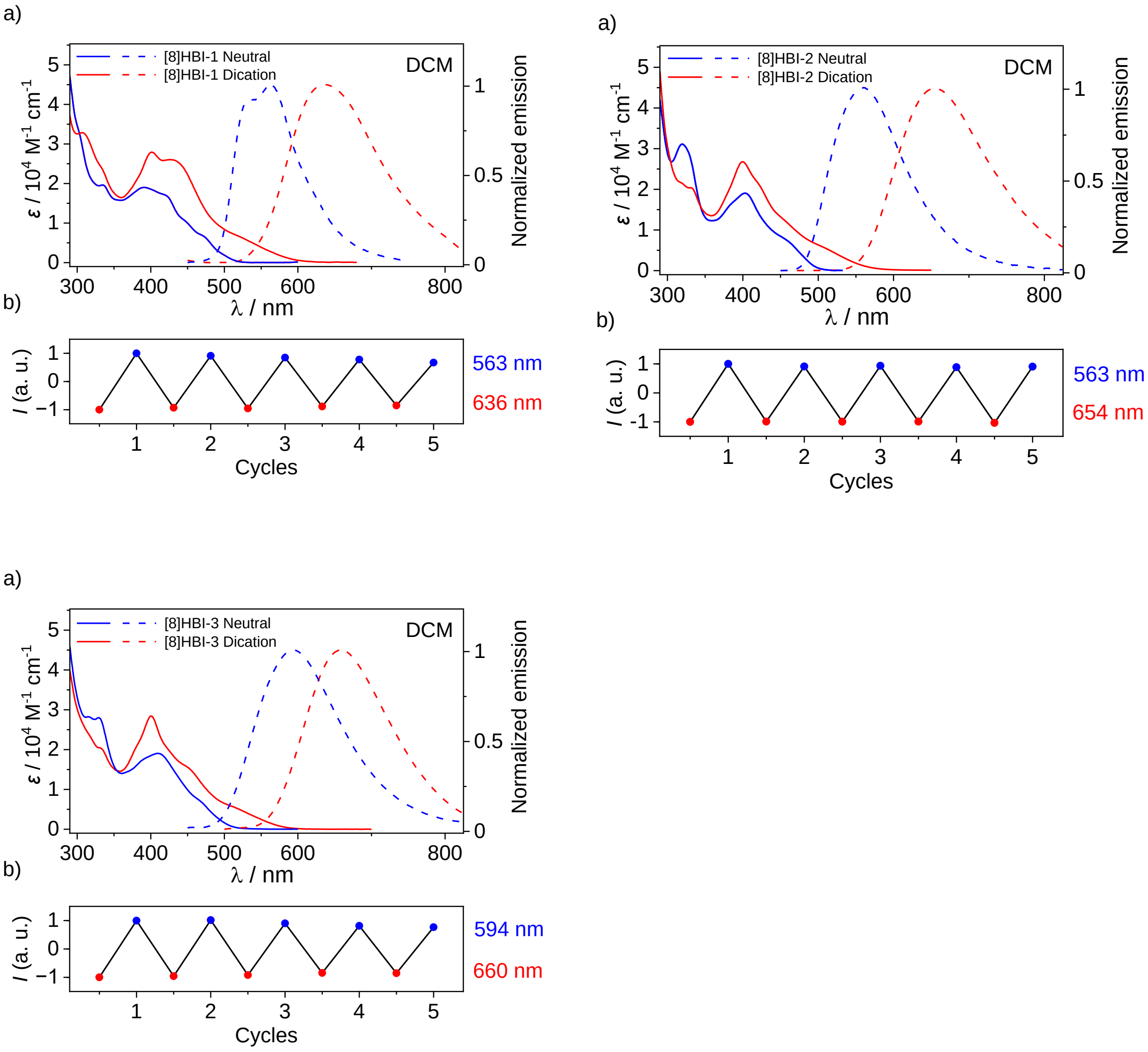

Figure S10. a) UV–vis and fluorescence spectra of **[8]HBIs** in DCM (c~20 μM) in neutral (blue) and dicationic state (red) in DCM. b) Acid/base switching cycles of the emission between neutral (blue) and dicationic state (red) through addition of TFA (0.5M) for the dicationic and pyridine (0.5M) for the neutral state. The intensity is normalized to the first emission spectra of the neutral and dicationic state.

S4. Time-resolved fluorescence decay

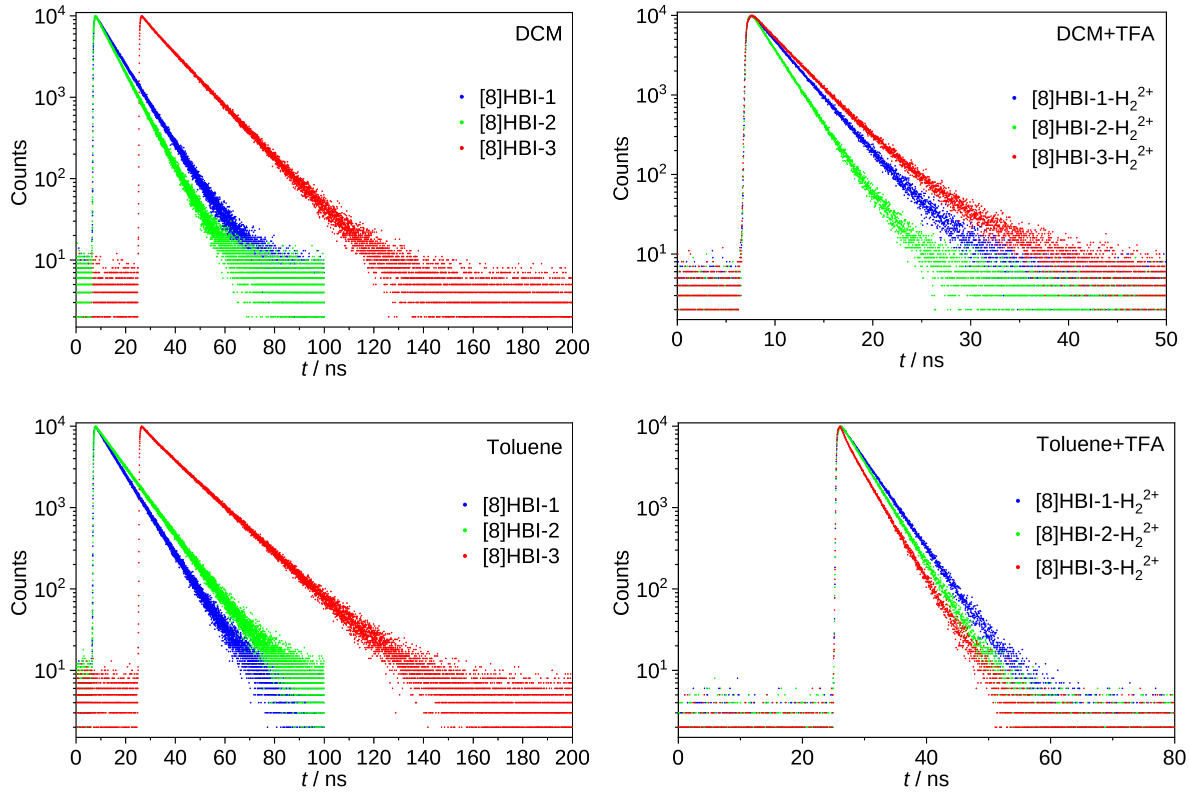

Figure S11. Comparison between the time-resolved fluorescence decays of the **[8]HBI**s in DCM and toluene with TFA (500 mM) (right) and without (left).

S5. CD and CPL spectra

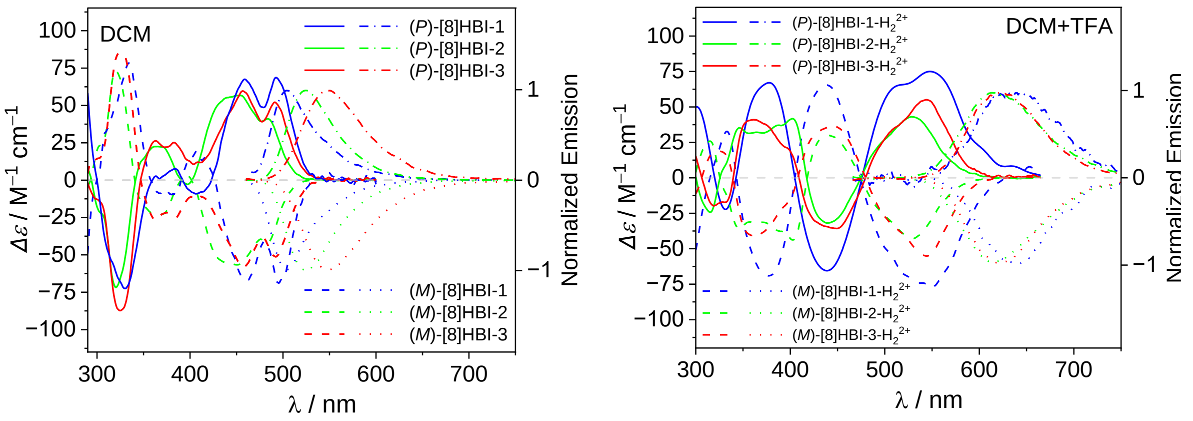

Figure S12. CD and CPL spectra of **[8]HBI**s (c~10^{-5} M) in DCM without TFA (left) and with TFA (500 mM) (right).

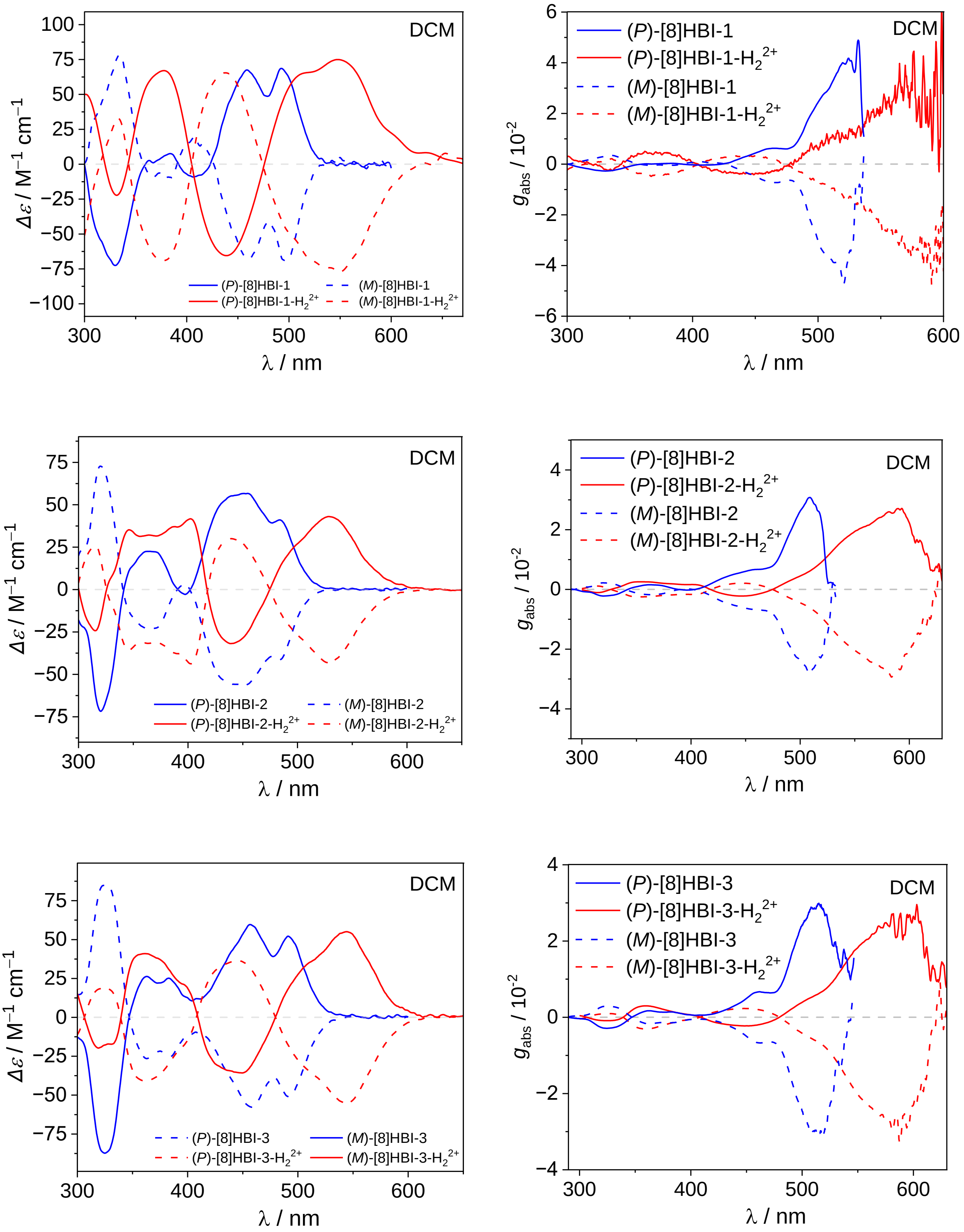

Figure S13. CD spectra (left) and g_{abs} (right) of the **[8]HBI**s (c~10^{-5} M) in DCM without TFA (blue) and with TFA (500 mM) (red).

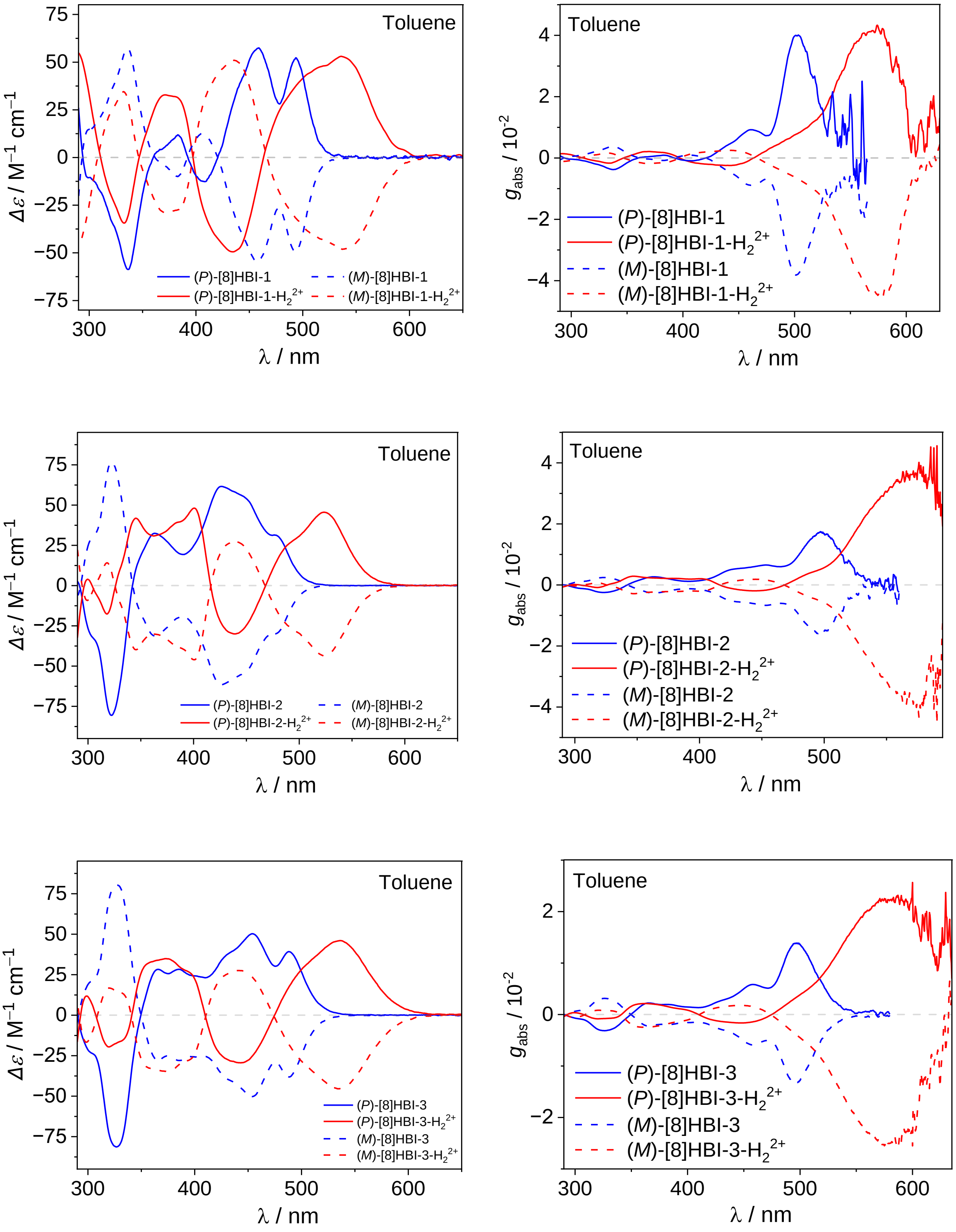

Figure S14. CD spectra (left) and g_{abs} (right) of the **[8]HBI**s (c~10^{-5} M) in toluene without TFA (blue) and with TFA (500 mM) (red).

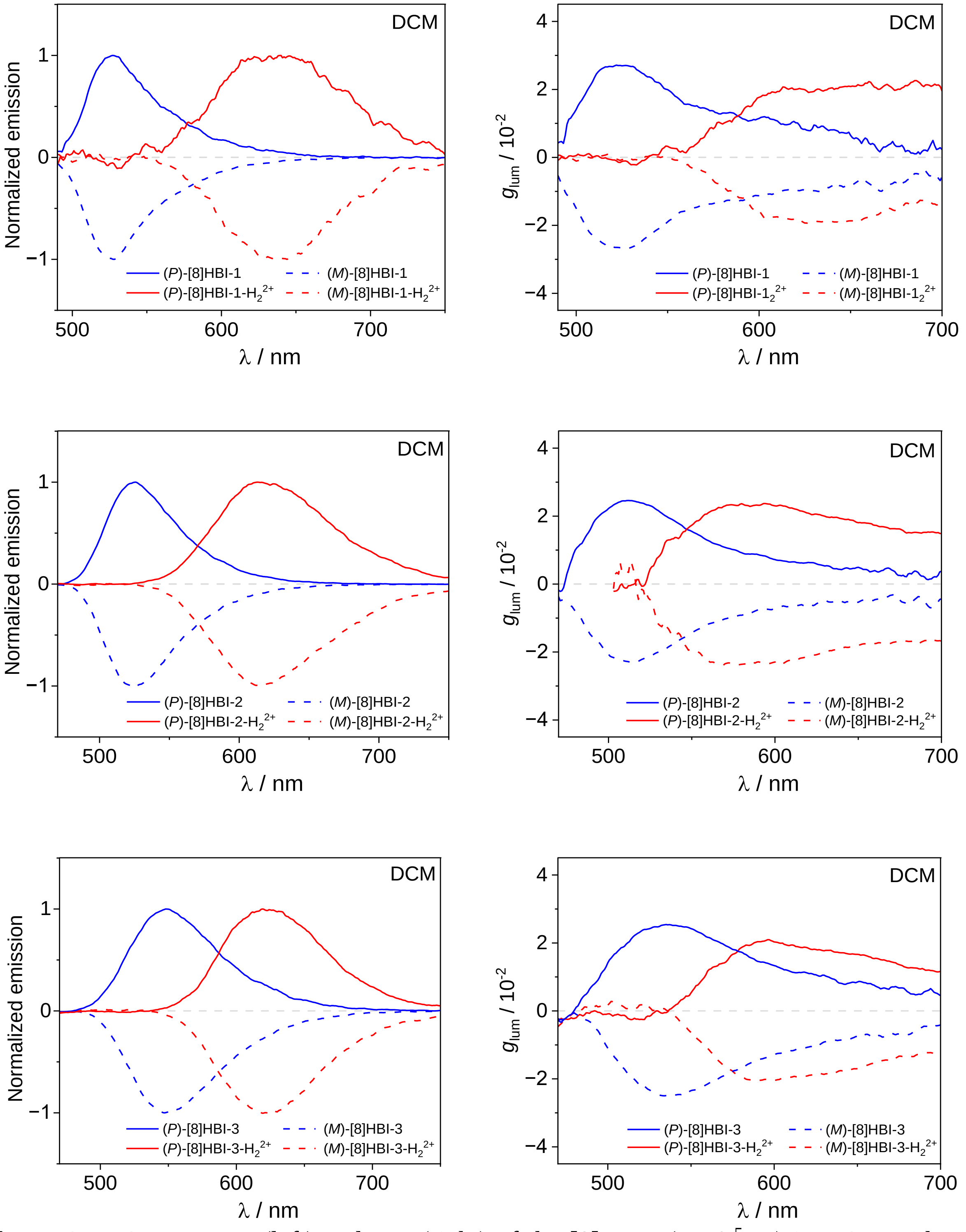

Figure S15. CPL spectra (left) and g_{lum} (right) of the **[8]HBI**s (c~10^{-5} M) in DCM without TFA (blue) and with TFA (500 mM) (red).

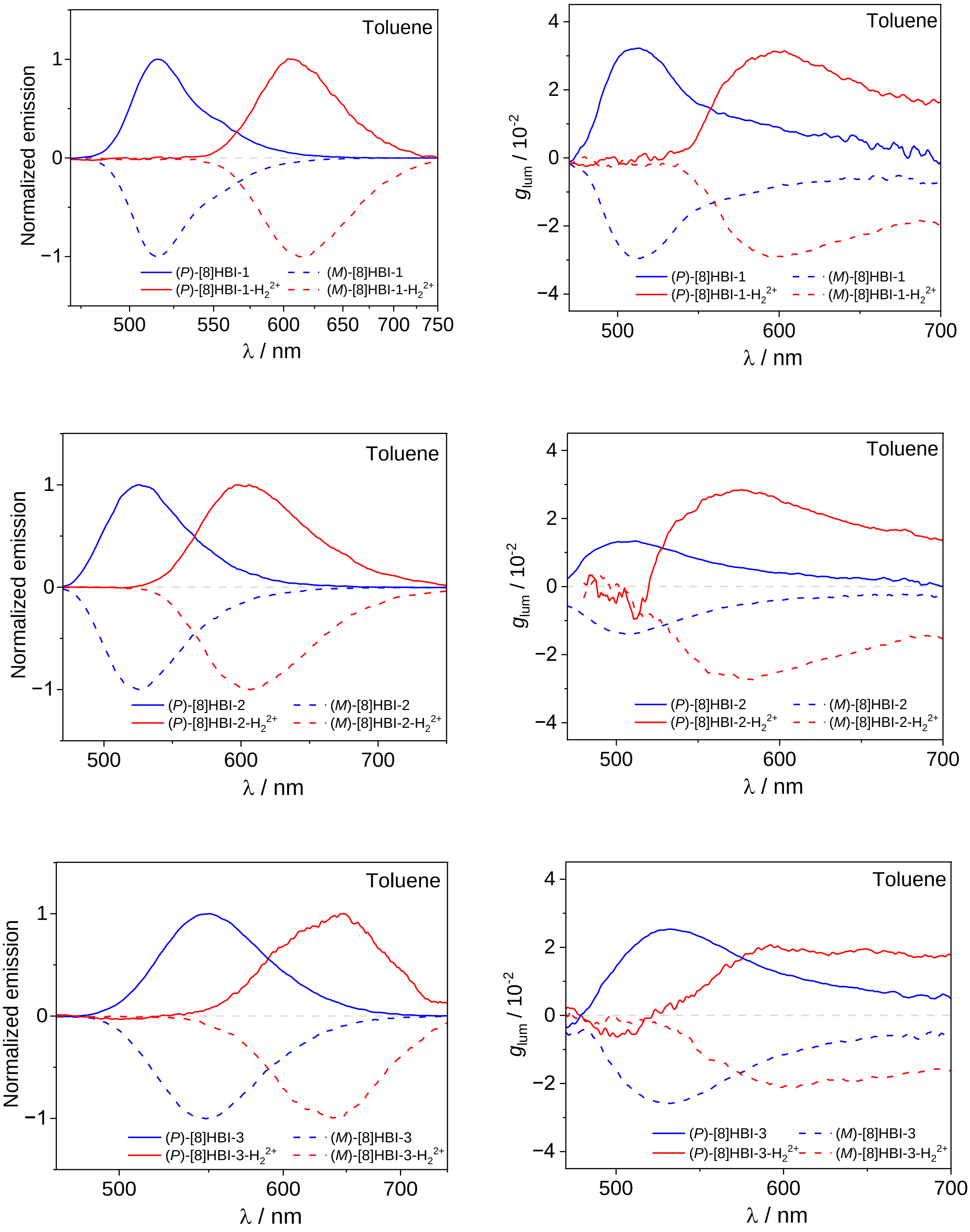

Figure S16. CPL spectra (left) and g_{lum} (right) of the **[8]HBI**s (c~10^{-5} M) in toluene without TFA (blue) and with TFA (500 mM) (red).

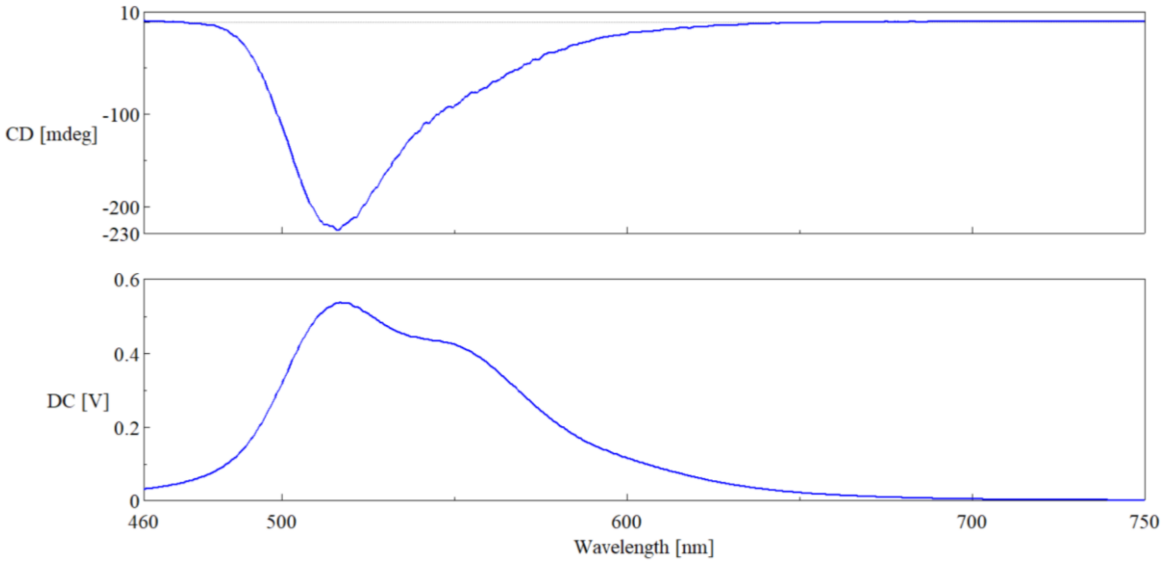

Figure S17. Raw CPL and DC Spectra of **(*M*)-[8]HBI-1** in toluene from the Jasco Spectral Manager as an example.

Table S2. Summary of optical and electrochemical data of the **[8]HBI**s.

Compound	**[8]HBI-1**				**[8]HBI-2**				**[8]HBI-3**			
Solvent	DCM	DCM +TFA[f]	Tol.	Tol. +TFA[f]	DCM	DCM +TFA[f]	Tol.	Tol. +TFA[f]	DCM	DCM +TFA[f]	Tol.	Tol. +TFA[f]
λ_{em} / nm	563	636	553	638	563	654	560	638	594	660	591	676
τ_{FL} / ns	8.82	3.09	8.79	3.94	7.43	2.30	10.4	3.47	13.1	3.56	14.7	3.19
E_g[a] / eV	2.50	2.22	2.53	2.26	2.54	2.20	2.55	2.29	2.48	2.18	2.48	2.24
Φ_{FL}	0.16	0.06	0.20	0.11	0.12	0.05	0.21	0.08	0.09	0.04	0.12	0.07
k_{FL}[b] / 10^7 s^{-1}	1.81	1.94	2.28	2.79	1.62	2.17	2.02	2.31	0.69	1.12	0.82	2.19
k_{NR}[c] / 10^7 s^{-1}	9.52	30.4	9.10	22.6	11.8	41.3	7.60	26.5	6.97	27.0	5.98	29.2
FWHM / cm^{-1}	3122	3361	3074	3140	3518	3255	3544	3310	3709	3015	3734	3389
HOMO[e] / eV	–5.86	-	-	-	–5.94	-	-	-	–5.89	-	-	-
LUMO[d] / eV	–3.36	-	-	-	–3.40	-	-	-	–3.41	-	-	-
g_{abs}[h] / 10^{-2}	4.0	3.5	3.9	4.3	2.9	2.4	1.7	3.7	3.0	2.6	1.3	2.3
g_{lum}[h] / 10^{-2}	2.7	1.9	3.1	3.0	2.4	2.2	1.4	2.8	1.9	1.7	2.5	2.1
B_{CPL}[g] / M^{-1} cm^{-1}	40.1	15.9	51.8	33.5	27.2	14.7	24.5	26.2	15.8	9.7	24.2	17.7

[a]Optical energy gap (E_g) estimated from the crossing of absorption and fluorescence spectra. [b]Rate constant for the radiative decay $k_{FL} = \Phi_{FL}/\tau_{FL}$. [c]Rate constant for the non-radiative decay $k_{NR} = (1-\Phi_{FL})/\tau_{FL}$. [d]LUMO = $-(E_{red}+5.10)$. [e]HOMO = $E_{LUMO}-E_g$. [f]TFA ≙ 500 mM TFA. [g]$B_{CPL} = \varepsilon_{abs} \times \Phi_{FL} \times g_{lum}/2$. [h]The maximum dissymmetry factors are given. The shown values are the average of the values found for both enantiomers.

S6. Cyclic voltammetry

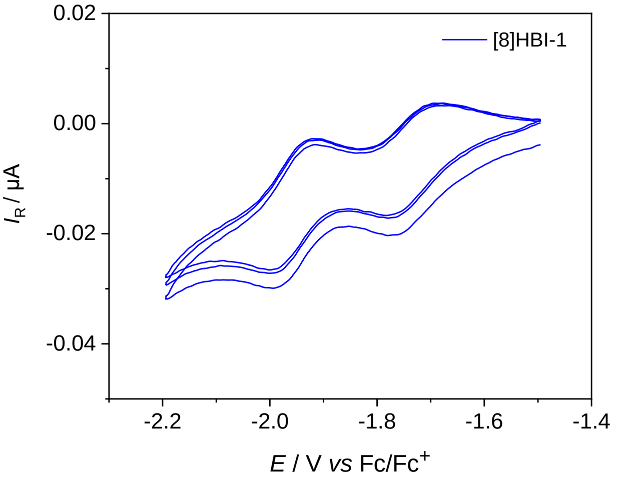

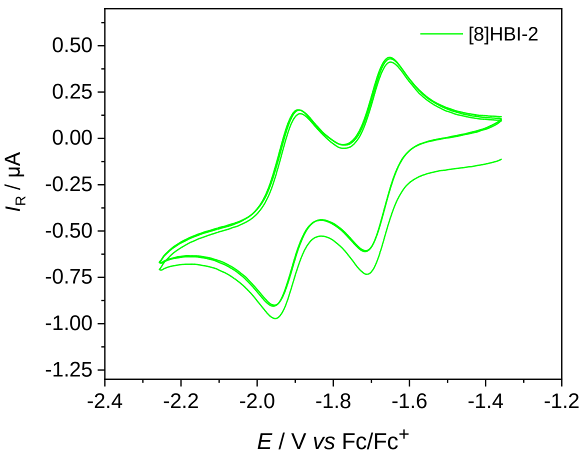

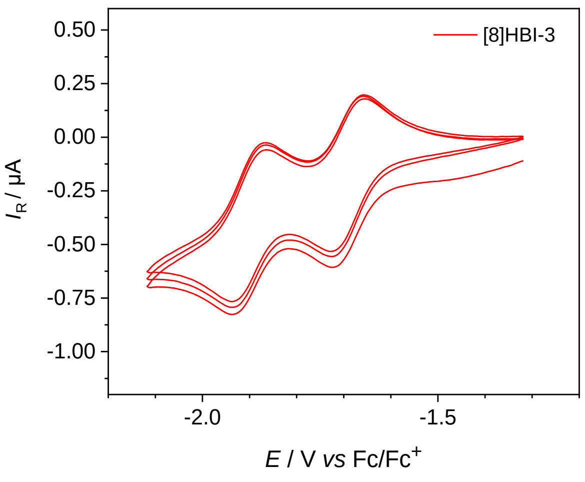

Figure S18. CV plots of **[8]HBI**s in DCM using 0.2 M [Bu_4N][PF_6] as supporting electrolyte at a scan speed of 50 mV s^{-1}.

S7. Quantum chemical calculations

DFT calculations were performed using Gaussian 16 suite.[2] Geometries were optimized using ωB97XD functional and 6-31G(d,p) basis set in the gas phase. TD-DFT calculations were performed on ωB97XD/6-31G(d,p) optimized geometries at the B3LYP/6-311G(2d,p) level. The effect of the solvent was accounted using PCM (with toluene as the solvent). SpecDis and Avogadro software were used to analyze the TD-DFT calculated spectra and POV-Ray was used to render graphical images of frontier molecular orbitals (FMOs).

Table S3. Calculated distance (measured as the average distance between the benzimidazole pyridinone-fragments) in the neutral molecules, the radical anions and the dications between the upper and lower plane.

Compound	*P* (neutral) / Å	*P* (radical anion) / Å	*P* (dication) / Å
**[8]HBI-1**	3.44	3.39	3.39
**[8]HBI-2**	3.49	3.31	3.25
**[8]HBI-3**	3.47	3.36	3.40

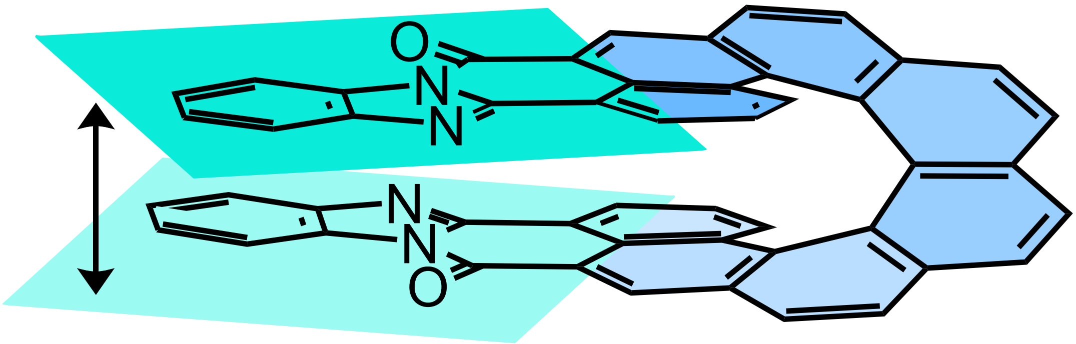

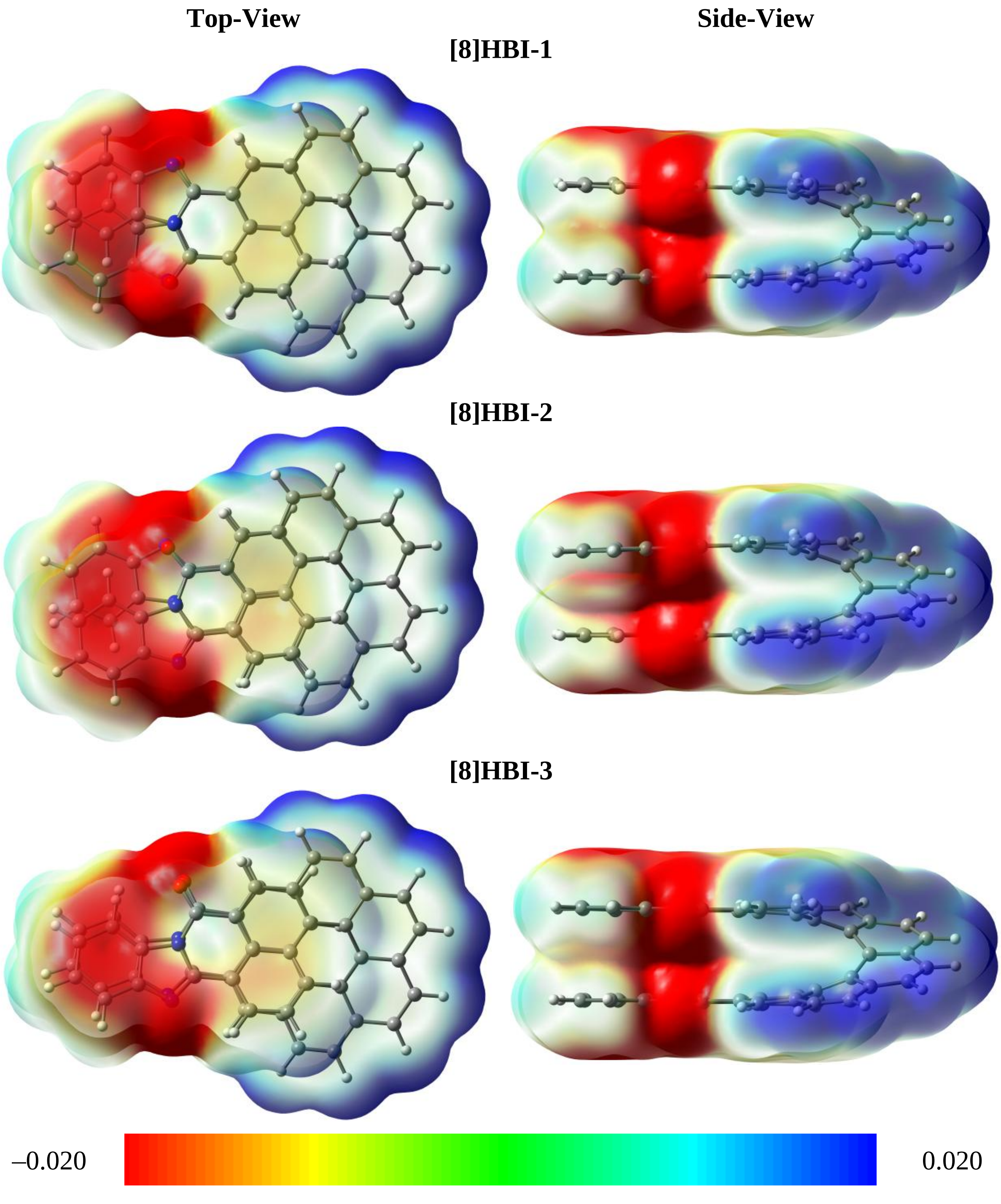

Figure S19. Calculated molecular electrostatic potential surfaces (MEPs) of **[8]HBI**s in the top (left) and side view (right).

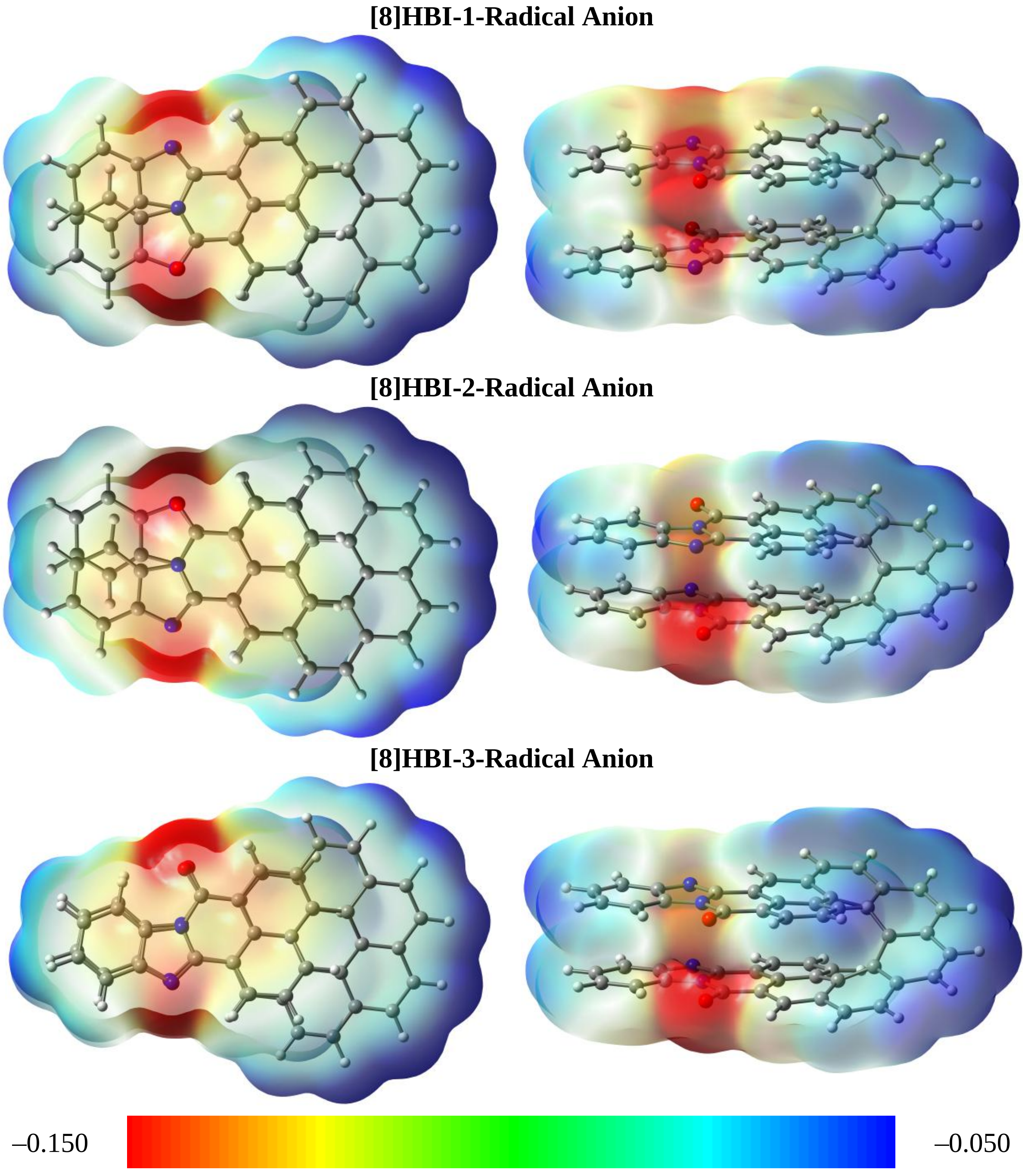

Figure S20. Calculated molecular electrostatic potential surfaces (MEPs) of radical anion of **[8]HBI**s in the top (left) and side view (right).

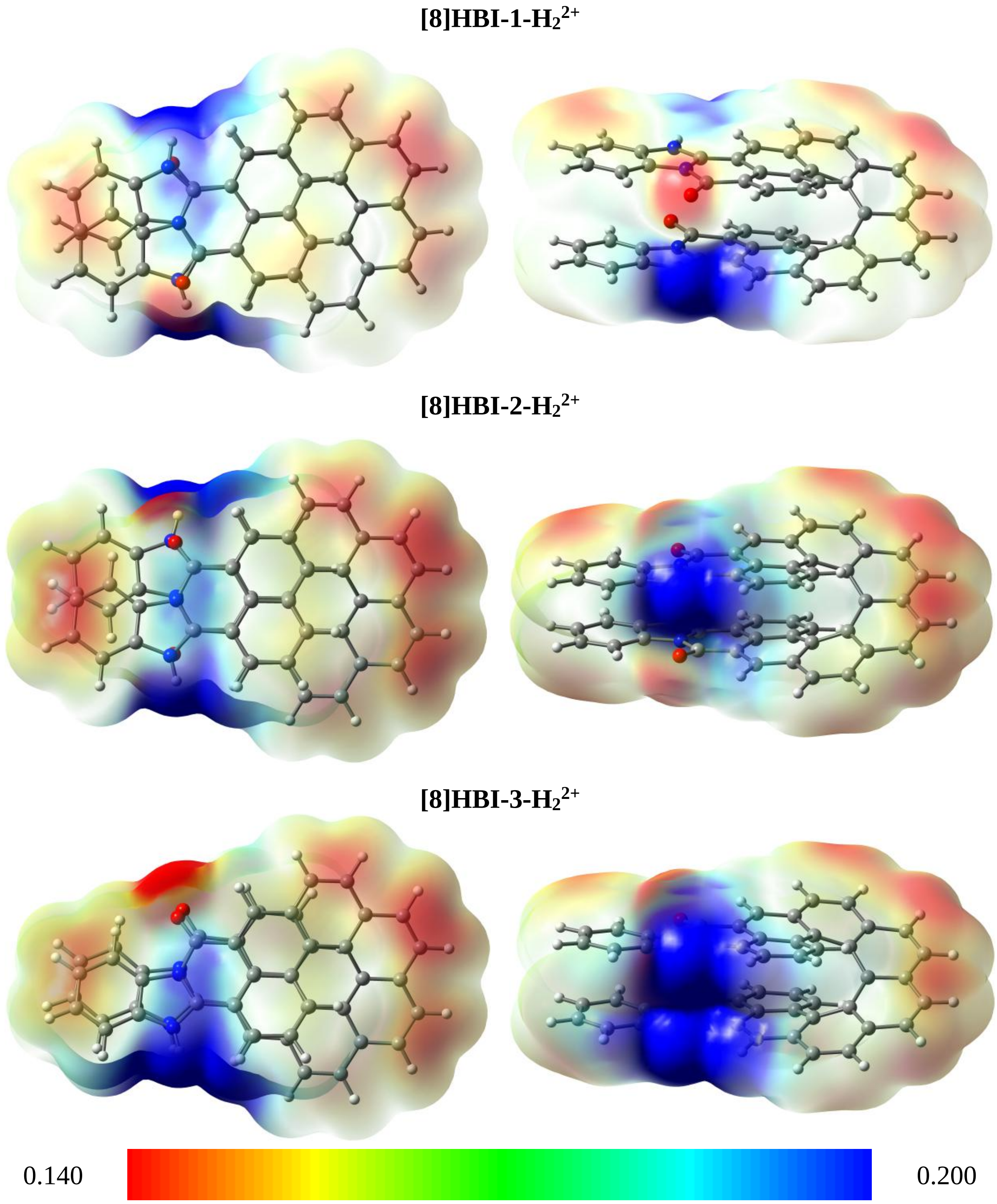

Figure S21. Calculated molecular electrostatic potential surfaces (MEPs) of doubly protonated **[8]HBI**s in the top (left) and side view (right).

Table S4. Summary of TD-DFT calculated key low-energy transitions in the UV–vis in toluene.

Excited singlet state	Wavelength	Energy	Major transitions	Contribution	Oscillator strength (f)
**[8]HBI-1**					
1	509 nm	2.44 eV	HOMO → LUMO	0.70	0.0200
2	489 nm	2.54 eV	HOMO–1 → LUMO	0.68	0.1158
			HOMO → LUMO+1	0.11	
4	447 nm	2.69 eV	HOMO–2 → LUMO	0.32	0.2419
			HOMO → LUMO+1	0.61	
5	430 nm	2.88 eV	HOMO–2 → LUMO	0.56	0.3141
			HOMO → LUMO+1	–0.30	
			HOMO → LUMO+2	–0.28	
			HOMO–2 → LUMO	0.14	
			HOMO → LUMO+1	0.22	
**[8]HBI-1-H_2^{2+}**					
1	622 nm	1.99 eV	HOMO–1 → LUMO	0.70	0.0184
2	606 nm	2.05 eV	HOMO → LUMO+1	0.70	0.0261
6	467 nm	2.65 eV	HOMO–3 → LUMO	0.64	0.4636
			HOMO–2 → LUMO+1	–0.20	
			HOMO–1 → LUMO+1	–0.20	
7	425 nm	2.92 eV	HOMO–5 → LUMO	–0.12	0.4015
			HOMO–3 → LUMO	0.17	
			HOMO–2 → LUMO+1	0.65	
			HOMO–1 → LUMO+1	–0.11	
			HOMO–1 → LUMO+2	0.11	
**[8]HBI-2**					
1	478 nm	2.59 eV	HOMO–2 → LUMO	–0.23	0.0205
			HOMO–1 → LUMO	0.66	
2	471 nm	2.63 eV	HOMO–1 → LUMO+1	–0.16	0.0745
			HOMO → LUMO	0.68	
5	444 nm	2.79 eV	HOMO–3 → LUMO	0.14	0.2360
			HOMO–1 → LUMO+1	0.66	
			HOMO → LUMO	0.14	

20	344 nm	3.60 eV	HOMO–6 → LUMO	–0.28	0.3997
			HOMO → LUMO+3	0.62	
			HOMO–4 → LUMO	–0.10	
			HOMO–2 → LUMO+2	–0.13	
			HOMO–1 → LUMO+1	0.55	
			HOMO → LUMO+1	–0.16	
			HOMO → LUMO+2	–0.29	
**[8]HBI-2-H_2^{2+}**					
1	574 nm	2.16 eV	HOMO–1 → LUMO	0.70	0.0188
2	564 nm	2.20 eV	HOMO → LUMO	0.70	0.0071
9	420 nm	2.95 eV	HOMO–3 → LUMO	0.52	0.7619
			HOMO–2 → LUMO+1	0.45	
**[8]HBI-3**					
1	510 nm	2.43 eV	HOMO → LUMO	0.69	0.0107
2	471 nm	2.63 eV	HOMO–1 → LUMO	0.23	0.1683
			HOMO → LUMO+1	0.66	
3	458 nm	2.71 eV	HOMO–3 → LUMO	–0.12	0.1587
			HOMO–2 → LUMO	–0.21	
			HOMO–1 → LUMO	0.60	
			HOMO → LUMO+1	–0.22	
			HOMO–4 → LUMO	0.65	
			HOMO–1 → LUMO	–0.14	
			HOMO → LUMO	0.14	
**[8]HBI-3-H_2^{2+}**					
1	588 nm	2.11 eV	HOMO–1 → LUMO	0.60	0.0217
			HOMO → LUMO	–0.38	
2	572 nm	2.17 eV	HOMO–1 → LUMO	0.37	0.0087
			HOMO → LUMO+1	0.59	
7	419 nm	2.96 eV	HOMO–3 → LUMO	0.50	0.4746
			HOMO–2 → LUMO+1	–0.21	
			HOMO–1 → LUMO+2	0.13	
			HOMO → LUMO+2	0.40	

Table S5. TD-DFT (B3LYP/6-311G(2d,p)) calculated parameters defining g_{abs} for the $S_0 \rightarrow S_1$ transition of the neutral and diprotonated **[8]HBI**s in toluene.

	λ^a	f^b	$\|\mu_e\|^c$	$\|\mu_m\|^d$	$\|cos\theta\|^e$	$\|R\|^f$	$\|g_{abs}\|^g$ (calc.) / 10^{-2}	$\|g_{abs}\|^g$ (obs.) / 10^{-2}
**[8]HBI-1**	509	0.0200	147.09	2.136	0.800	251.37	4.65	3.9
**[8]HBI-2**	478	0.0205	144.64	1.465	0.829	175.63	3.36	1.7
**[8]HBI-3**	510	0.0107	107.88	1.660	0.859	153.93	5.29	1.3
**[8]HBI-1-H_2^{2+}**	622	0.0184	156.36	2.082	0.990	322.21	5.27	4.3
**[8]HBI-2-H_2^{2+}**	574	0.0188	151.49	2.058	0.281	87.596	1.53	3.7
**[8]HBI-3-H_2^{2+}**	588	0.0217	164.70	1.816	0.956	285.82	4.21	2.3

^{a}Wavelength in nanometers. ^{b}Oscillator strength in atomic units. ^{c}Electric transition dipole moments for the $S_0 \rightarrow S_1$ transition in 10^{-20} esu cm. ^{d}Magnetic transition dipole moments for the $S_0 \rightarrow S_1$ transition in 10^{-20} erg G^{-1}. $^e\theta$ - angle between μ_e and μ_m in degrees. ^{f}Rotational strength in 10^{-40} erg esu cm G^{-1}. ^{g}Dimensionless values.

Table S6. TD–DFT (B3LYP/6-311G(2d,p)) calculated parameters defining g_{lum} for the $S_1 \rightarrow S_0$ transition of the neutral and diprotonated **[8]HBI**s in toluene.

	$\|\mu_e\|^a$	$\|\mu_m\|^b$	$\|cos\theta\|^c$	$\|R\|^d$	$\|g_{lum}\|^e$ (calc.) / 10^{-2}	$\|g_{lum}\|^e$ (obs.) / 10^{-2}
**[8]HBI-1**	156.88	2.040	0.772	247.08	4.01	3.1
**[8]HBI-2**	140.73	1.427	0.514	103.16	2.08	1.4
**[8]HBI-3**	145.80	1.597	0.399	92.951	1.75	2.5
**[8]HBI-1-H_2^{2+}**	213.65	2.005	0.646	376.79	2.43	3.0
**[8]HBI-2-H_2^{2+}**	39.602	0.175	1.000	6.9157	1.76	2.8
**[8]HBI-3-H_2^{2+}**	258.02	2.040	0.526	276.76	1.66	2.1

^{a}Electric transition dipole moments for the $S_1 \rightarrow S_0$ transition in 10^{-20} esu cm. ^{b}Magnetic transition dipole moments for the $S_1 \rightarrow S_0$ transition in 10^{-20} erg G^{-1}. $^c\theta$ - angle between μ_e and μ_m in degrees. ^{d}Rotational strength in 10^{-40} erg esu cm G^{-1}. ^{e}Dimensionless values.

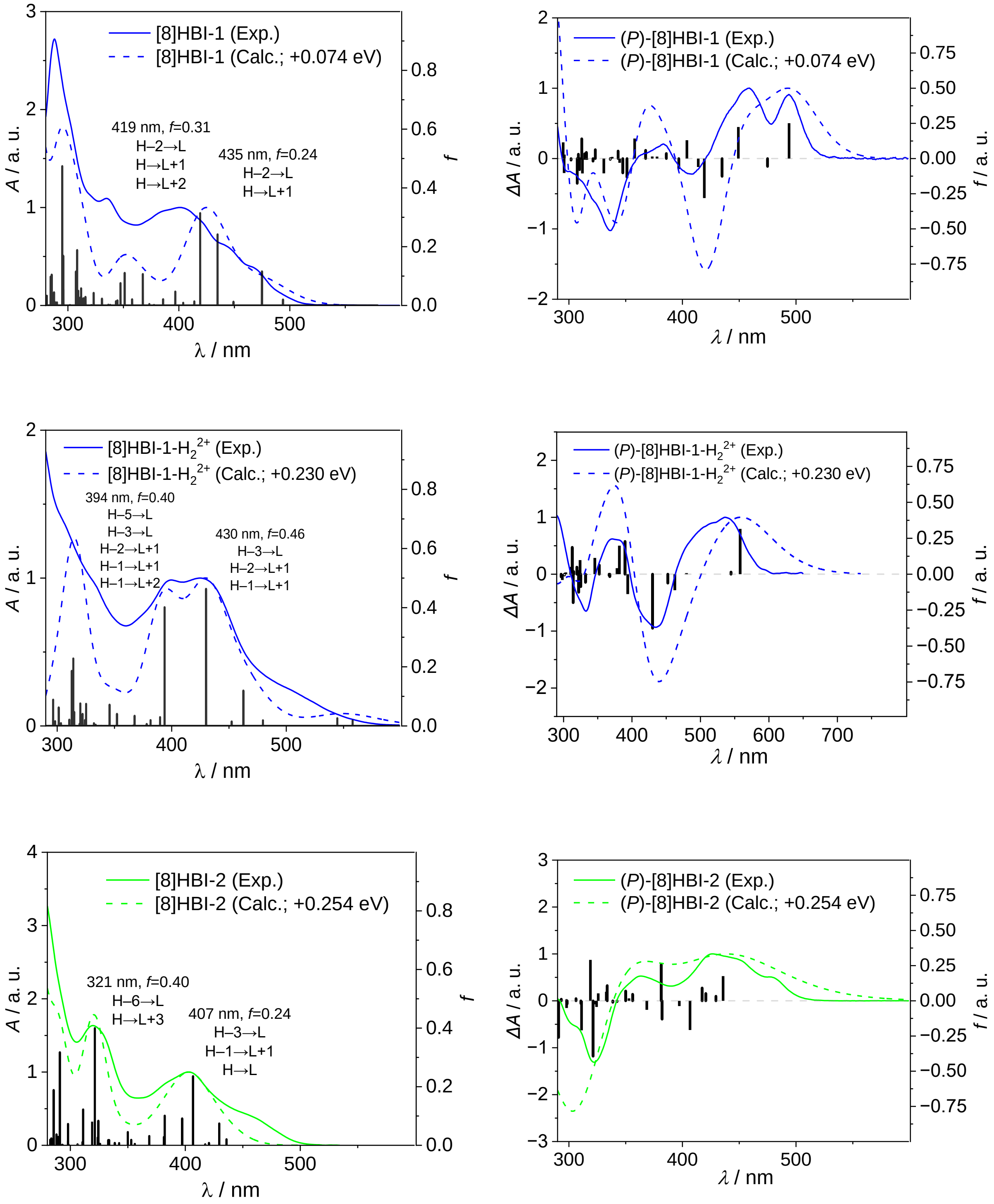
[8]HBI-1 (Exp.)
[8]HBI-1 (Calc.; +0.074 eV)
419 nm, f=0.31
H–2→L
H→L+1
H→L+2
435 nm, f=0.24
H–2→L
H→L+1
A / a. u.
f
λ / nm
(P)-[8]HBI-1 (Exp.)
(P)-[8]HBI-1 (Calc.; +0.074 eV)
ΔA / a. u.
f / a. u.
λ / nm
[8]HBI-1-H2 2+ (Exp.)
[8]HBI-1-H2 2+ (Calc.; +0.230 eV)
394 nm, f=0.40
H–5→L
H–3→L
H–2→L+1
H–1→L+1
H–1→L+2
430 nm, f=0.46
H–3→L
H–2→L+1
H–1→L+1
A / a. u.
f
λ / nm
(P)-[8]HBI-1-H2 2+ (Exp.)
(P)-[8]HBI-1-H2 2+ (Calc.; +0.230 eV)
ΔA / a. u.
f / a. u.
λ / nm
[8]HBI-2 (Exp.)
[8]HBI-2 (Calc.; +0.254 eV)
321 nm, f=0.40
H–6→L
H→L+3
407 nm, f=0.24
H–3→L
H–1→L+1
H→L
A / a. u.
f
λ / nm
(P)-[8]HBI-2 (Exp.)
(P)-[8]HBI-2 (Calc.; +0.254 eV)
ΔA / a. u.
f / a. u.
λ / nm

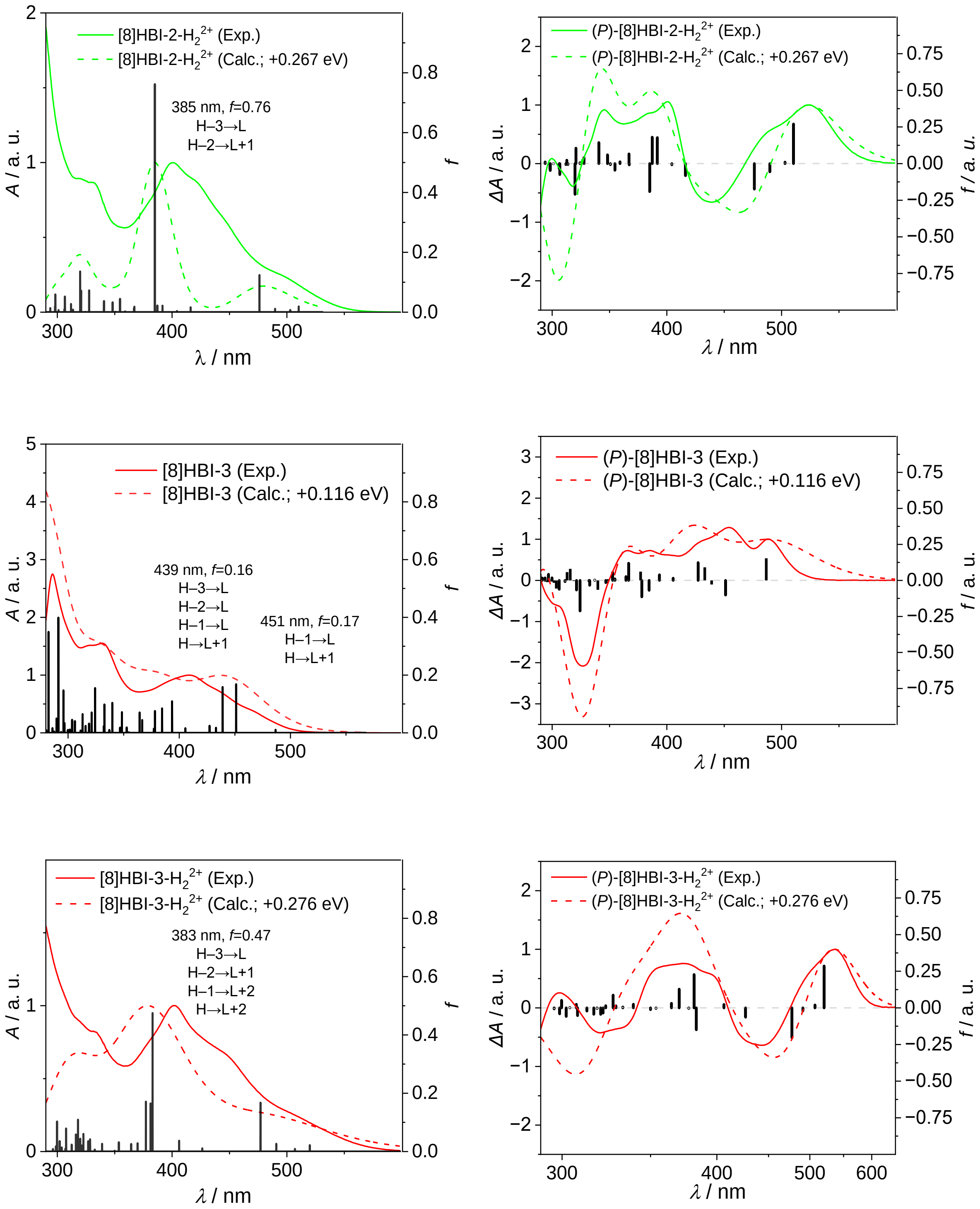

Figure S22. Comparison of experimental (solid) and TD-DFT calculated (dashed) in toluene. UV– vis absorption (left) and ECD spectra (right) along with assignments of key transitions. H = HOMO, L = LUMO, *f* = oscillator strength.

S8. Single crystal data

The crystal data of the compounds were collected on a RIGAKU XTALAB SYNERGY-R diffractometer with a HPA area detector and multi-layer mirror monochromated CuK_a radiation. The structure was solved using intrinsic phasing method[17], refined with the SHELXL program[18], and expanded using Fourier techniques. All non-hydrogen atoms were refined anisotropically. ORTEP plots were calculated and rendered with the CCDC Mercury 2024.3.1 (Build 428097) software.[19]

Table S7. Crystal data and structure refinement parameters.

Compound	***rac*-[8]HBI-1**	***rac*-[8]HBI-2**	**(*M*)-[8]HBI-3**
CCDC number	2504723	2504724	2504725
Chemical formula	$C_{50}H_{24}N_4O_2 \cdot CH_3OH$	$C_{50}H_{24}N_4O_2 \cdot CHCl_3$	$C_{50}H_{24}N_4O_2$
M_r	744.77	832.10	712.73
Crystal system, space group	Monoclinic, $P2_1/c$	Monoclinic, $P2_1/c$	Monoclinic, $P2_1$
Temperature (K)	100	100	100
a, b, c (Å)	15.5474(5), 16.0304(6), 13.9909(5)	10.777(18), 26.00(3), 13.44(2)	14.5102(4), 7.3039(1), 15.9789(3)
β (°)	104.293(3)	99.91(6)	90, 106.331(2), 90
V (Å^3)	3379.0(2)	3711(10)	1625.13(6)
Z	4	4	2
Radiation type	Cu Kα	Cu Kα	Cu Kα
μ (mm^{-1})	0.74	2.65	0.72
Crystal size (mm)	0.07 × 0.07 × 0.01	0.55 × 0.10 × 0.05	0.25 × 0.06 × 0.01
T_{min}, T_{max}	0.911, 1.000	0.308, 1.000	0.644, 1.000
No. of measured, independent and observed [$I > 2\sigma(I)$] reflections	21013, 6625, 4331	35361, 7066, 6598	20219, 6271, 5310
R_{int}	0.044	0.025	0.036
$(\sin \theta/\lambda)_{max}$ (Å^{-1})	0.628	0.617	0.627
$R[F^2 > 2\sigma(F^2)]$, $wR(F^2)$, S	0.053, 0.145, 1.01	0.060, 0.152, 1.10	0.053, 0.159, 1.09
No. of reflections	6625	7066	6271
No. of parameters	525	541	505
No. of restraints			1
Absolute structure parameter			0.2(2)
$\Delta\rho_{max}$, $\Delta\rho_{min}$ (e Å^{-3})	0.31, −0.29	0.66, −0.62	0.24, −0.28

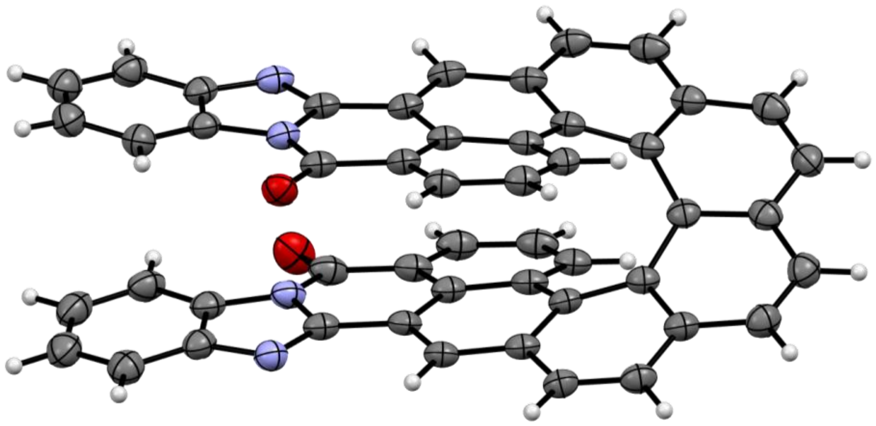

Figure S23. Thermal ellipsoids of ***rac*-[8]HBI-1** at the 50% probability level.

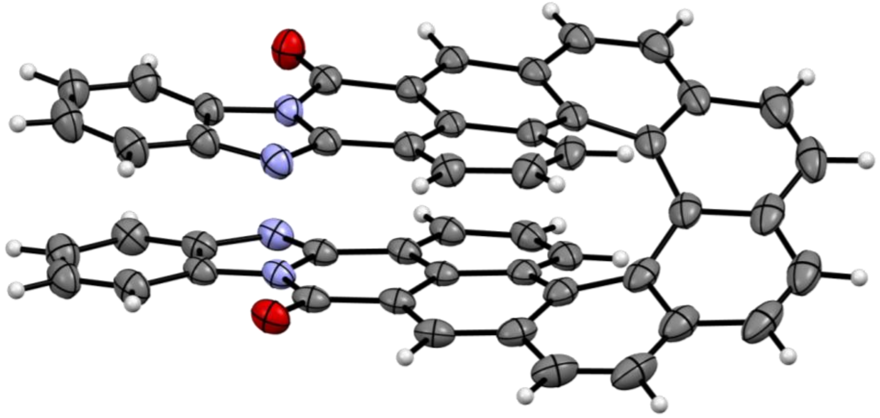

Figure S24. Thermal ellipsoids of ***rac*-[8]HBI-2** at the 50% probability level.

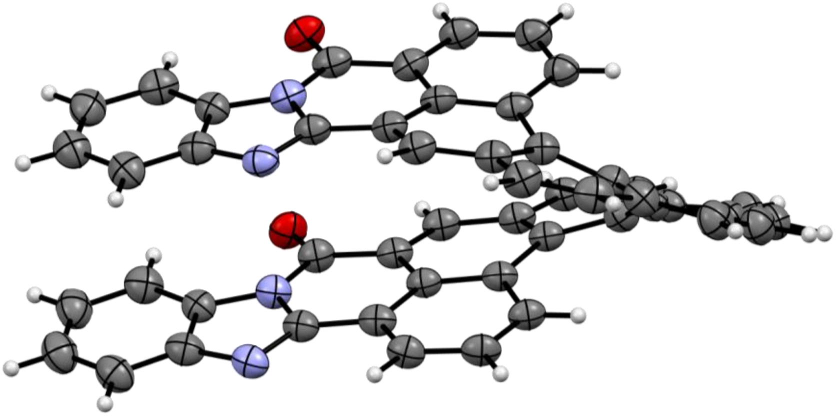

Figure S25. Thermal ellipsoids of **(*M*)-[8]HBI-3** at the 50% probability level.

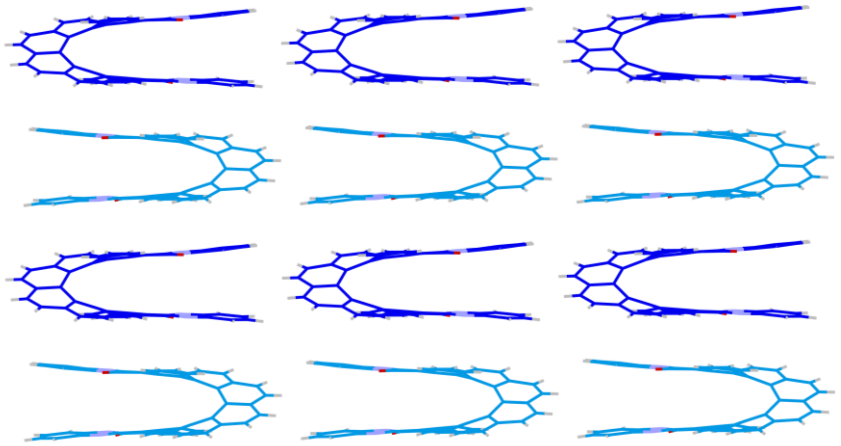

Figure S26. Crystal packing of ***rac*-[8]HBI-1**. **(*M)*-[8]HBI-1** is dark und **(*P)*-[8]HBI-1** is light blue. The molecular layers of a single enantiomer are arranged in stacks, with alternating enantiomers with a 180° offset within the stacks.

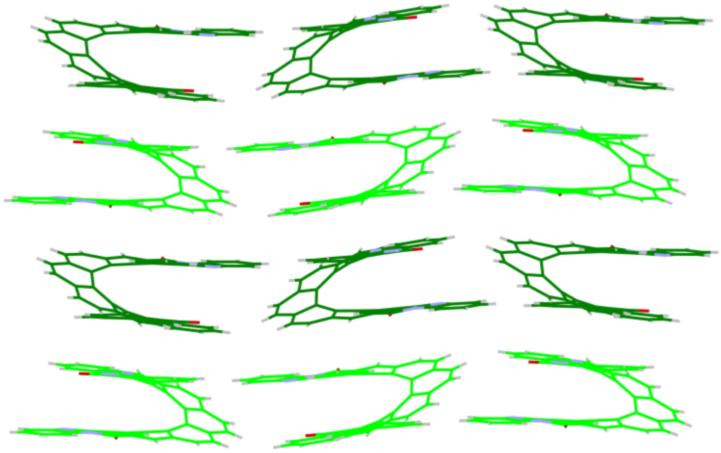

Figure S27. Crystal packing of ***rac*-[8]HBI-2**. **(*M)*-[8]HBI-2** is dark und **(*P)*-[8]HBI-2** is light green. The molecular layers of a single enantiomer are arranged in stacks, with alternating enantiomers with a 180° offset within the stacks and a 90° offset between the stacks.

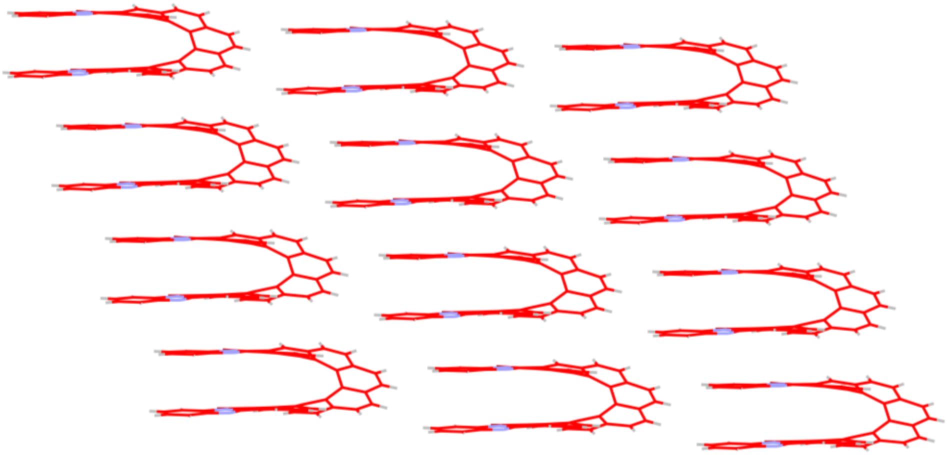

Figure S28. Crystal packing of **(*M*)-[8]HBI-3** in dark red. The molecules are arranged in layers with a interlayer shift.

S9. NMR spectra

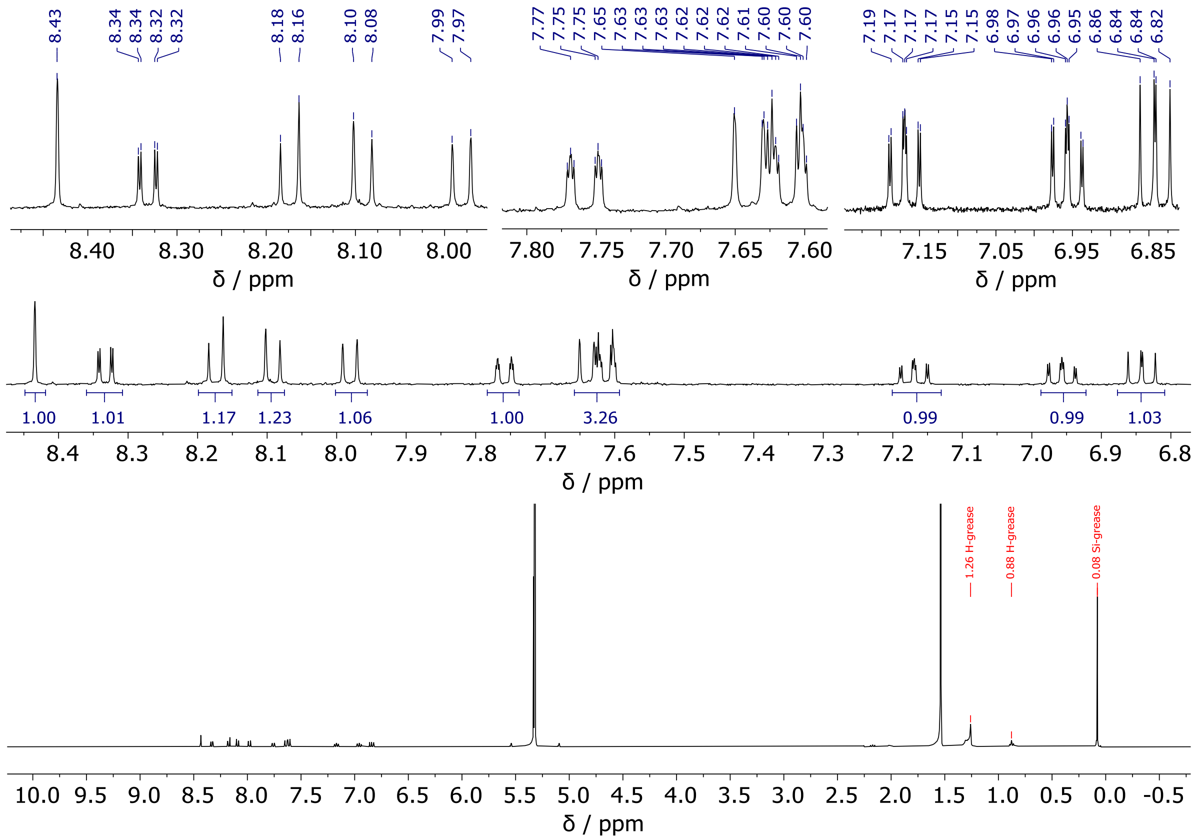

Figure S29. ^{1}H NMR spectra (400.1 MHz, CD_2Cl_2) of **[8]HBI-1**.

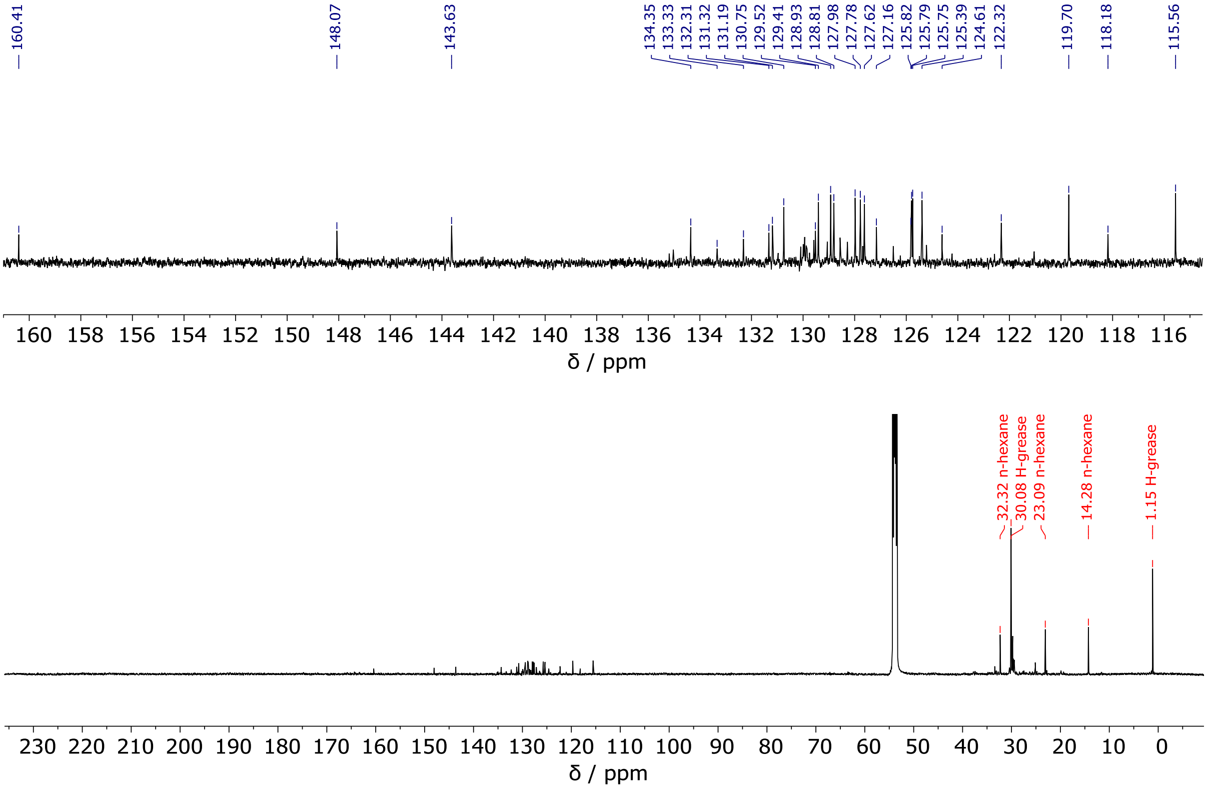

Figure S30. ^{13}C NMR spectra (150.9 MHz, CD_2Cl_2) of **[8]HBI-1**.

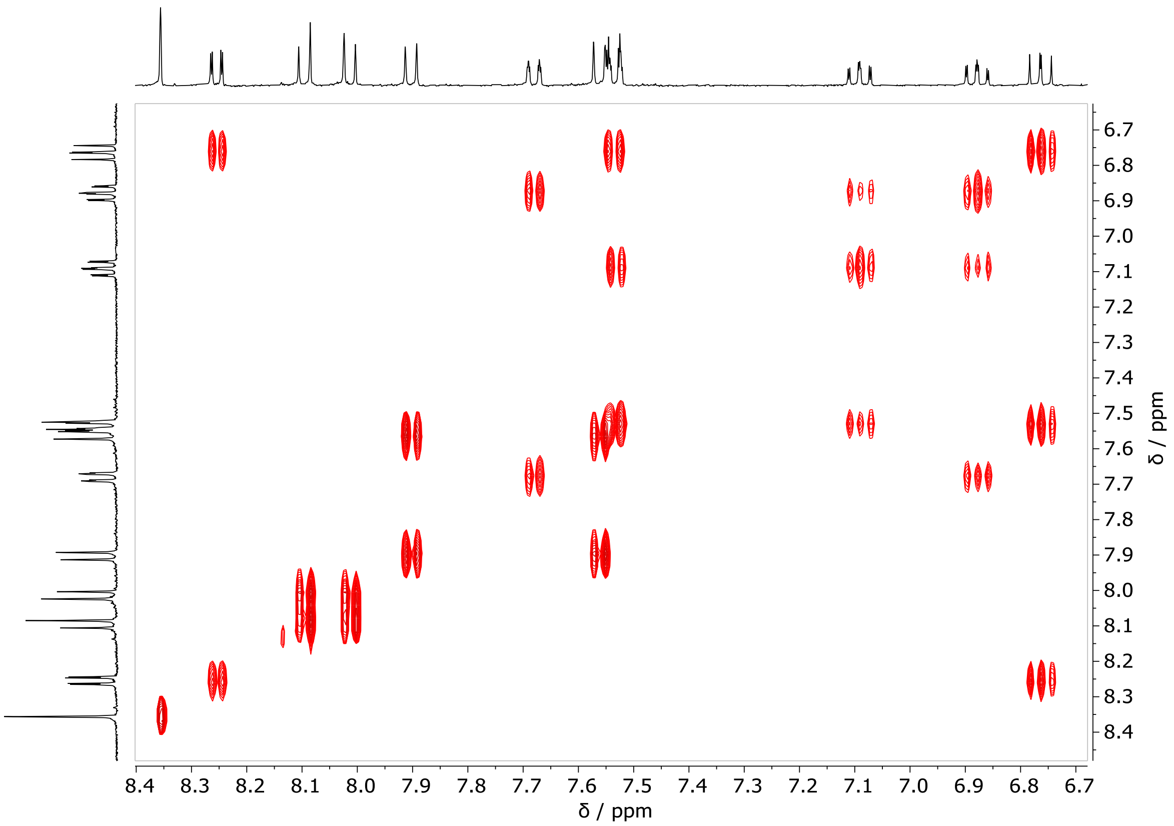

Figure S31. ^{1}H–^{1}H COSY NMR spectrum of **[8]HBI-1** in CD_2Cl_2.

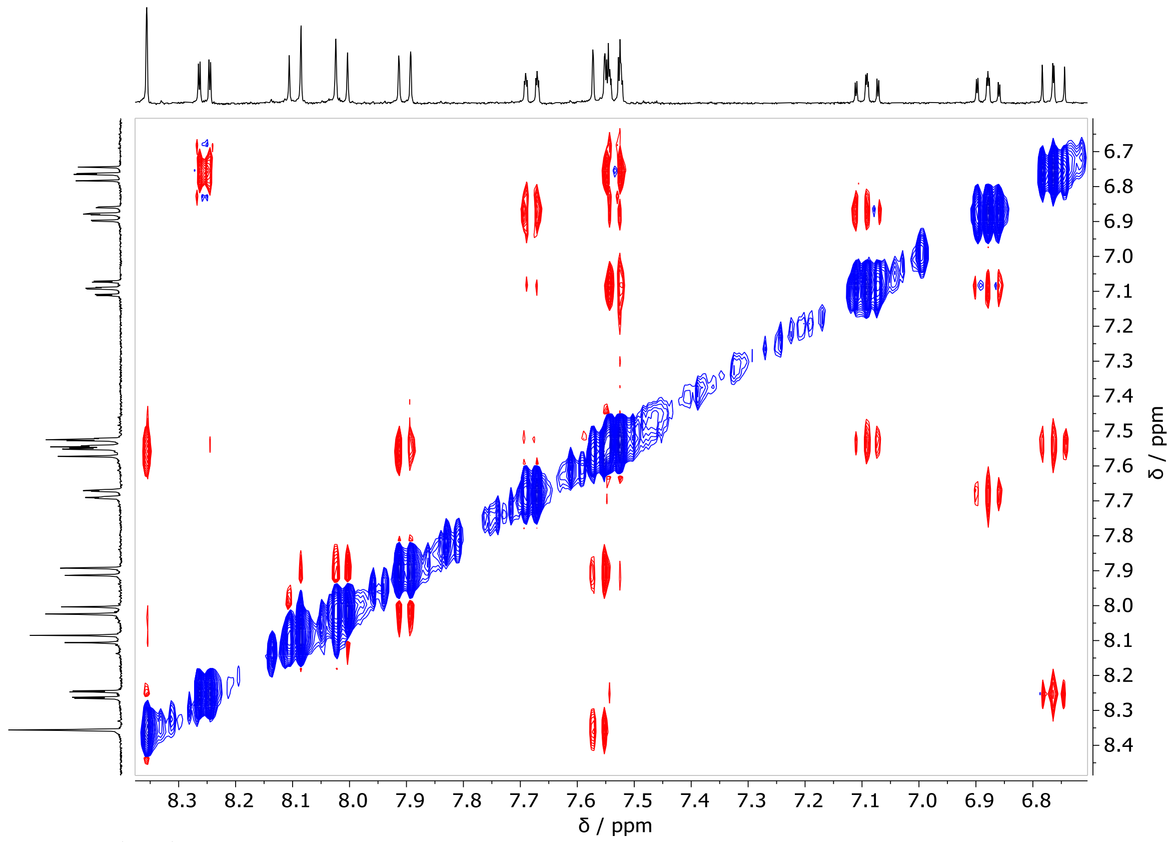

Figure S32. ^{1}H–^{1}H NOESY NMR spectrum of **[8]HBI-1** in CD_2Cl_2.

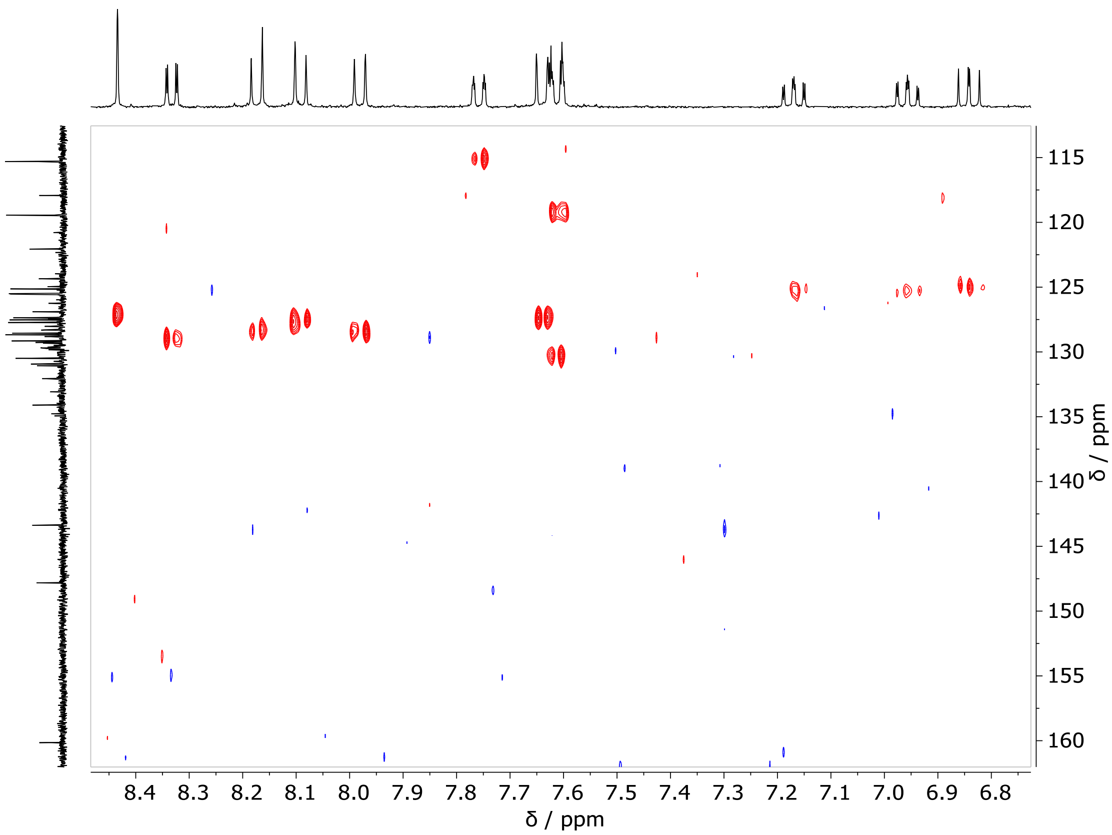

Figure S33. ^{1}H–^{13}C HSQC NMR spectrum of **[8]HBI-1** in CD_2Cl_2.

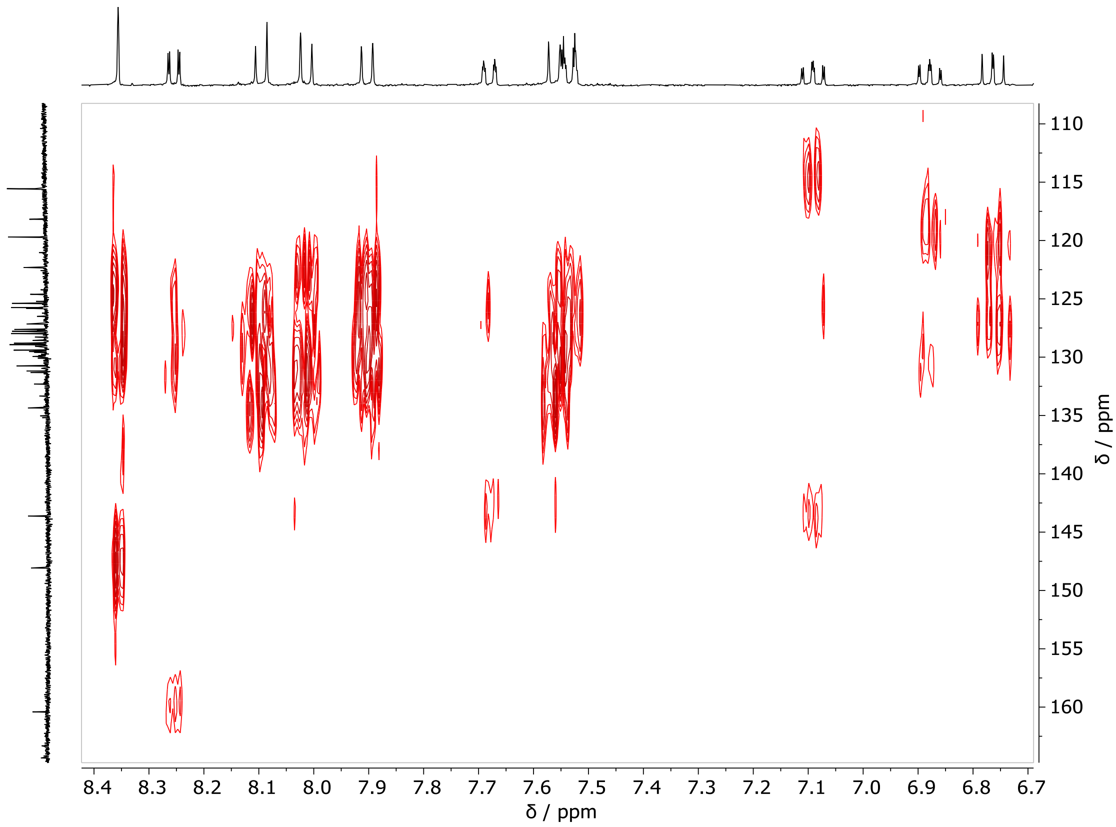

Figure S34. ^{1}H–^{13}C HMBC NMR spectrum of **[8]HBI-1** in CD_2Cl_2.

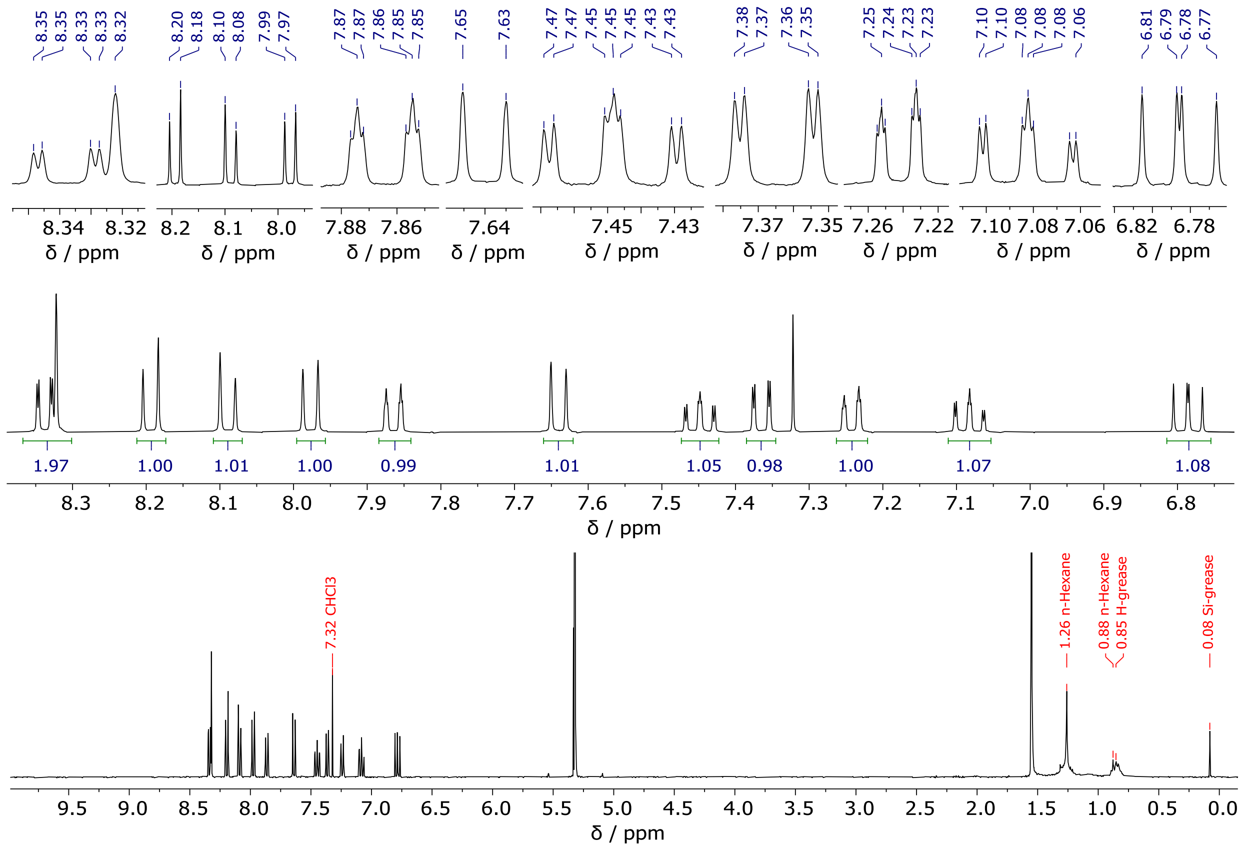

Figure S35. ^{1}H NMR spectra (400.1 MHz, CD_2Cl_2) of **[8]HBI-2**.

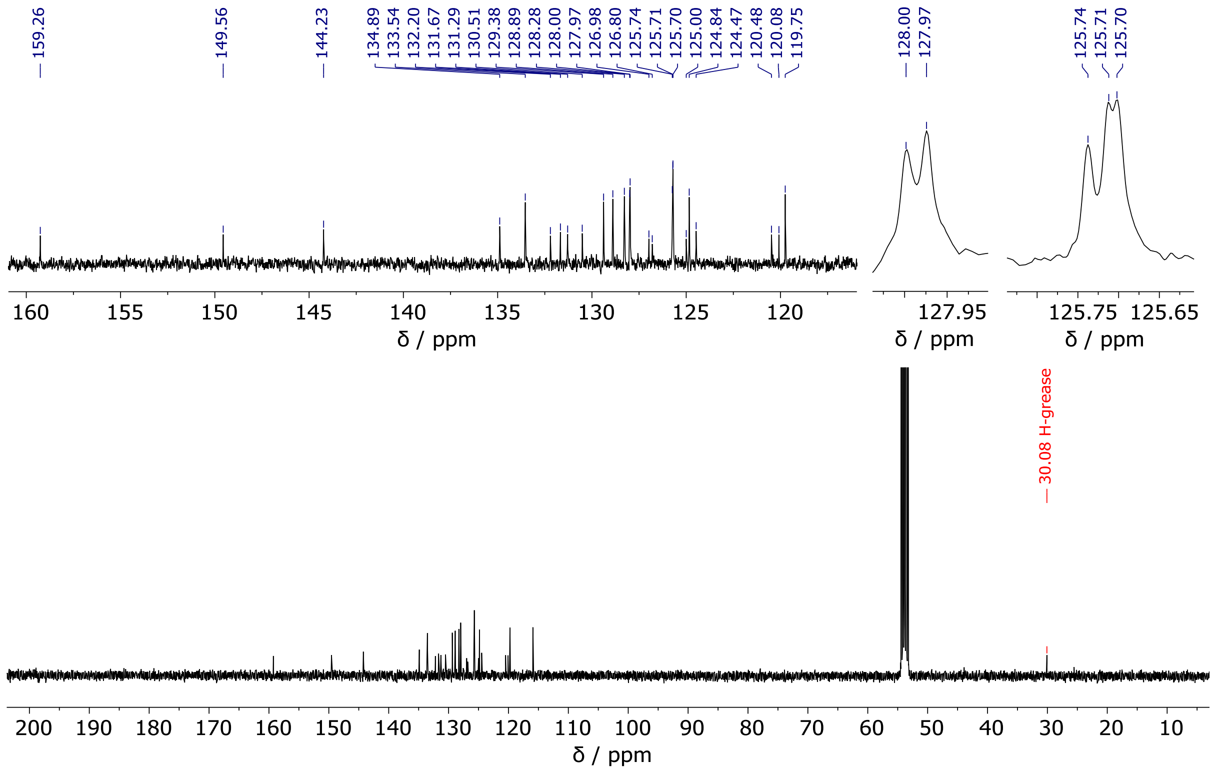

Figure S36. ^{13}C NMR spectra (100.6 MHz, CD_2Cl_2) of **[8]HBI-2**.

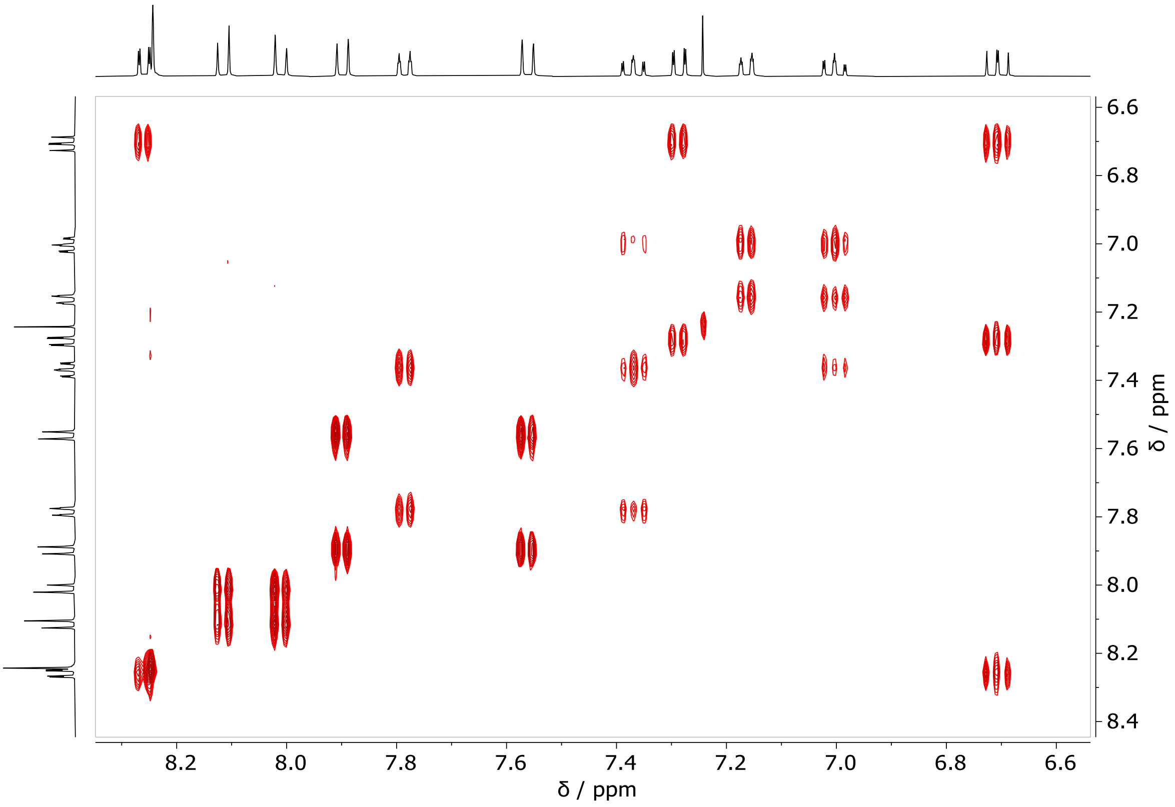

Figure S37. ^1H–^1H COSY NMR spectrum of **[8]HBI-2** in CD_2Cl_2.

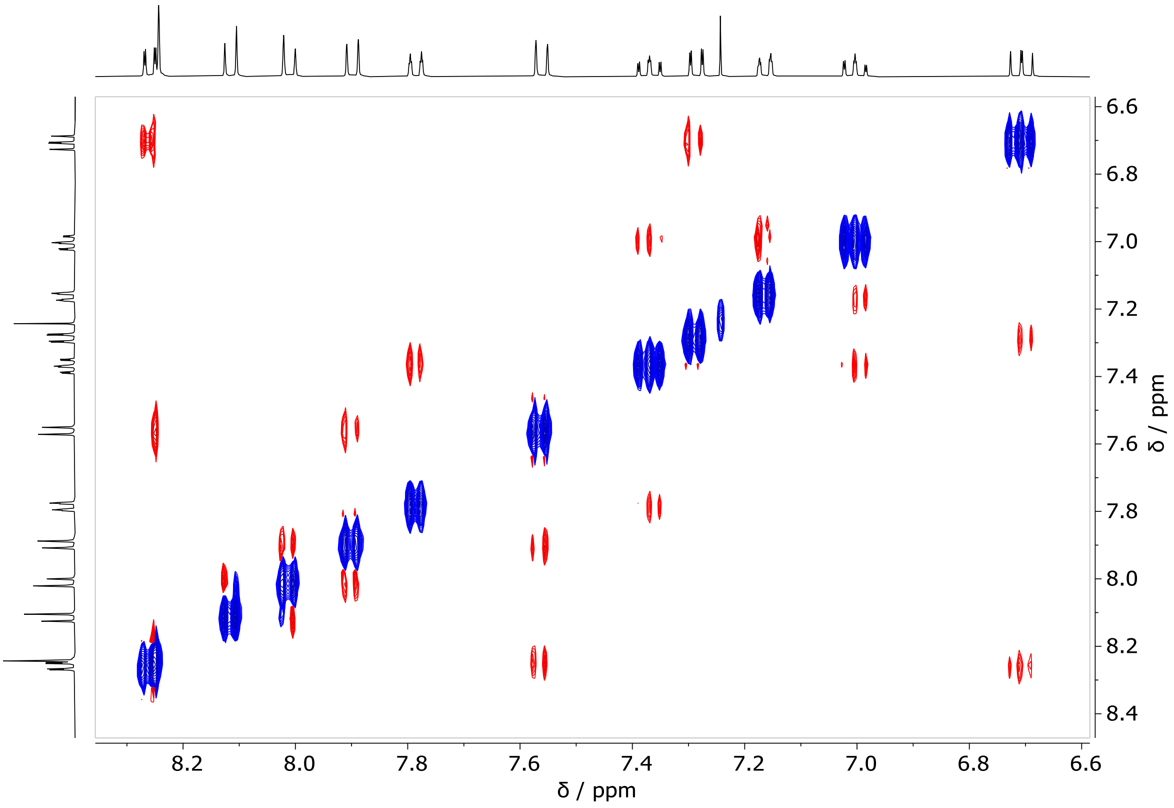

Figure S38. ^1H–^1H NOESY NMR spectrum of **[8]HBI-2** in CD_2Cl_2.

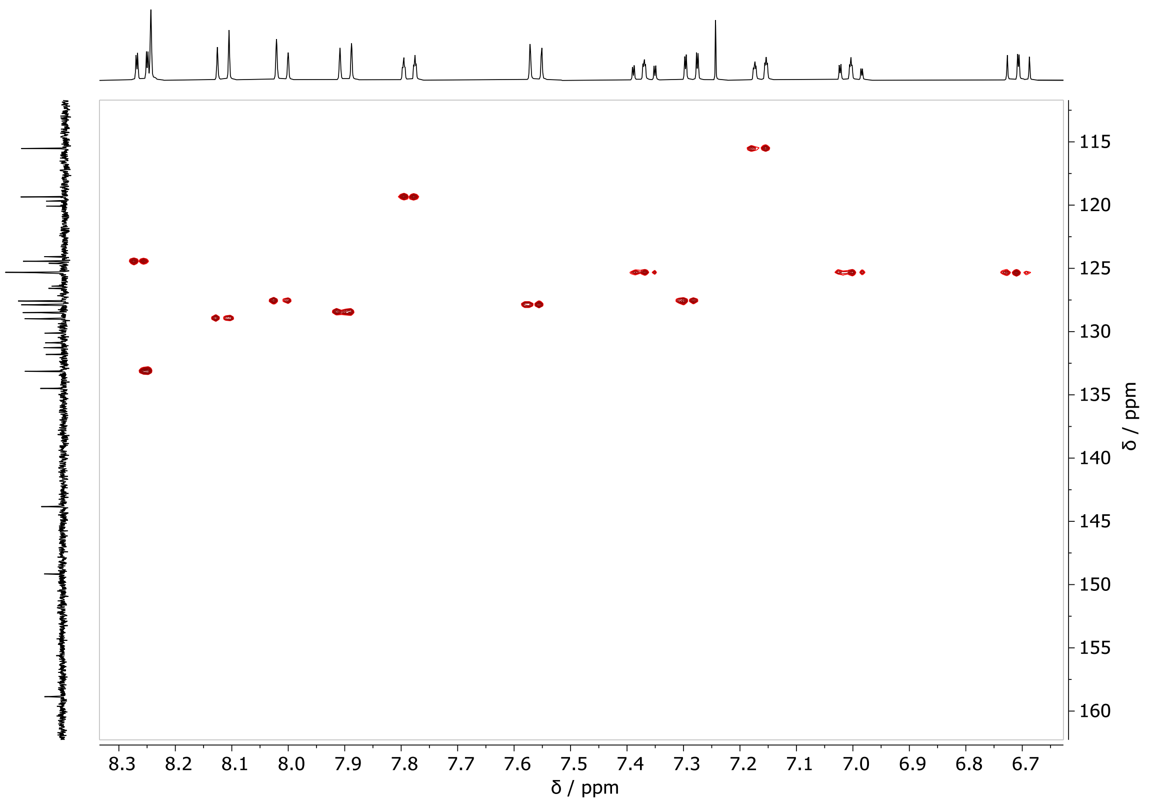

Figure S39. ^{1}H–^{13}C HSQC NMR spectrum of **[8]HBI-2** in CD_2Cl_2.

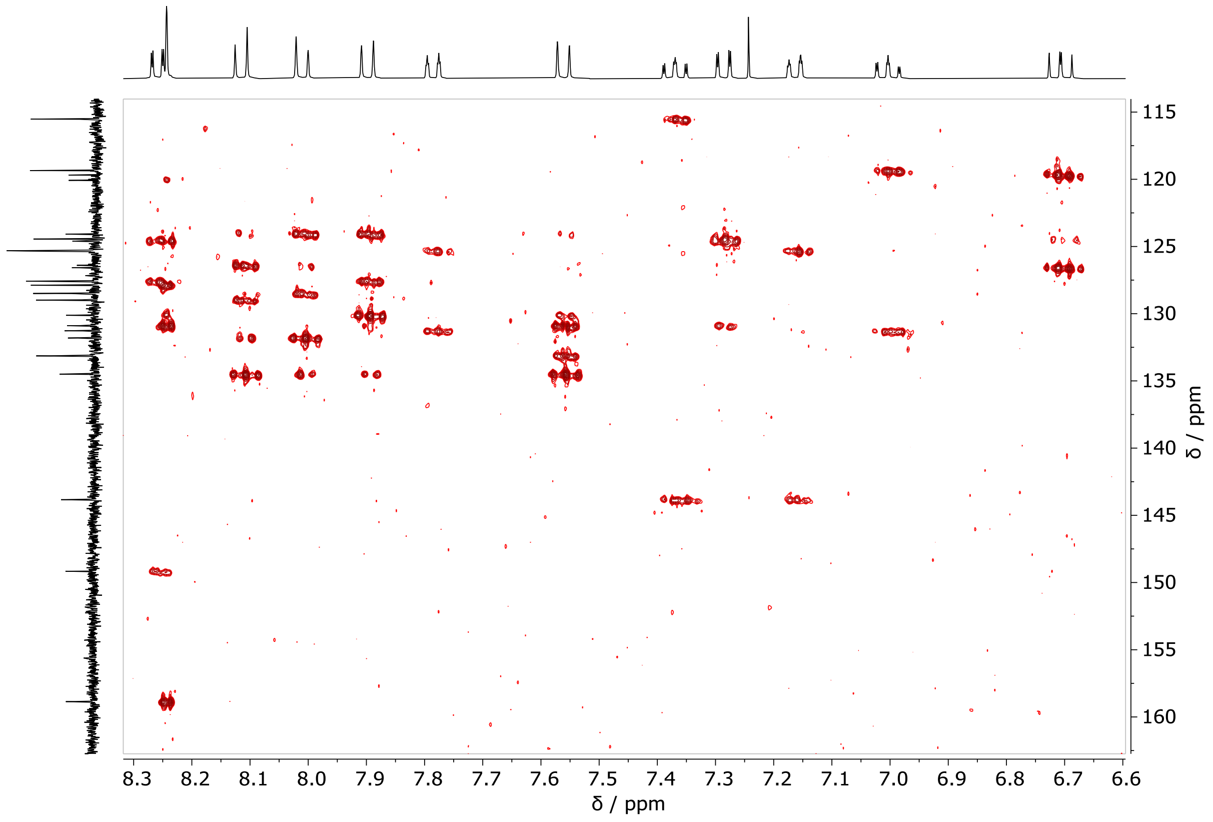

Figure S40. ^{1}H–^{13}C HMBC NMR spectrum of **[8]HBI-2** in CD_2Cl_2.

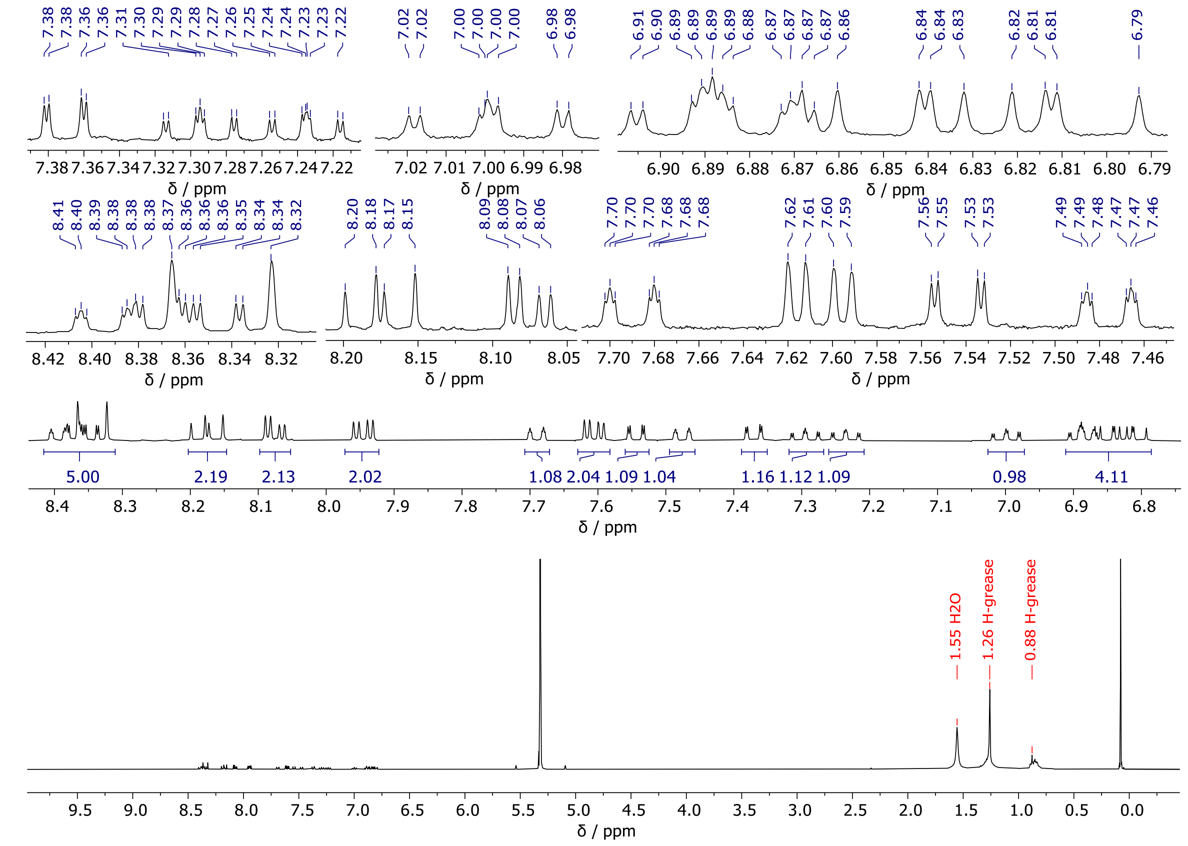

Figure S41. ^{1}H NMR spectra (400.1 MHz, CD_2Cl_2) of **[8]HBI-3**.

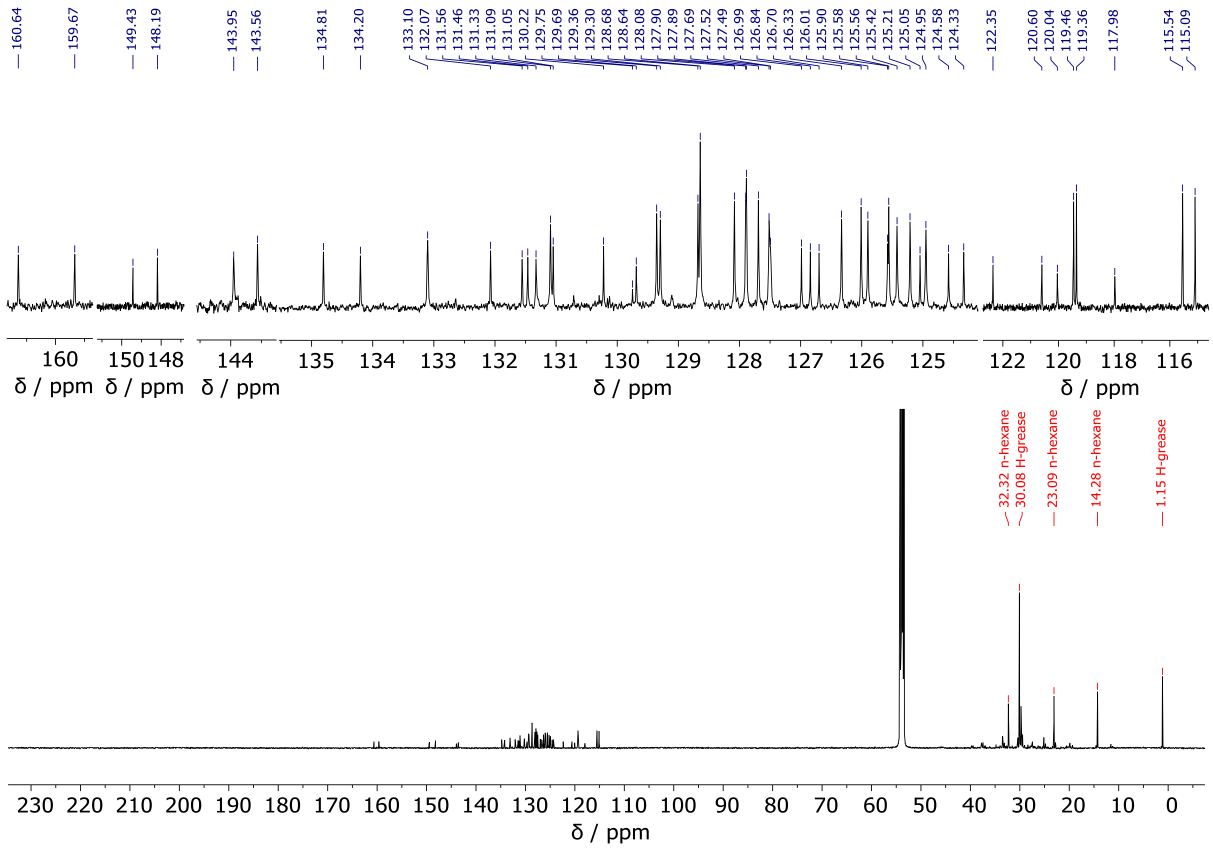

Figure S42. ^{13}C NMR spectra (150.9 MHz, CD_2Cl_2) of **[8]HBI-3**.

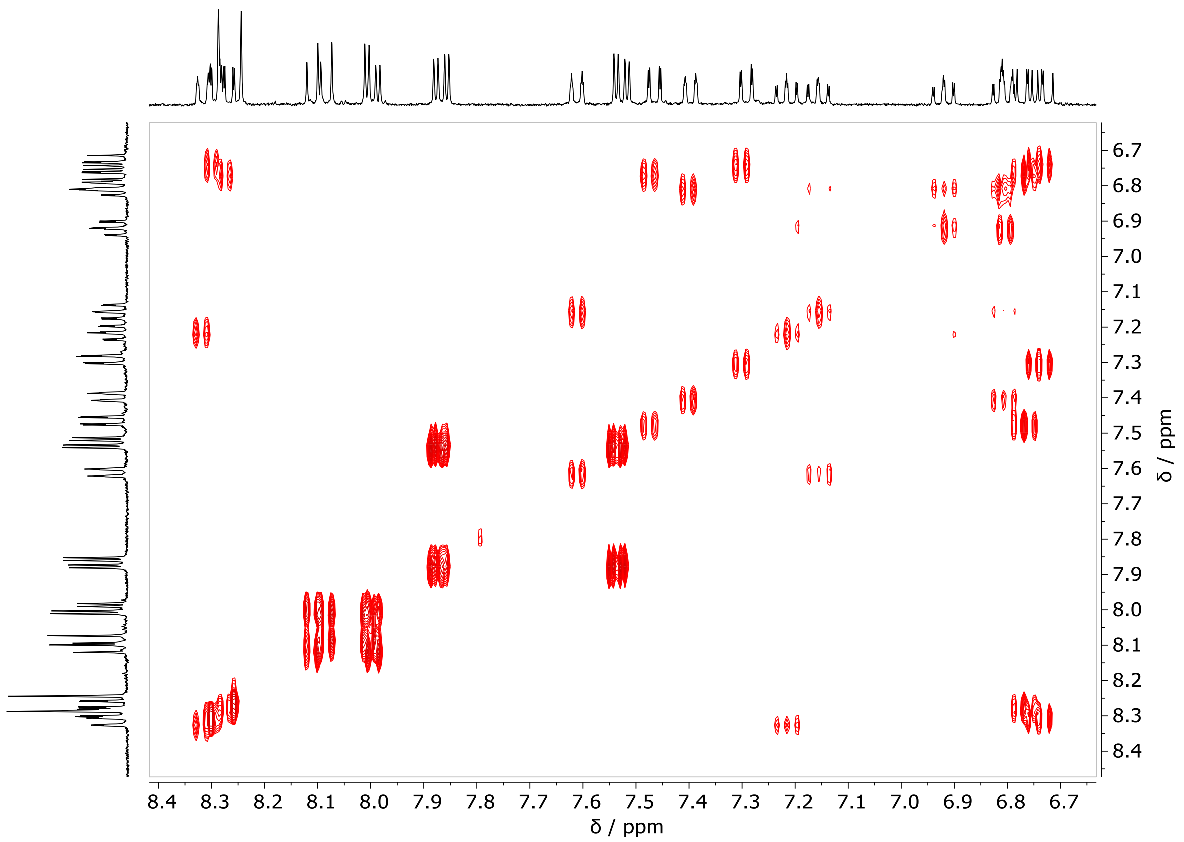

Figure S43. ^{1}H–^{1}H COSY NMR spectrum of **[8]HBI-3** in CD_2Cl_2.

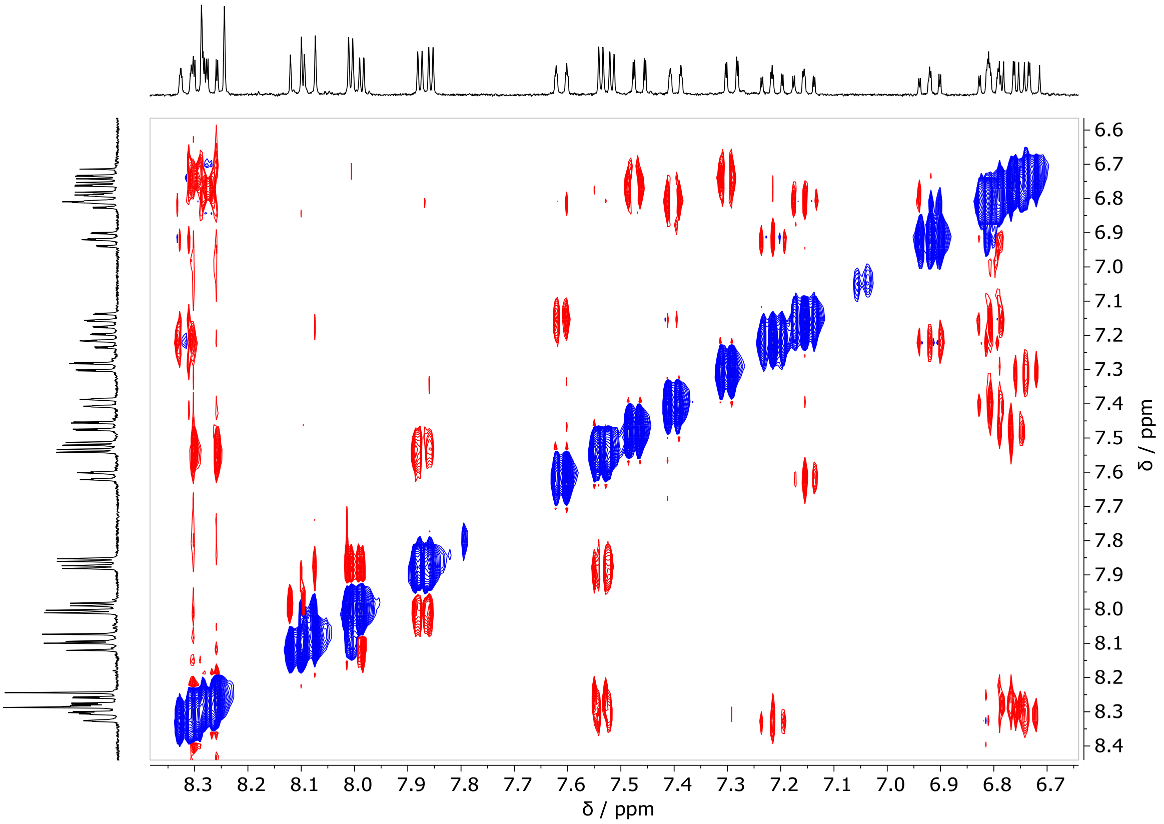

Figure S44. ^{1}H–^{1}H NOESY NMR spectrum of **[8]HBI-3** in CD_2Cl_2.

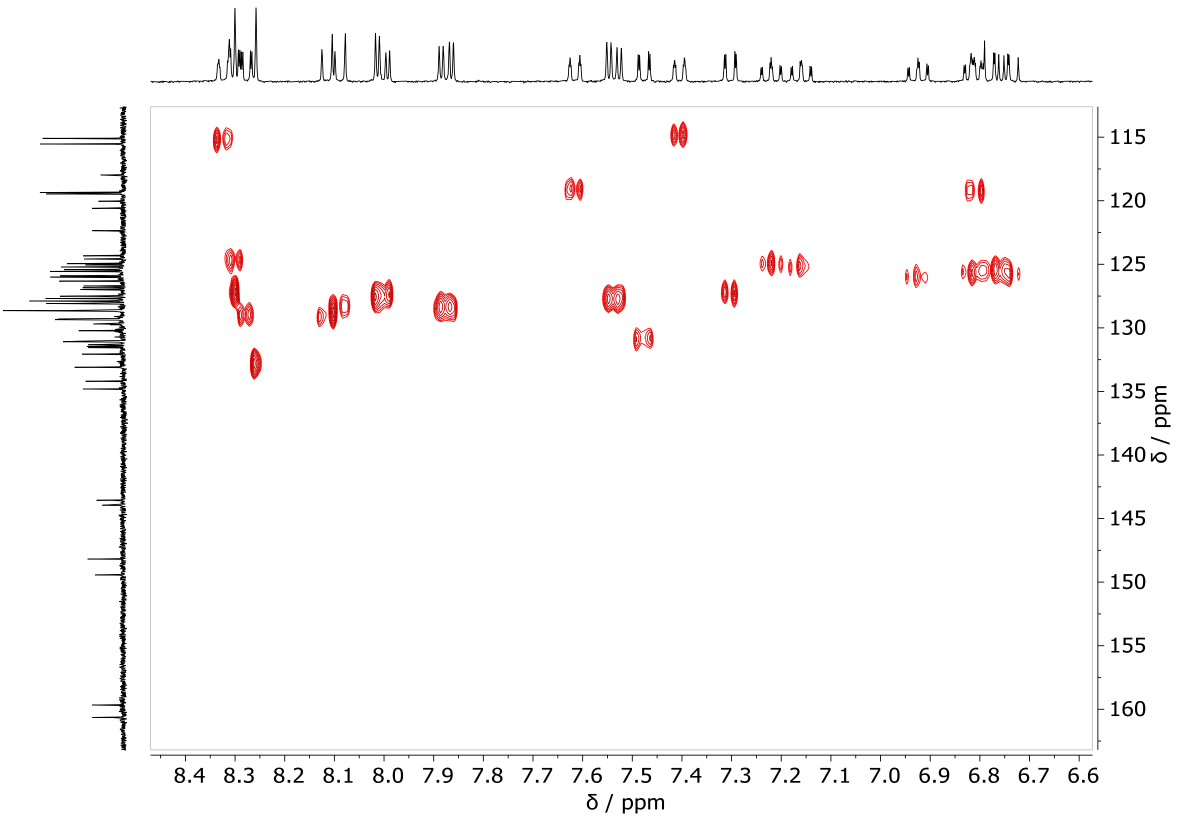

Figure S45. ^{1}H–^{13}C HSQC NMR spectrum of **[8]HBI-3** in CD_2Cl_2.

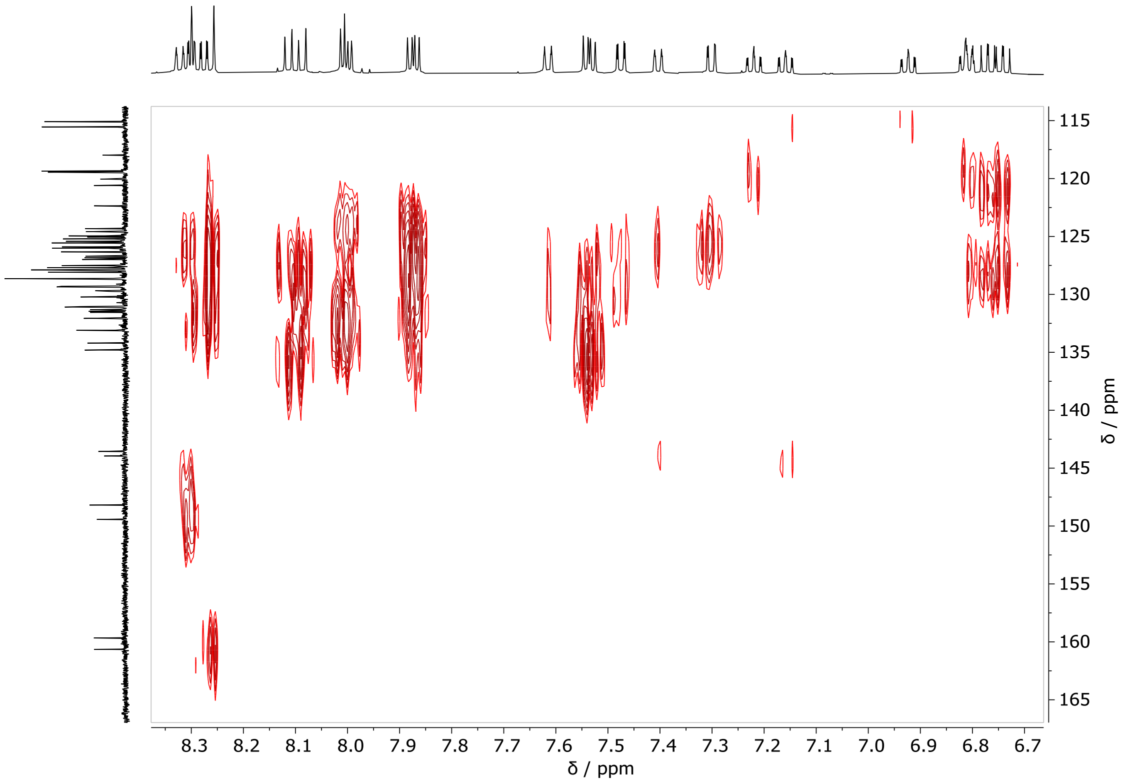

Figure S46. ^{1}H–^{13}C HMBC NMR spectrum of **[8]HBI-3** in CD_2Cl_2.

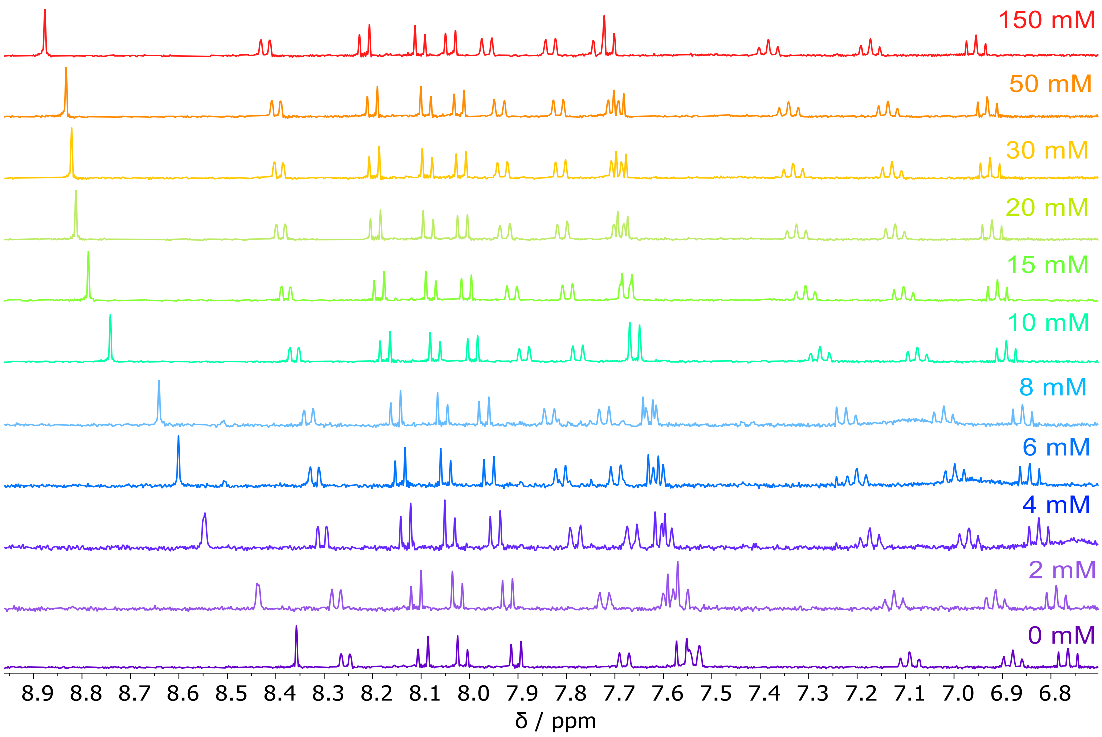

Figure S47. ^{1}H NMR spectral changes of **[8]HBI-1** (c~6 mM) upon addition of TFA-d in CD_2Cl_2.

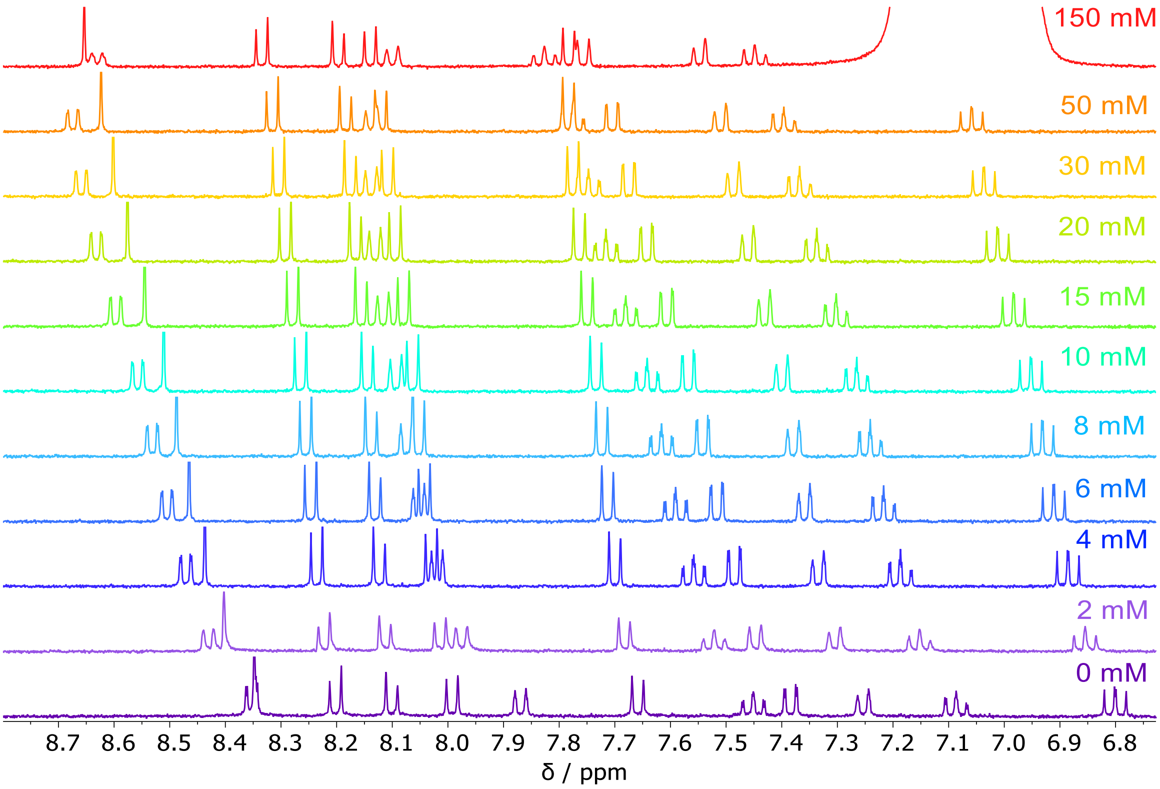

Figure S48. ^{1}H NMR spectral changes of **[8]HBI-2** (c~6 mM) upon addition of TFA-d in CD_2Cl_2.

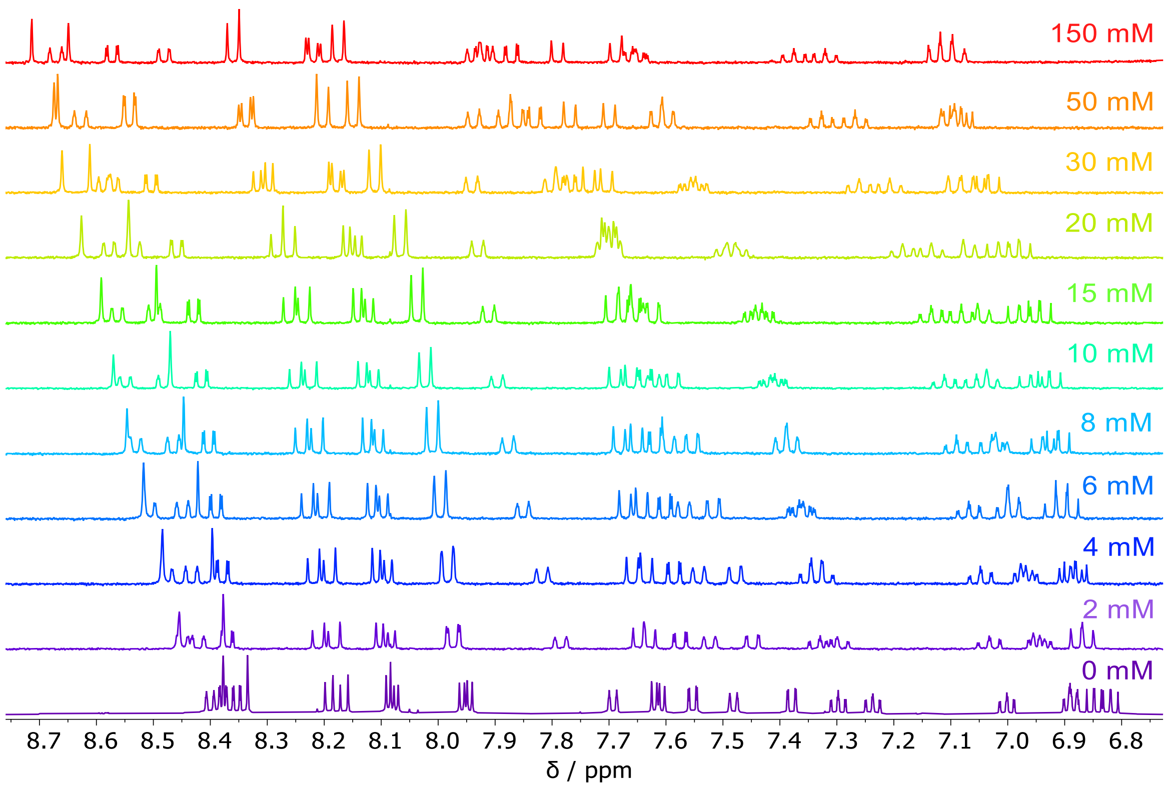

Figure S49. ^{1}H NMR spectral changes of **[8]HBI-3** (c~6 mM) upon addition of TFA-d in CD_2Cl_2.

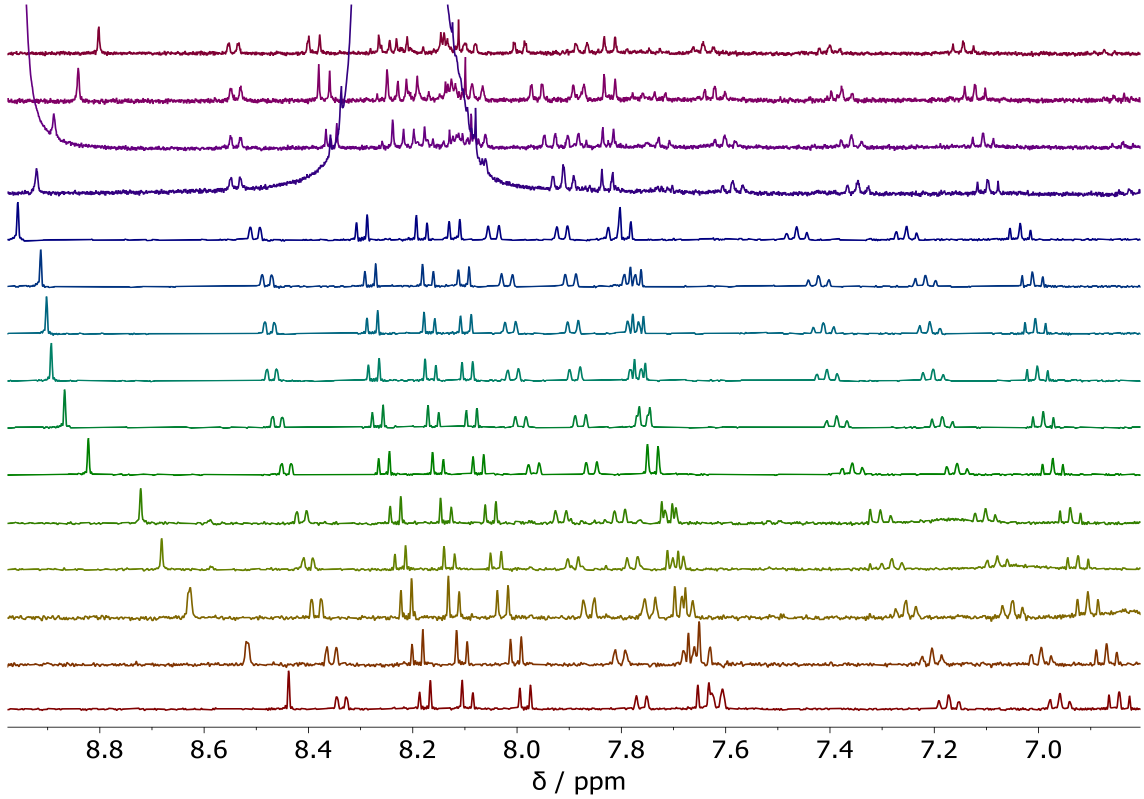

Figure S50. ^{1}H NMR spectral changes of **[8]HBI-1** (c~6 mM) upon addition of TFA-d from 0 mM to 2.0 M (bottom to top) in CD_2Cl_2.

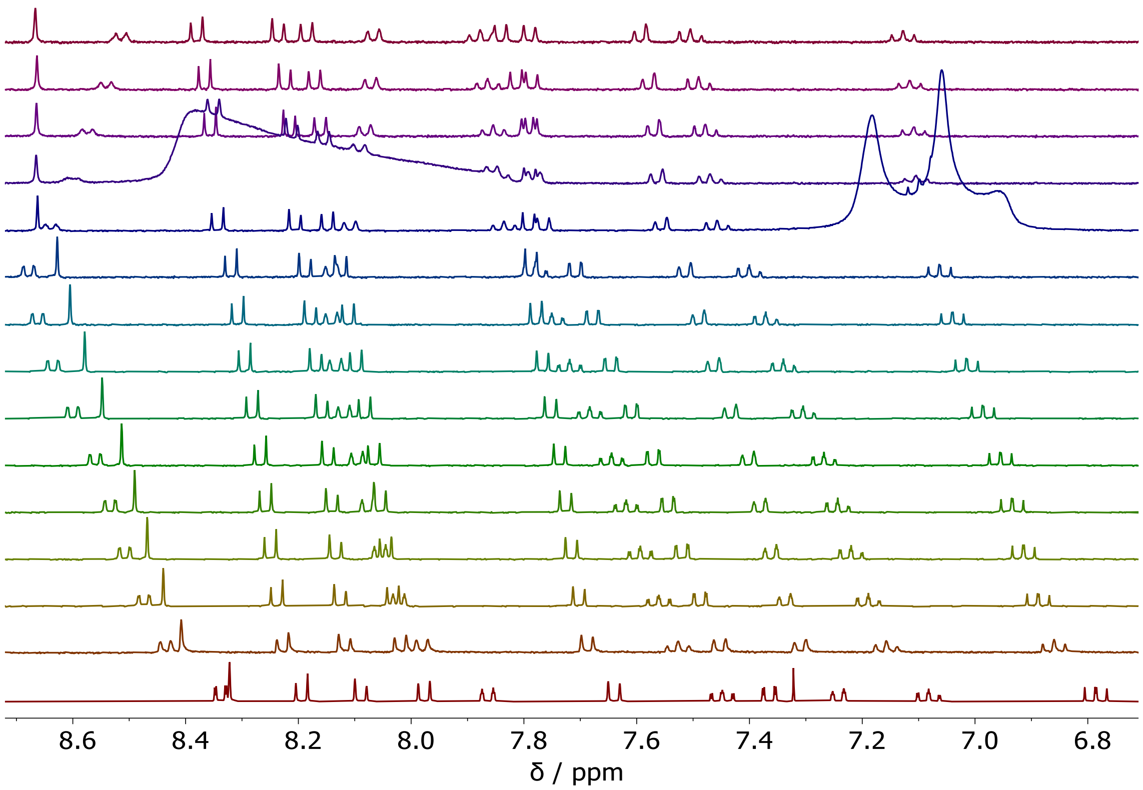

Figure S51. ^{1}H NMR spectral changes of **[8]HBI-2** (c~6 mM) upon addition of TFA-d from 0 mM to 2.0 M (bottom to top) in CD_2Cl_2.

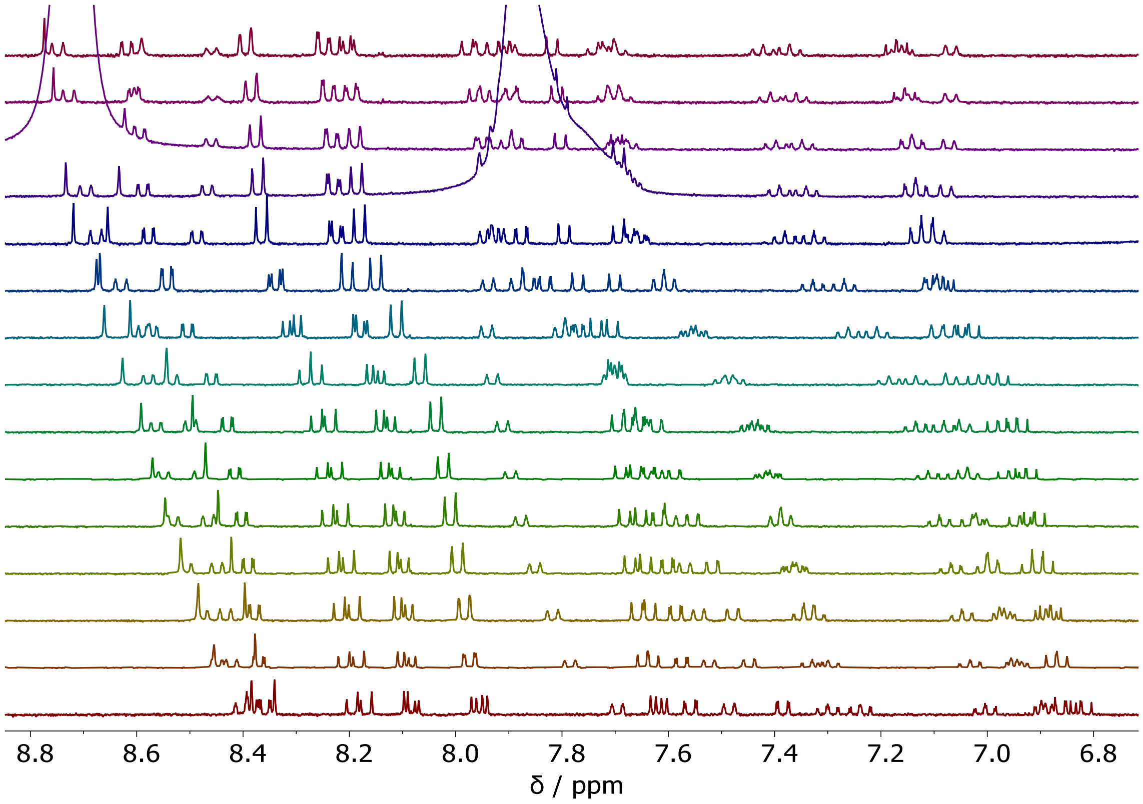

Figure S52. ^{1}H NMR spectral changes of **[8]HBI-3** (c~6 mM) upon addition of TFA-d from 0 mM to 2.0 M (bottom to top) in CD_2Cl_2.

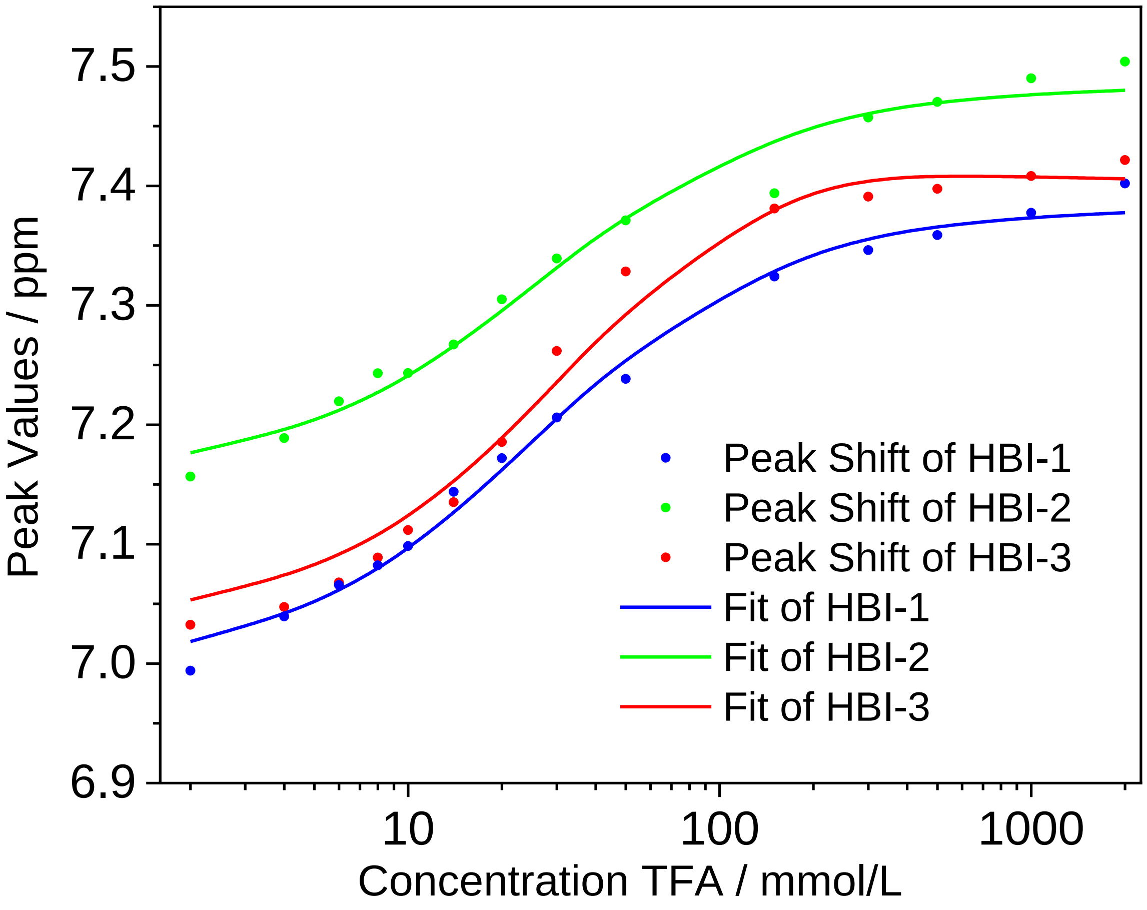

Figure S53. Plot of the NMR shifts of the benzene ring positions 3 and 4 of benzimidazole resulting from the TFA titration shown in **Figure S50** to **Figure S52**.

Table S8. Association constants for the diprotonation of the **[8]HBI** compounds with BindFit (Fitter: NMR 1:2).[20-21] The Fits are shown in **Figure S53**.

Compound	**HBI-1-H_2^{2+}**	**HBI-2- H_2^{2+}**	**HBI-3- H_2^{2+}**
K [M^{-1}]	8.257 x 10^{-2}	7.989 x 1^{-2}	3.021 x 10^{-2}

S10. High-resolution mass spectrometry

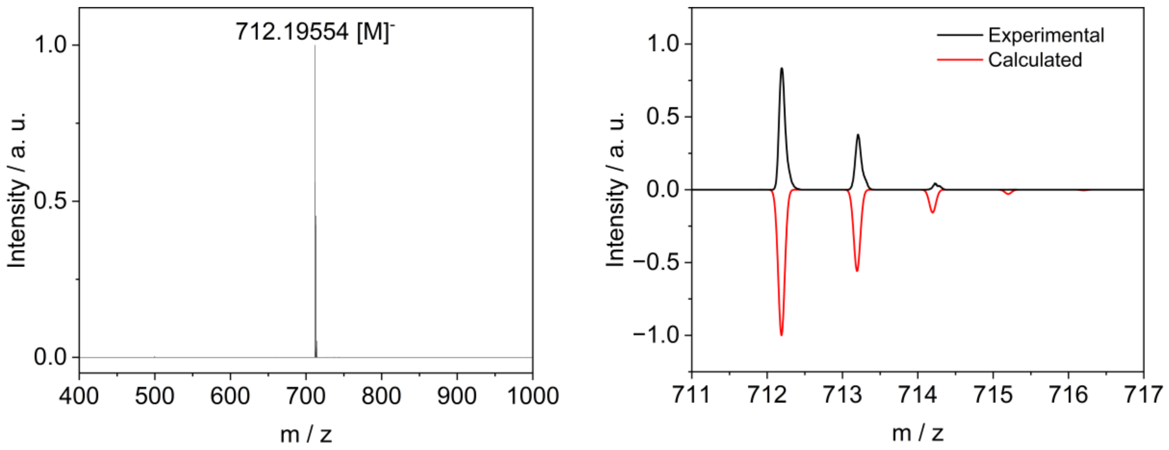

Figure S54. MALDI-TOF HRMS of **[8]HBI-1**.

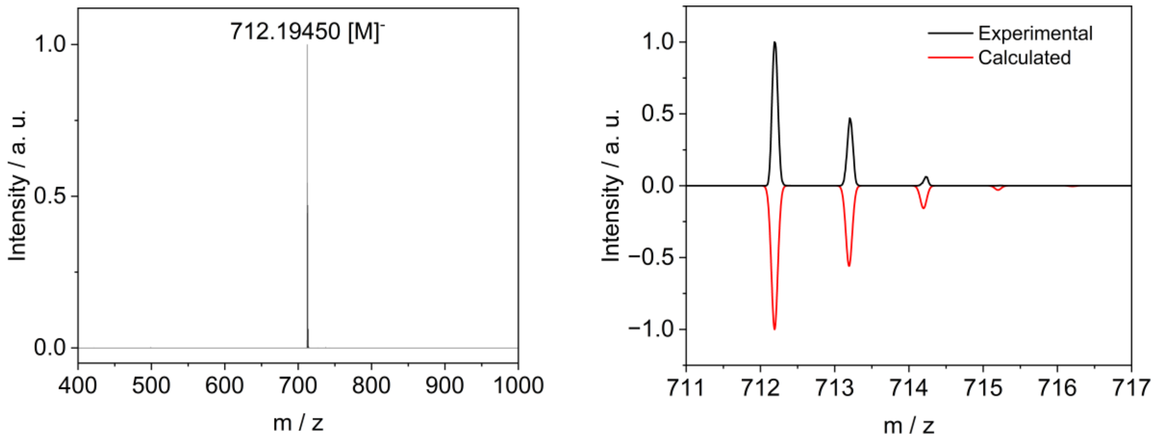

Figure S55. MALDI-TOF HRMS of **[8]HBI-2**.

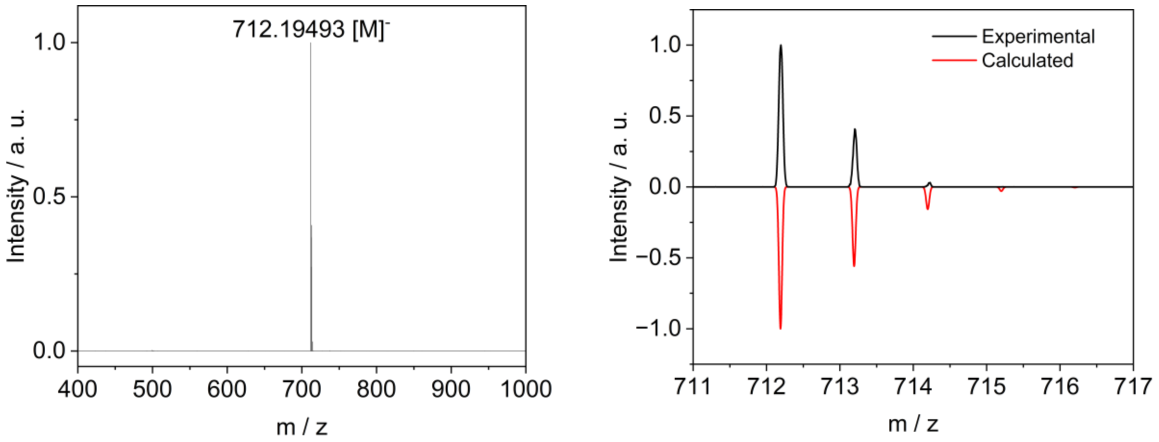

Figure S56. MALDI-TOF HRMS of **[8]HBI-3**.

S11. Cartesian coordinates

The cartesian coordinates for the discussed compounds in the neutral state, radical anions and dicationic state are uploaded as separate files.

S12. References

[1] F. Saal, V. Brancaccio, K. Radacki, H. Braunschweig, P. Ravat, *Angew. Chem. Int. Ed.* **2025**, *64*, e202508779.

[2] M. J. Frisch, G. W. Trucks, H. B. Schlegel, G. E. Scuseria, M. A. Robb, J. R. Cheeseman, G. Scalmani, V. Barone, G. A. Petersson, H. Nakatsuji, X. Li, M. Caricato, A. V. Marenich, J. Bloino, B. G. Janesko, R. Gomperts, B. Mennucci, H. P. Hratchian, J. V. Ortiz, A. F. Izmaylov, J. L. Sonnenberg, Williams, F. Ding, F. Lipparini, F. Egidi, J. Goings, B. Peng, A. Petrone, T. Henderson, D. Ranasinghe, V. G. Zakrzewski, J. Gao, N. Rega, G. Zheng, W. Liang, M. Hada, M. Ehara, K. Toyota, R. Fukuda, J. Hasegawa, M. Ishida, T. Nakajima, Y. Honda, O. Kitao, H. Nakai, T. Vreven, K. Throssell, J. A. Montgomery Jr., J. E. Peralta, F. Ogliaro, M. J. Bearpark, J. J. Heyd, E. N. Brothers, K. N. Kudin, V. N. Staroverov, T. A. Keith, R. Kobayashi, J. Normand, K. Raghavachari, A. P. Rendell, J. C. Burant, S. S. Iyengar, J. Tomasi, M. Cossi, J. M. Millam, M. Klene, C. Adamo, R. Cammi, J. W. Ochterski, R. L. Martin, K. Morokuma, O. Farkas, J. B. Foresman, D. J. Fox, Wallingford, CT, **2016**.

[3] L. Falivene, L. Cavallo, G. Talarico, *ACS Catal.* **2015**, *5*, 6815–6822.

[4] E. F. Pettersen, T. D. Goddard, C. C. Huang, E. C. Meng, G. S. Couch, T. I. Croll, J. H. Morris, T. E. Ferrin, *Protein Science.* **2021**, *30*, 70–82.

[5] A. J. Schaefer, V. M. Ingman, S. E. Wheeler, *J. Comput. Chem.* **2021**, *42*, 1750–1754.

[6] N. Saleh, B. Moore II, M. Srebro, N. Vanthuyne, L. Toupet, J. A. G. Williams, C. Roussel, K. K. Deol, G. Muller, J. Autschbach, J. Crassous, *Chem. Eur. J.* **2015**, *21*, 1673–1681.

[7] H. Sakai, T. Kubota, J. Yuasa, Y. Araki, T. Sakanoue, T. Takenobu, T. Wada, T. Kawai, T. Hasobe, *Org. Biomol. Chem.* **2016**, *14*, 6738–6743.

[8] S. Pascal, C. Besnard, F. Zinna, L. Di Bari, B. Le Guennic, D. Jacquemin, J. Lacour, *Org. Biomol. Chem.* **2016**, *14*, 4590–4594.

[9] T. Otani, A. Tsuyuki, T. Iwachi, S. Someya, K. Tateno, H. Kawai, T. Saito, K. S. Kanyiva, T. Shibata, *Angew. Chem. Int. Ed.* **2017**, *56*, 3906–3910.

[10] E. Yen-Pon, F. Buttard, L. Frédéric, P. Thuéry, F. Taran, G. Pieters, P. A. Champagne, D. Audisio, *JACS Au* **2021**, *1*, 807–818.

[11] K. Hanada, J. Nogami, K. Miyamoto, N. Hayase, Y. Nagashima, Y. Tanaka, A. Muranaka, M. Uchiyama, K. Tanaka, *Chem. Eur. J.* **2021**, *27*, 9313–9319.

[12] L. Guy, M. Mosser, D. Pitrat, J.-C. Mulatier, M. Kukułka, M. Srebro-Hooper, E. Jeanneau, A. Bensalah-Ledoux, B. Baguenard, S. Guy, *Molecules* **2023**, *28*, 7322.

[13] S. Miwa, D. Mizutani, K. Kawano, K. Matsuzaki, Y. Nagata, K. Tsubaki, K. Takasu, H. Takikawa, *Chem. Eur. J.* **2025**, *31*, e202500335.

[14] P. García-Cerezo, M. D. Codesal, A. H. G. David, L. Le Bras, S. Abid, X. Li, D. Miguel, M. Kazem-Rostami, B. Champagne, A. G. Campaña, J. F. Stoddart, V. Blanco, *Adv. Mater.* **2025**, *37*, 2417326.

[15] C. Maeda, Y. Daigen, S. Michishita, T. Ema, *Org. Lett.* **2025**, *27*, 6648–6653.

[16] R. Inoue, A. Aoki, T. Agou, Y. Morisaki, *Angew. Chem. Int. Ed.* **2025**, *64*, e202506733.

[17] G. M. Sheldrick, *Acta Crystallographica Section A* **2015**, *71*, 3–8.

[18] G. Sheldrick, *Acta Crystallographica Section A* **2008**, *64*, 112–122.

[19] C. F. Macrae, I. Sovago, S. J. Cottrell, P. T. A. Galek, P. McCabe, E. Pidcock, M. Platings, G. P. Shields, J. S. Stevens, M. Towler, P. A. Wood, *J. Appl. Cryst.* **2020**, *53*, 226–235.

[20] BindFit v0.5, Supramolecular | https://app.supramolecular.org/bindfit/, (accessed 24 April 2025).

[21] P. Thordarson, *Chem. Soc. Rev.* **2011**, *40*, 1305–1323.
